# Passive noise datasets at regolith sites

**DOI:** 10.1016/j.dib.2018.08.055

**Published:** 2018-08-31

**Authors:** Bambang Setiawan, Mark Jaksa, Michael Griffith, David Love

**Affiliations:** a Faculty of Engineering, Syiah Kuala University, Jl. Tgk. Syech Abdurrauf 7, Darussalam, Banda Aceh 23111, Indonesia; b School of Civil, Environmental and Mining Engineering, the University of Adelaide, North Terrace Campus, SA 5005, Australia; c Department of State Development, Government of South Australia, 101 Grenfell Street, Adelaide, SA 5000, Australia

**Keywords:** Passive noise, Array, HVSR, SPAC, Regolith

## Abstract

The data presented in this article contain datasets of passive noise measurements at regolith sites in Adelaide, South Australia. The data were acquired using three component (3C) LE-3Dlite Lennartz seismometers with an eigenfrequency of 1 Hz. The data were acquired at eight sites across Adelaide׳s regolith in a hexagonal array layout. Four tests, each with a duration of 30 min, were conducted at different times. The ambient noise data can be used for both horizontal to vertical spectral ratio (HVSR) analysis and array analyses, which are essential to obtain the site fundamental frequency and the ellipticity of the fundamental mode Rayleigh waves at the measured site. The array analyses are useful to obtain the dispersion curves, which are needed to estimate the shear wave velocity profile.

**Specifications Table**

**Table:** 

Subject area	*Geophysics*
More specific subject area	*Near surface geophysics*
Type of data	*Table, text file, and figure*
How data was acquired	*3 component (3C) LE-3Dlite Lennartz seismometers with an eigenfrequency of 1 Hz*
Data format	*Raw, filtered, analyzed*
Experimental factors	*The passive noise measurements were conducted for at least 2 h at a sampling frequency of 100 Hz*
Experimental features	*The 3C LE-3Dlite Lennartz seismometers were equipped with an analog-to-digital recorder & a global positioning system (GPS)*
Data source location	*Adelaide City, South Australia*
Data accessibility	*Data are included with this article*

**Value of the data**

• The ambient noise data can be used in the development of further experiments at other regolith sites
• The data can be compared to other measurements for provide greater insight
• The HVSR curves serve as a benchmark for other researchers
• The data are important to evaluate the reliability of ambient vibration data analysis and for comparison of other sites with similar or divergent geophysical characteristics.

## 1 Data

**Fig. 1:**
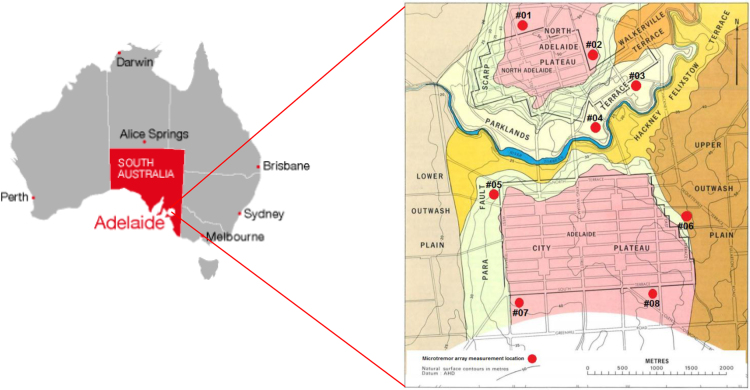
Locality of the collected data sites [5].

The data in this article contain a series of measurements of ambient noise (microtremor) at regolith sites in Adelaide, South Australia (Fig. 1). Eight sites across Adelaide׳s regolith were measured in a hexagonal array layout. The acquired data contains four continuous ambient noise tests, each with a duration of 30 min. The ambient noise data, which were acquired in arrays consisting of three instruments in a triangular arrangement, are important for horizontal to vertical spectral ratio (HVSR) analyses. The HVSR method was introduced by [1] based on the work of [2]. This HVSR method was popularized by [3]. Due to its simplicity this HVSR method has been used extensively since 1989. The data from all seismometers in the hexagonal array can be used to evaluate the dispersion curves in array analyses, such as spatial autocorrelation (SPAC) analysis [4]. HVSR analyses are carried out to obtain the site fundamental frequency and ellipticity of the fundamental mode Rayleigh waves at each of the measured sites. The use of the array analyses to evaluate the dispersion curve is motivated by the objective of analyzing Raleigh waves and excluding Love waves.

## 2 Experimental design, equipment and analyses

### 2.1 Experimental design

**Fig. 2:**
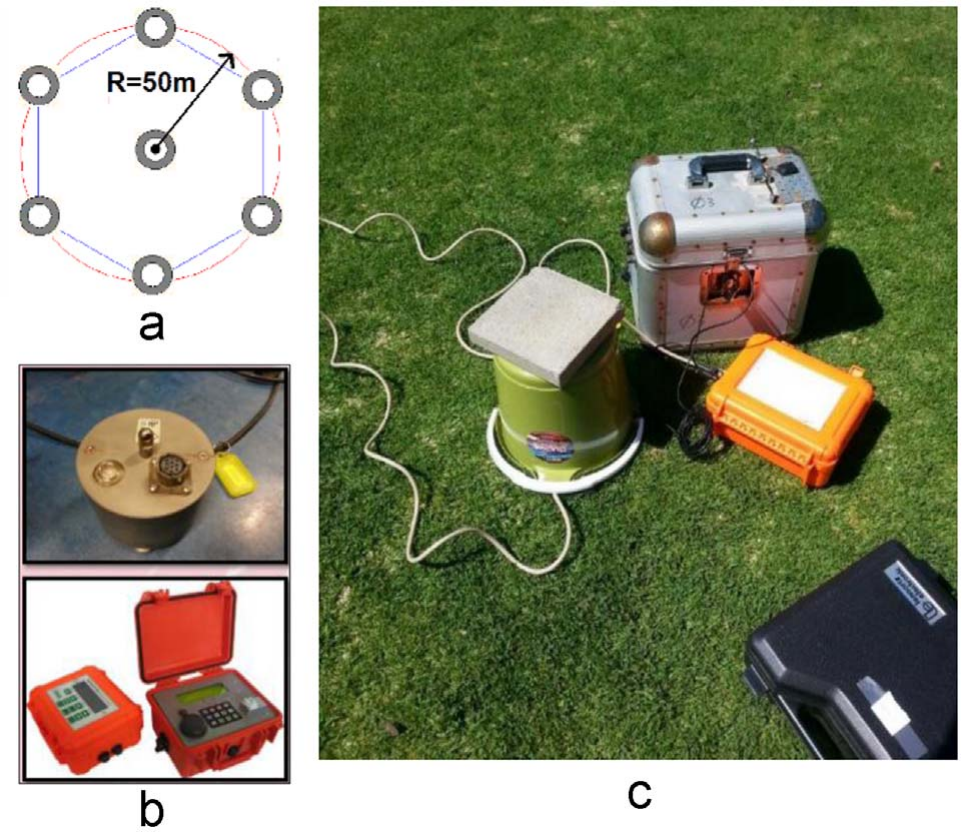
(a) Array layout; (b) LE3DLite Lennartz seismometer Kelunji Data Recorder; and (c) field setting up of the equipment.

A hexagonal array with a radius of 50 m was used in the measurement process, as shown in Fig. 2*(a).* Eight ambient noise field measurements were conducted in the parklands that surround the Adelaide city area (Fig. 1) using seven sets of 3-component seismometers. These seismometers record the three orthogonal components of vibration: two horizontals (i.e. east–west and north–south) and one vertical. The data were recorded and saved to the internal memory storage within each instrument.

### 2.2 Equipment

All the seismometers used for the noise data acquisition are three component (3C) LE-3Dlite Lennartz seismometers with an eigenfrequency of 1 Hz, as shown in Fig. 2(b). These 3C seismometers were connected to an analog-to-digital recorder [Kelunji digital data recorder, as shown in Fig. 2(b)], a global positioning system (GPS), antenna and a battery. The digital data recorder was equipped with a memory disk to store the acquired data. A laptop computer was used for initial setting up and checking the status of the recording process.

In order to be provide a stable measurement platform at each site, the seismometers were placed onto of a 20 mm thick circular concrete slab over a generally firm to a stiff ground surface, which was previously cleared of vegetation and any pebble-sized rubble. The seismometers were leveled by adjusting the legs so as to minimize any instability during recording. For consistency with the measured horizontal vibration, all the seismometers were also oriented to magnetic North. Furthermore, to maintain the stability of the seismometer during the data acquisition, all the seismometers were protected from wind-induced vibrations by means of a plastic container and stabilized with a paving block on top, as shown in Fig. 2(c).

### 2.3 Equipment repeatability

**Fig. 3:**
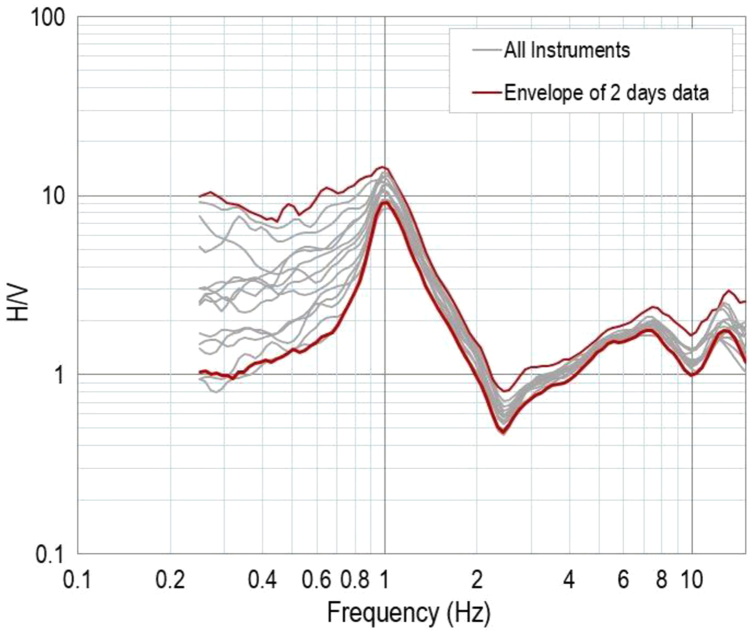
HVSR curves of all adopted seismometers and the envelope of HVSR test results from two continuous days of measurement over 30-min time periods.

Prior to data acquisition, all of the equipment sets were examined to assess their repeatability. All equipment sets were run simultaneously at the same location, with a separation distance of approximately 0.5 m from one another for at least two days. The results of this huddle test are presented in Table 1 (where f_0_ is the fundamental frequency; n is the number of windows selected for the average HVSR curve; *A*_0_ is the HVSR peak amplitude at frequency f_0_; and i, ii, and iii are the criteria suggested by [6] to assess the reliability of the HVSR curves. The HVSR curves from the huddle tests that passed the SESAME reliability criteria, were plotted with respect to HVSR amplitude and frequency. The results are presented in Fig. 3, which demonstrates very similar curves, particularly between 0.8 and 10.0 Hz. A high dispersion is shown from a frequency of 0.8 Hz, downward to 0.25 Hz, which suggests a low noise source intensity over this frequency interval. Further investigation of this huddle test is available in [7].

**Table 1:** Fundamental frequency, maximum horizontal vertical spectral ratio, number of stationary data and SESAME reliability criteria checks.

**No.**	**Date**		**Hour**		***f***_**0**_	***n***	***A***_**0**_	**Check for reliability**		
	**GMT**	**Adelaide**	**GMT**	**Adelaide**				i	ii	iii
1	2/10/2014	2/10/2014	6:31:00 a.m.	3:01:00 p.m.	0.90	13	12.82	PASS	PASS	PASS
2			7:00:00 a.m.	3:30:00 p.m.	0.88	12	9.92	PASS	PASS	FAIL
3			7:30:00 a.m.	4:00:00 p.m.	0.99	16	11.42	PASS	PASS	FAIL
4			8:00:00 a.m.	4:30:00 p.m.	1.00	20	11.68	PASS	PASS	PASS
5			8:30:00 a.m.	5:00:00 p.m.	0.94	22	11.18	PASS	PASS	PASS
6			9:00:00 a.m.	5:30:00 p.m.	0.98	23	12.63	PASS	PASS	PASS
7			9:30:00 a.m.	6:00:00 p.m.	0.98	25	13.31	PASS	PASS	PASS
8			10:00:00 a.m.	6:30:00 p.m.	1.00	18	11.37	PASS	PASS	PASS
9			10:30:00 a.m.	7:00:00 p.m.	1.00	22	11.01	PASS	PASS	PASS
10			11:00:00 a.m.	7:30:00 p.m.	1.00	24	10.85	PASS	PASS	PASS
11			11:30:00 a.m.	8:00:00 p.m.	1.01	23	11.99	PASS	PASS	PASS
12			12:00:00 p.m.	8:30:00 p.m.	1.01	25	10.49	PASS	PASS	PASS
13			12:30:00 p.m.	9:00:00 p.m.	0.99	26	10.88	PASS	PASS	PASS
14			1:00:00 p.m.	9:30:00 p.m.	1.00	26	11.28	PASS	PASS	PASS
15			1:30:00 p.m.	10:00:00 p.m.	1.00	23	11.53	PASS	PASS	PASS
16			2:00:00 p.m.	10:30:00 p.m.	1.02	23	11.43	PASS	PASS	PASS
17			2:30:00 p.m.	11:00:00 p.m.	1.01	23	11.20	PASS	PASS	PASS
18			3:00:00 p.m.	11:30:00 p.m.	1.01	27	11.60	PASS	PASS	PASS
19		3/10/2014	3:30:00 p.m.	12:00:00 a.m.	1.01	25	11.11	PASS	PASS	PASS
20			4:00:00 p.m.	12:30:00 a.m.	1.02	25	11.63	PASS	PASS	PASS
21			4:30:00 p.m.	1:00:00 a.m.	1.00	24	11.06	PASS	PASS	PASS
22			5:00:00 p.m.	1:30:00 a.m.	1.02	21	10.75	PASS	PASS	PASS
23			5:30:00 p.m.	2:00:00 a.m.	0.99	23	11.35	PASS	PASS	PASS
24			6:00:00 p.m.	2:30:00 a.m.	1.00	19	11.49	PASS	PASS	PASS
25			6:30:00 p.m.	3:00:00 a.m.	1.01	21	11.01	PASS	PASS	PASS
26			7:00:00 p.m.	3:30:00 a.m.	1.01	23	11.62	PASS	PASS	PASS
27			7:30:00 p.m.	4:00:00 a.m.	1.02	24	12.42	PASS	PASS	PASS
28			8:00:00 p.m.	4:30:00 a.m.	1.01	23	12.35	PASS	PASS	PASS
29			8:30:00 p.m.	5:00:00 a.m.	1.01	25	11.45	PASS	PASS	PASS
30			9:00:00 p.m.	5:30:00 a.m.	1.00	21	9.54	PASS	PASS	PASS
31			9:30:00 p.m.	6:00:00 a.m.	0.97	28	11.52	PASS	PASS	FAIL
32			10:00:00 p.m.	6:30:00 a.m.	0.98	29	12.92	PASS	PASS	PASS
33			10:30:00 p.m.	7:00:00 a.m.	0.98	24	14.42	PASS	PASS	PASS
34			11:00:00 p.m.	7:30:00 a.m.	0.98	9	13.93	PASS	PASS	FAIL
35			11:30:00 p.m.	8:00:00 a.m.	0.96	17	12.30	PASS	PASS	PASS
36	3/10/2014		12:00:00 a.m	8:30:00 a.m.	0.97	15	12.21	PASS	PASS	PASS
37			12:30:00 a.m	9:00:00 a.m.	0.98	23	12.54	PASS	PASS	PASS
38			1:00:00 a.m	9:30:00 a.m.	0.94	14	13.56	PASS	PASS	PASS
39			1:30:00 a.m	10:00:00 a.m.	0.96	19	12.22	PASS	PASS	FAIL
40			2:00:00 a.m	10:30:00 a.m.	1.01	22	12.80	PASS	PASS	PASS
41			2:30:00 a.m	11:00:00 a.m.	1.03	16	12.55	PASS	PASS	PASS
42			3:00:00 a.m	11:30:00 a.m.	1.02	12	12.36	PASS	PASS	PASS
43			3:30:00 a.m	12:00:00 p.m.	1.03	18	11.80	PASS	PASS	PASS
44			4:00:00 a.m	12:30:00 p.m.	1.02	13	12.42	PASS	PASS	PASS
45			4:30:00 a.m	1:00:00 p.m.	1.02	15	11.13	PASS	PASS	PASS
46			5:00:00 a.m	1:30:00 p.m.	0.99	22	12.18	PASS	PASS	PASS
47			5:30:00 a.m	2:00:00 p.m.	1.02	15	9.32	PASS	PASS	PASS
48			6:00:00 a.m	2:30:00 p.m.	1.00	18	12.27	PASS	PASS	PASS
49			6:30:00 a.m	3:00:00 p.m.	0.91	21	12.88	PASS	PASS	PAS
50			7:00:00 a.m	3:30:00 p.m.	0.98	17	13.08	PASS	PASS	FAIL
51			7:30:00 a.m	4:00:00 p.m.	1.00	22	12.79	PASS	PASS	PASS
52			8:00:00 a.m	4:30:00 p.m.	1.01	20	11.67	PASS	PASS	PASS
53			8:30:00 a.m	5:00:00 p.m.	1.01	28	11.85	PASS	PASS	PASS
54			9:00:00 a.m	5:30:00 p.m.	1.01	25	12.74	PASS	PASS	PASS
55			9:30:00 a.m	6:00:00 p.m.	1.03	23	11.71	PASS	PASS	PASS
56			10:00:00 a.m	6:30:00 p.m.	1.02	20	11.48	PASS	PASS	PASS
57			10:30:00 a.m	7:00:00 p.m.	1.02	22	10.47	PASS	PASS	PASS
58			11:00:00 a.m	7:30:00 p.m.	1.01	27	11.27	PASS	PASS	PASS
59			11:30:00 a.m	8:00:00 p.m.	1.01	20	10.56	PASS	PASS	PASS
60			12:00:00 p.m.	8:30:00 p.m.	1.01	27	10.36	PASS	PASS	PASS
61			12:30:00 p.m.	9:00:00 p.m.	1.00	23	10.20	PASS	PASS	PASS
62			1:00:00 p.m.	9:30:00 p.m.	1.02	22	10.55	PASS	PASS	PASS
63			1:30:00 p.m.	10:00:00 p.m.	1.00	29	10.91	PASS	PASS	PASS
64			2:00:00 p.m.	10:30:00 p.m.	1.00	31	10.10	PASS	PASS	PASS
65			2:30:00 p.m.	11:00:00 p.m.	1.02	25	9.16	PASS	PASS	PASS
66			3:00:00 p.m.	11:30:00 p.m.	1.01	29	10.01	PASS	PASS	PASS
67		4/10/2014	3:30:00 p.m.	12:00:00 a.m.	1.01	29	9.22	PASS	PASS	PASS
68			4:00:00 p.m.	12:30:00 a.m.	1.01	23	9.82	PASS	PASS	PASS
69			4:30:00 p.m.	1:00:00 a.m.	1.01	29	10.52	PASS	PASS	PASS
70			5:00:00 p.m.	1:30:00 a.m.	1.02	28	10.60	PASS	PASS	PASS
71			5:30:00 p.m.	2:00:00 a.m.	1.03	29	9.97	PASS	PASS	PASS
72			6:00:00 p.m.	2:30:00 a.m.	1.02	18	10.68	PASS	PASS	PASS
73			6:30:00 p.m.	3:00:00 a.m.	1.02	29	11.89	PASS	PASS	PASS
74			7:00:00 p.m.	3:30:00 a.m.	1.02	22	10.69	PASS	PASS	PASS
75			7:30:00 p.m.	4:00:00 a.m.	1.03	28	11.98	PASS	PASS	PASS
76			8:00:00 p.m.	4:30:00 a.m.	1.02	26	11.26	PASS	PASS	PASS
77			8:30:00 p.m.	5:00:00 a.m.	1.00	30	12.11	PASS	PASS	PASS
78			9:00:00 p.m.	5:30:00 a.m.	1.00	29	11.86	PASS	PASS	PASS
79			9:30:00 p.m.	6:00:00 a.m.	1.01	30	12.40	PASS	PASS	PASS
80			10:00:00 p.m.	6:30:00 a.m.	0.97	19	13.45	PASS	PASS	FAIL
81			10:30:00 p.m.	7:00:00 a.m.	1.01	23	11.51	PASS	PASS	PASS
82			11:00:00 p.m.	7:30:00 a.m.	1.01	19	11.79	PASS	PASS	PASS
83			11:30:00 p.m.	8:00:00 a.m.	0.95	18	13.19	PASS	PASS	PASS
84	4/10/2014		12:00:00 a.m.	8:30:00 a.m.	0.93	16	11.51	PASS	PASS	PASS
85			12:30:00 a.m.	9:00:00 a.m.	0.33	16	13.58	PASS	PASS	FAIL
86			1:00:00 a.m.	9:30:00 a.m.	0.33	8	18.05	PASS	FAIL	FAIL
87			1:30:00 a.m.	10:00:00 a.m.	0.97	12	11.29	PASS	PASS	FAIL
88			2:00:00 a.m.	10:30:00 a.m.	0.99	14	12.12	PASS	PASS	PASS
89			2:30:00 a.m.	11:00:00 a.m.	1.00	15	12.65	PASS	PASS	PASS
90			3:00:00 a.m.	11:30:00 a.m.	1.00	19	11.55	PASS	PASS	PASS
91			3:30:00 a.m.	12:00:00 p.m.	1.02	17	11.99	PASS	PASS	PASS
92			4:00:00 a.m.	12:30:00 p.m.	1.00	22	11.91	PASS	PASS	PASS
93			4:30:00 a.m.	1:00:00 p.m.	1.01	16	12.84	PASS	PASS	PASS
94			5:00:00 a.m.	1:30:00 p.m.	1.00	16	13.72	PASS	PASS	PASS
95			5:30:00 a.m.	2:00:00 p.m.	1.00	23	13.18	PASS	PASS	PASS
96			6:00:00 a.m.	2:30:00 p.m.	1.02	17	13.12	PASS	PASS	PASS

### 2.4 Detection of industrial origin

**Fig. 4:**
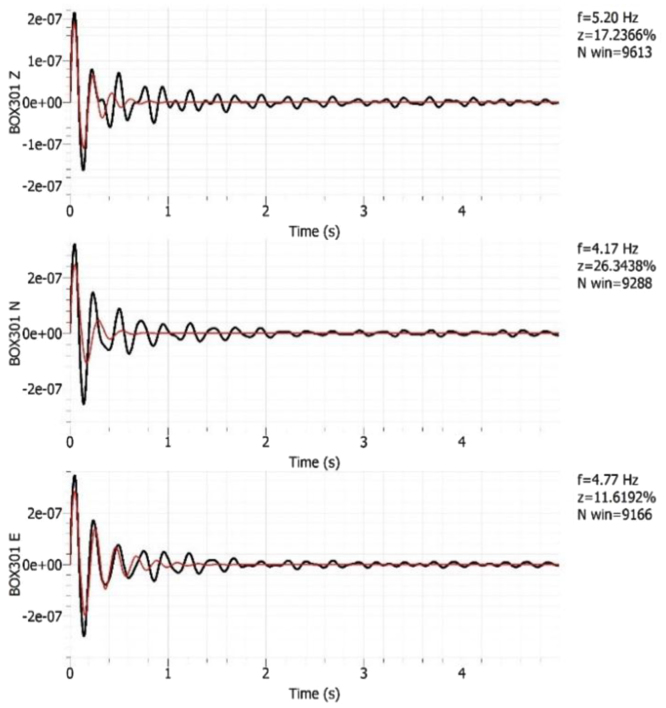
Example results of identification of data from an industrial source.

The Geopsy [8] damping toolbox was used to detect the presence of any vibration originating from an industrial source. Data from an industrial origin is concluded if the damping is much lower than 1% and the frequency is sustained. This identification is important in the HVSR analysis. Example results are shown in Fig. 4. Further details of all such analyses are included in Appendix A of the Supplementary Data associated with this paper.

### 2.5 Field data and Analyses

#### 2.5.1 Field data

**Fig. 5:**
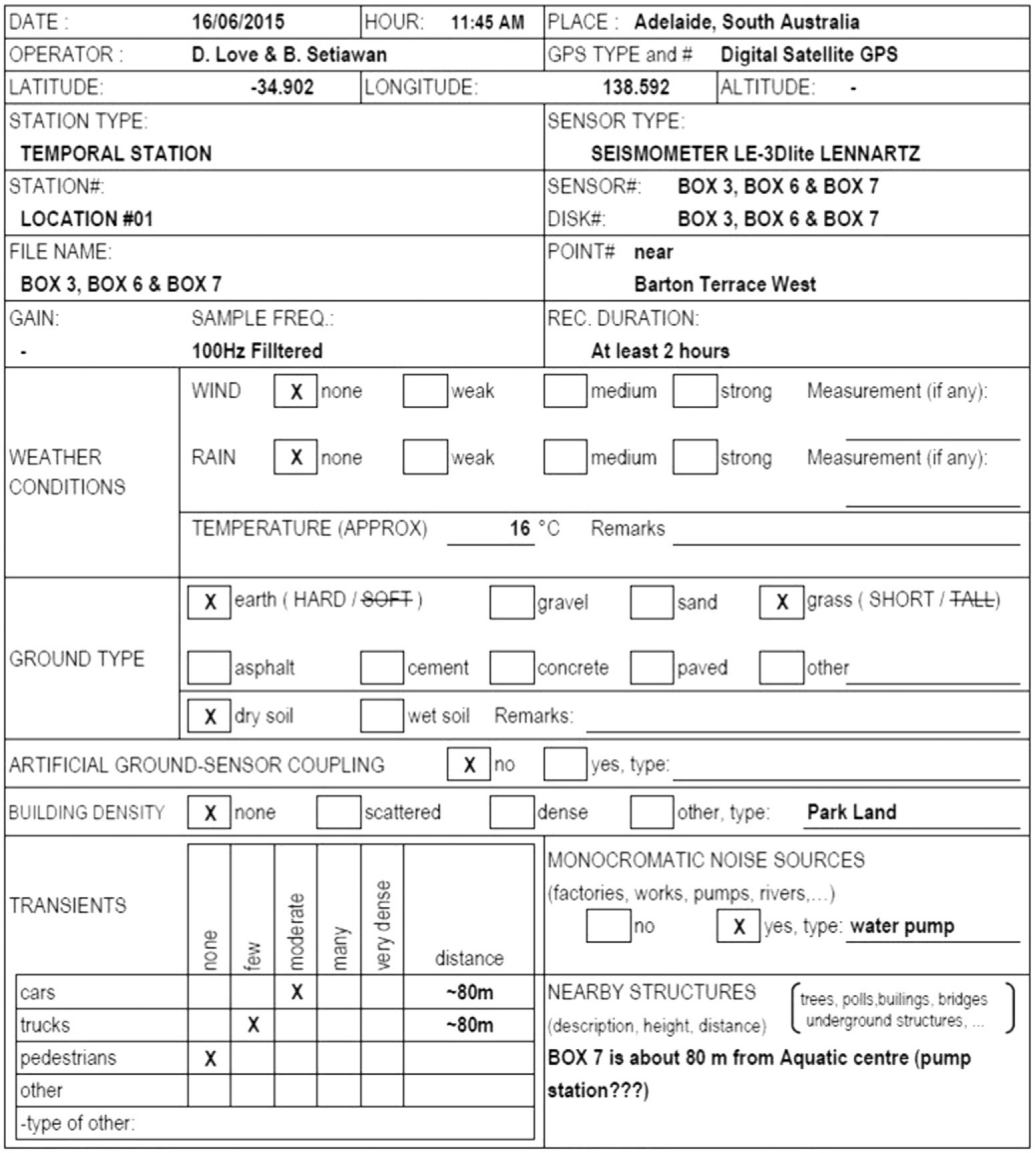
Example of a field data sheet.

At each survey location, noise measurements were conducted for at least 2 h, at a sampling frequency of 100 Hz. Field data were filtered using a cutoff frequency of 50 Hz. Only recordings on three instruments (in a triangular arrangement) were used for the horizontal to vertical spectral ratio (HVSR) analysis and the vertical seismometer readings from all instruments were used to analyze the spatial autocorrelation (SPAC). At each of the 8 sites, four tests, with a duration of 30 min, were conducted at different times using all instruments. An example of the field data sheet is shown in Fig. 5. All the field data sheets are included in [9].

#### 2.5.2 HVSR analysis

Analysis of the spectral ratio, between the Fourier amplitude spectrum of the horizontal (H) and vertical (V) components of the ambient noise, is fundamental to the horizontal to vertical spectral ratio (HVSR) method.

The selection of the windows with the most stationary wave forms is a crucial initial step in computing HVSR spectral ratios. This selection is used to exclude transient vibrations [6]. Subsequently, in each selected window, the Fourier spectra of each HVSR component are smoothed and merged by adopting the geometric mean.

The computation of HVSR is performed by taking the root mean square of the horizontal components of the Fourier amplitude spectra (F_NS_ and F_EW_) divided by the vertical component frequency spectrum (F_UD_) [10], as shown below.

(1) $$\frac{H}{V}=\sqrt{\frac{\left(F_{\mathrm{NS}}^{2}+F_{\mathrm{EW}}^{2}\right)}{\left(2F_{\mathrm{UD}}^{2}\right)}}$$

In the process, several parameters (window length, threshold of the short-time average/long-time average (STA/LTA) and the lengths of STA/LTA) were employed. Each selected window was then smoothed using a smoothing constant of 40 [11].

**Fig. 6:**
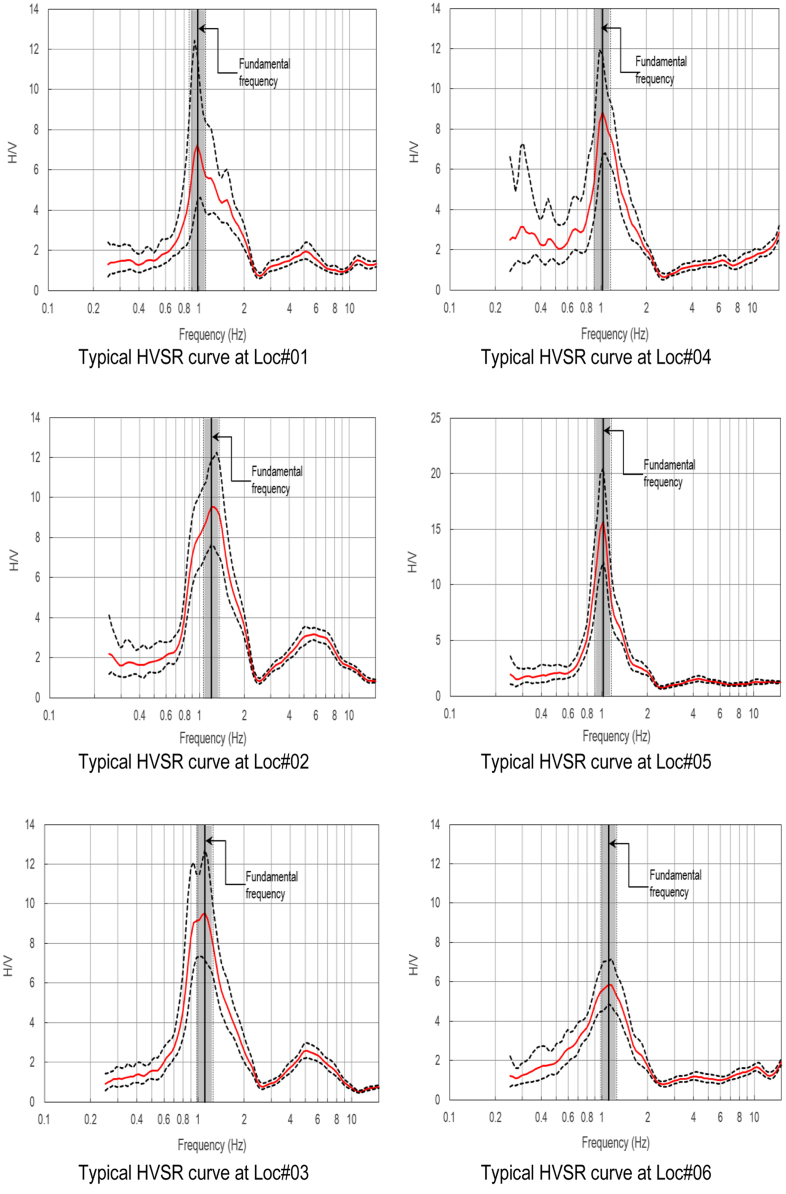
Selected HVSR curves at Locations #01 to #06.

**Fig. 7:**
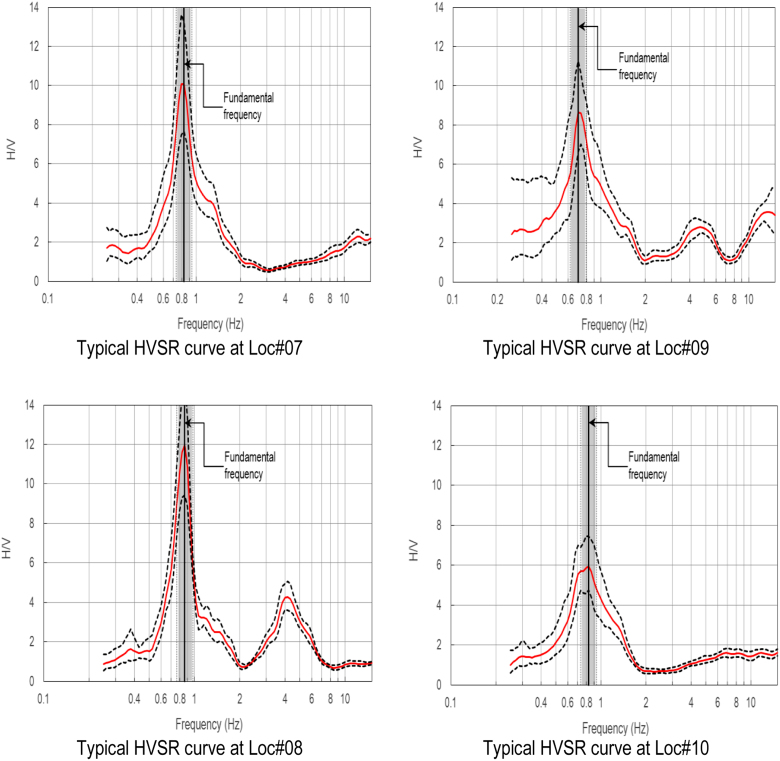
Selected HVSR curves Locations #07 to #10.

HVSR analysis was carried out using the method proposed by [11] to obtain the HVSR ellipticity curves, from which the site fundamental frequency is obtained. The results of this HVSR analysis, at all measured sites, are included in Appendix B of the Supplementary Data associated with the present paper. Prior to the HVSR analysis, as presented in this paper, several tentative window lengths (i.e. 25, 30, 35 and 40 s) were trialed to obtain as many reliable HVSR curves as possible, as suggested by [8], and the 40 s window length was found to be the optimal. Selected HVSR curves from the study areas are shown in Fig. 6, Fig. 7.

#### 2.5.3 Shear wave velocity profile

**Fig. 8:**
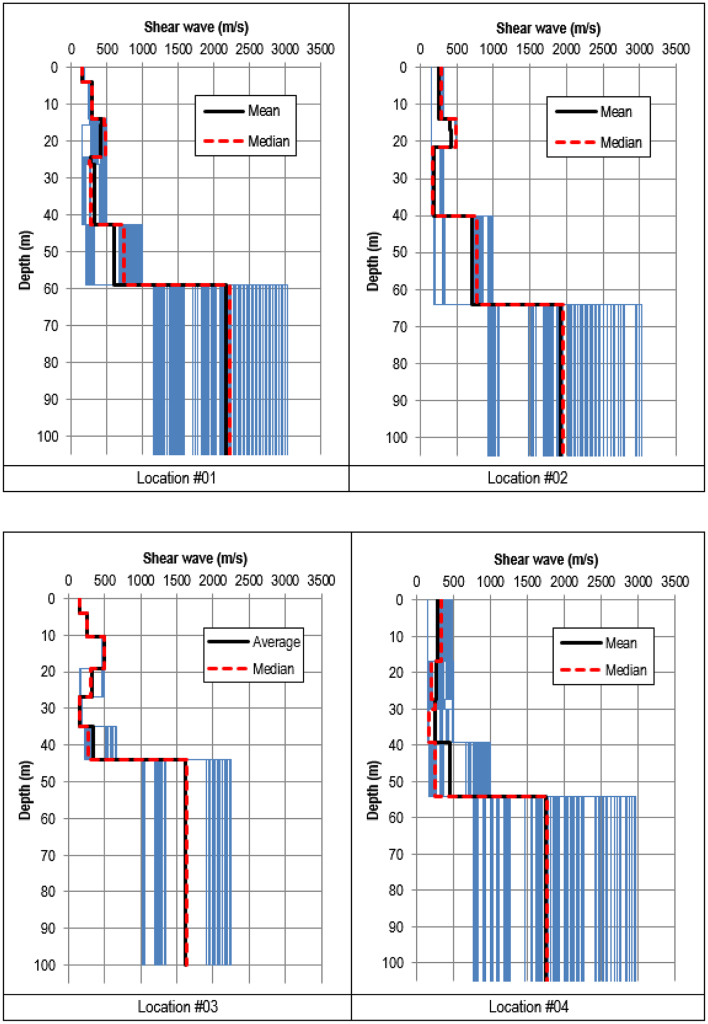
Shear wave velocity profiles inverted using the classic HVSR ellipticity constraint at Locations #01 to #04.

**Fig. 9:**
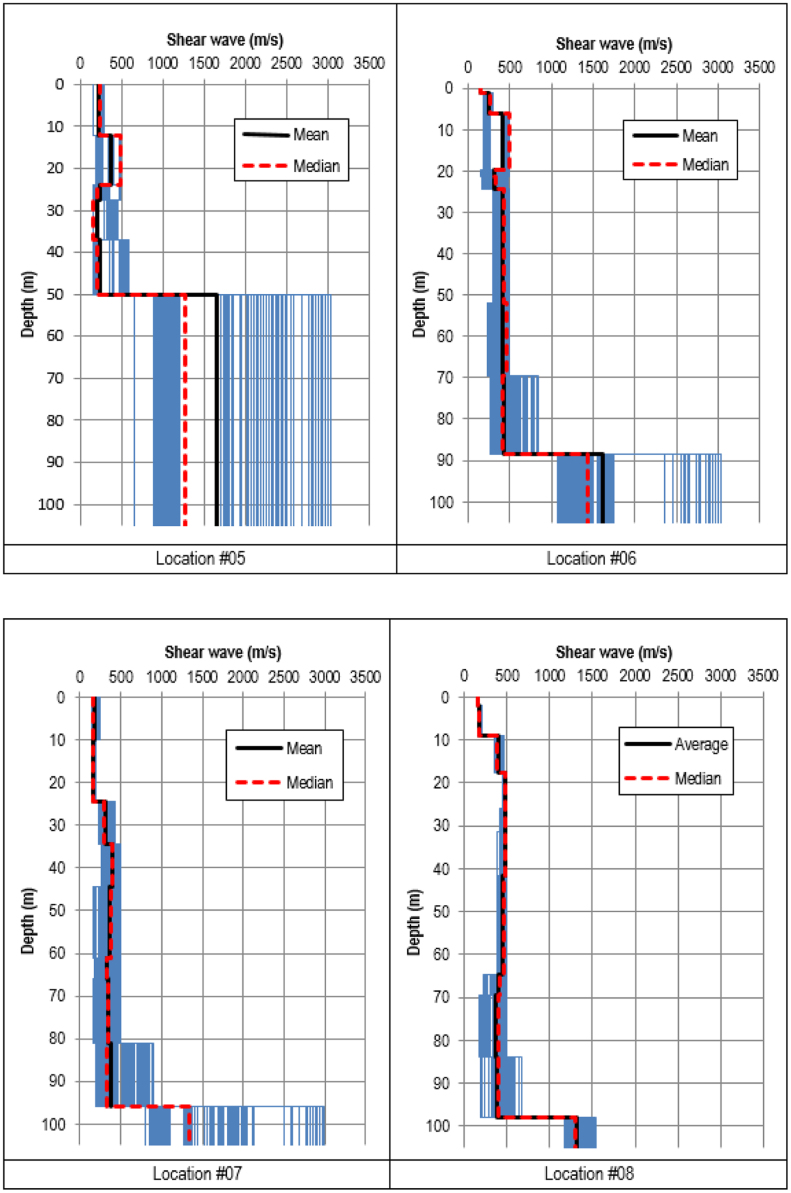
Shear wave velocity profiles inverted using the classic HVSR ellipticity constraint at Locations #05 to #08.

**Fig. 10:**
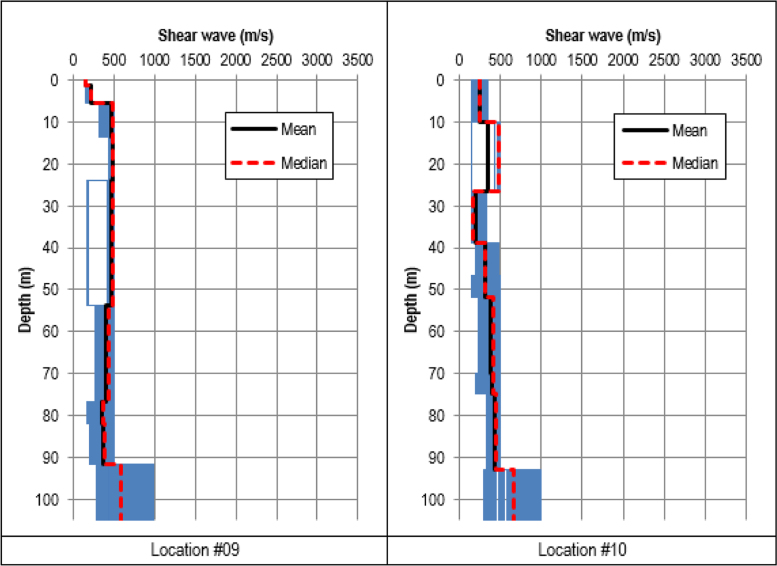
Shear wave velocity profiles inverted using the classic HVSR ellipticity constraint at Locations #09 to #10.

Shear wave velocity profiles were obtained by inverting the HVSR ellipticity curves using the process recommended by [8] and 20 best shear wave velocity models were extracted from the results of the inversion. The profiles are presented in Fig. 8, Fig. 9, Fig. 10. From the 20 best models, arithmetic mean and median values of the shear wave velocity were calculated.
