# Supplementary material for: Passive noise datasets at regolith sites
Source: Data Brief. 2018 Aug 31;20:735–47. doi: 10.1016/j.dib.2018.08.055 (PMC6129695; doi:10.1016/j.dib.2018.08.055)
Supplement: Supplementary file 2 — Supplementary material [file mmc2.docx]

**Appendix A: Industrial origin detection results at all locations**

| 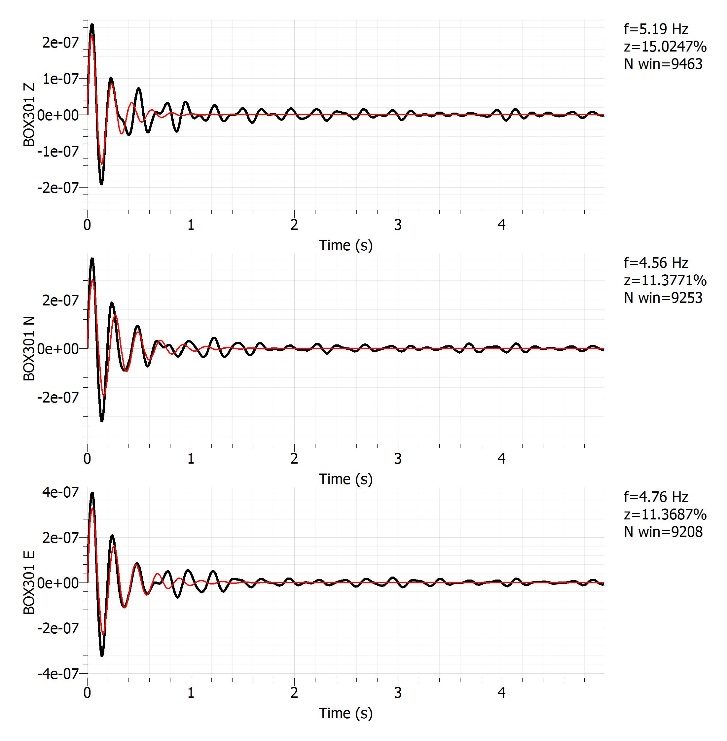 | | | 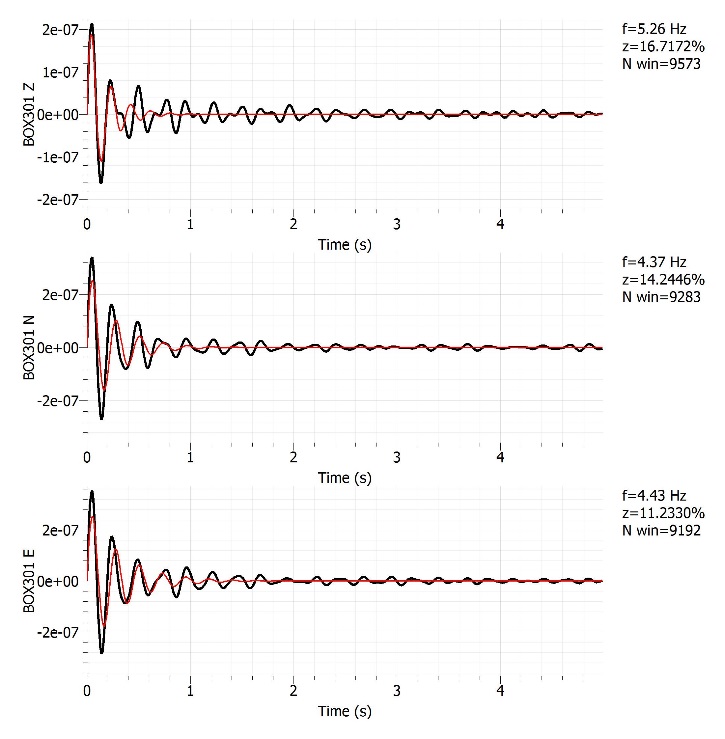 | | |
| --- | --- | --- | --- | --- | --- |
| Estimated damping | Up-Down | 15% | Estimated damping | Up-Down | 16% |
|  | North-South | 11% |  | North-South | 14% |
|  | East-West | 11% |  | East-West | 11% |
| Sustained frequency? | | No | Sustained frequency? | | No |
| Trial#01 | | | Trial#2 | | |
| 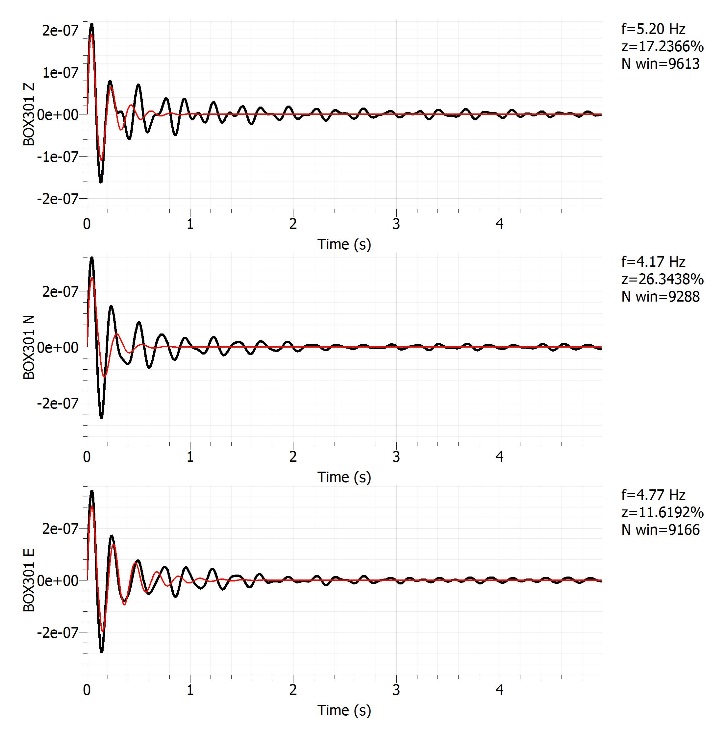 | | | 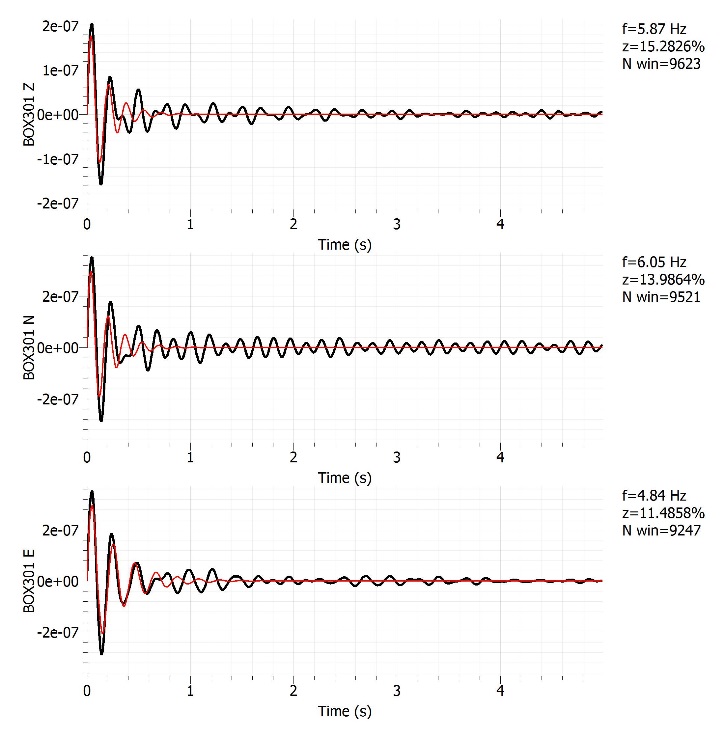 | | |
| Estimated damping | Up-Down | 17% | Estimated damping | Up-Down | 15% |
|  | North-South | 26% |  | North-South | 13% |
|  | East-West | 11% |  | East-West | 11% |
| Sustained frequency? | | No | Sustained frequency? | | No |
| Trial#03 | | | Trial#4 | | |
| **Industrial origin detection at Location #01** | | | | | |

| 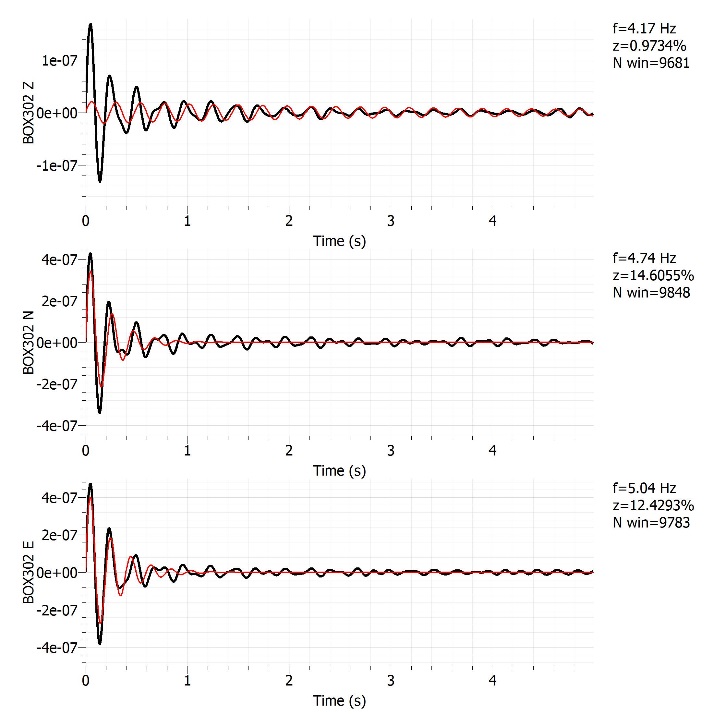 | | | 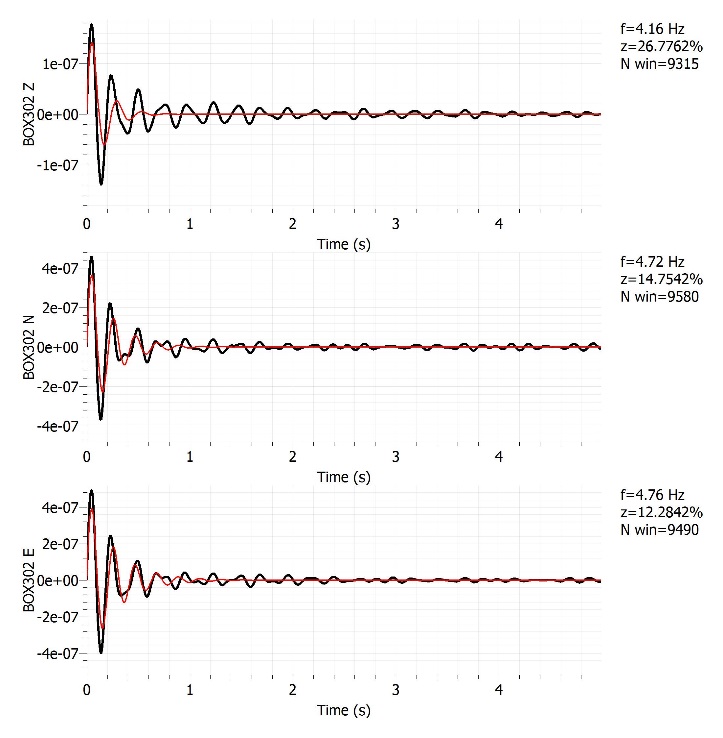 | | |
| --- | --- | --- | --- | --- | --- |
| Estimated damping | Up-Down | 0.97% | Estimated damping | Up-Down | 26% |
|  | North-South | 15% |  | North-South | 14% |
|  | East-West | 12% |  | East-West | 12% |
| Sustained frequency? | | No | Sustained frequency? | | No |
| Trial#01 | | | Trial#2 | | |
| 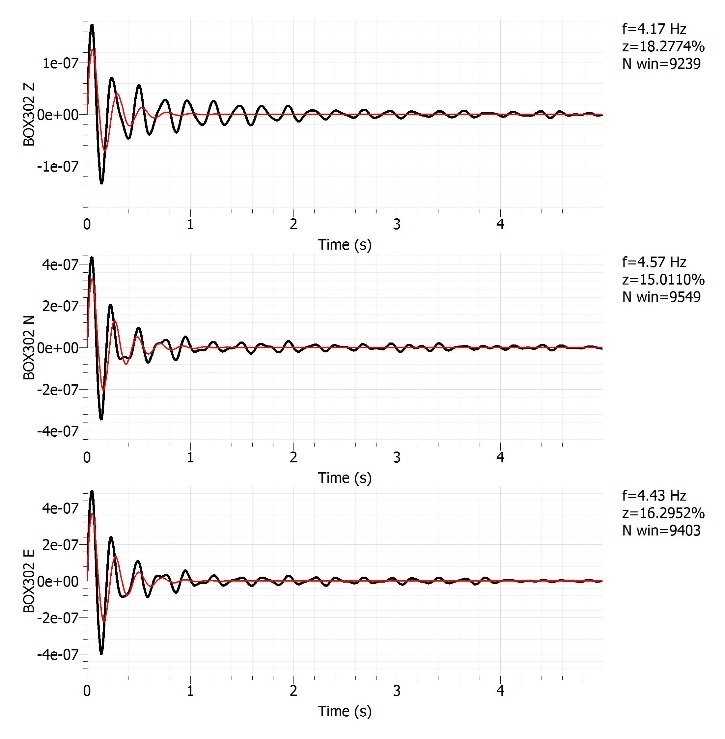 | | | 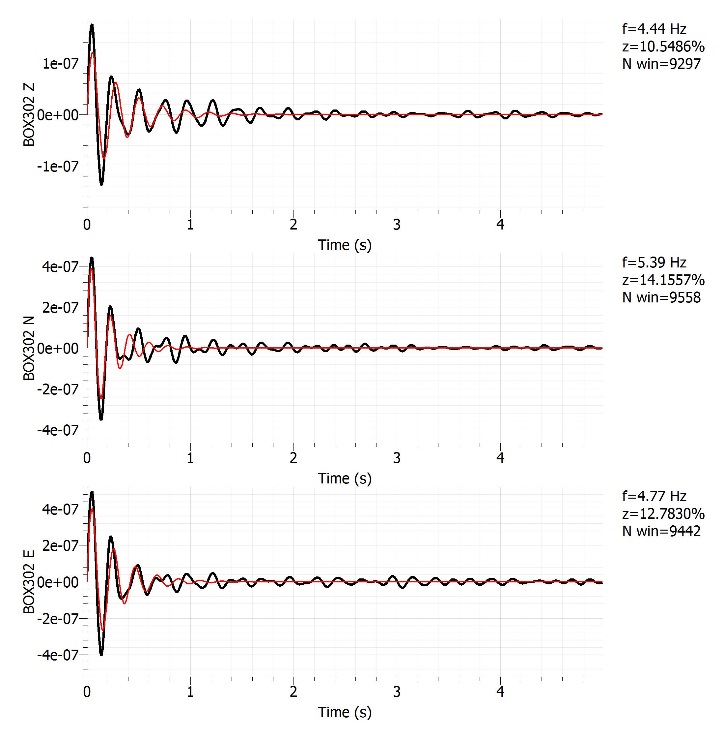 | | |
| Estimated damping | Up-Down | 18% | Estimated damping | Up-Down | 10% |
|  | North-South | 15% |  | North-South | 14% |
|  | East-West | 16% |  | East-West | 12% |
| Sustained frequency? | | No | Sustained frequency? | | No |
| Trial#03 | | | Trial#4 | | |
| **Industrial origin detection at Location #02** | | | | | |

| 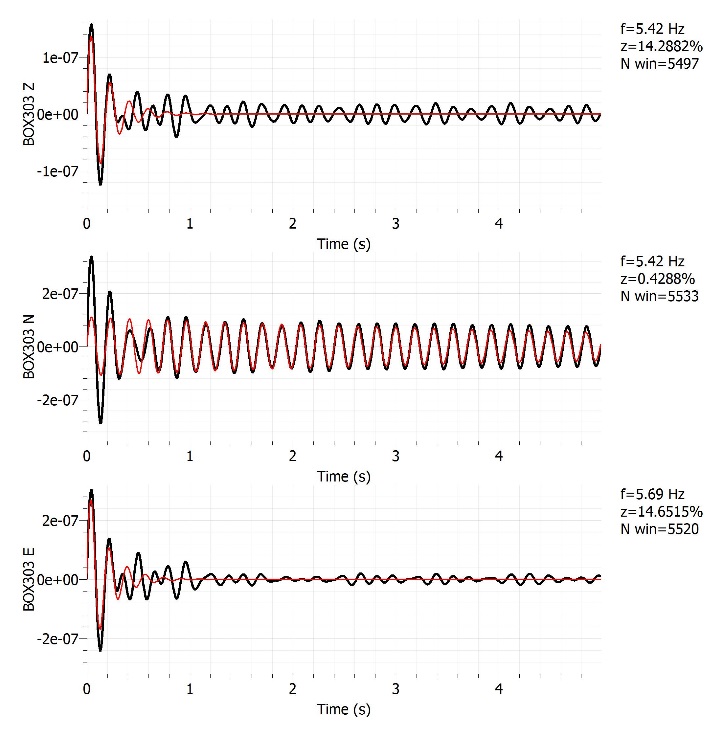 | | | 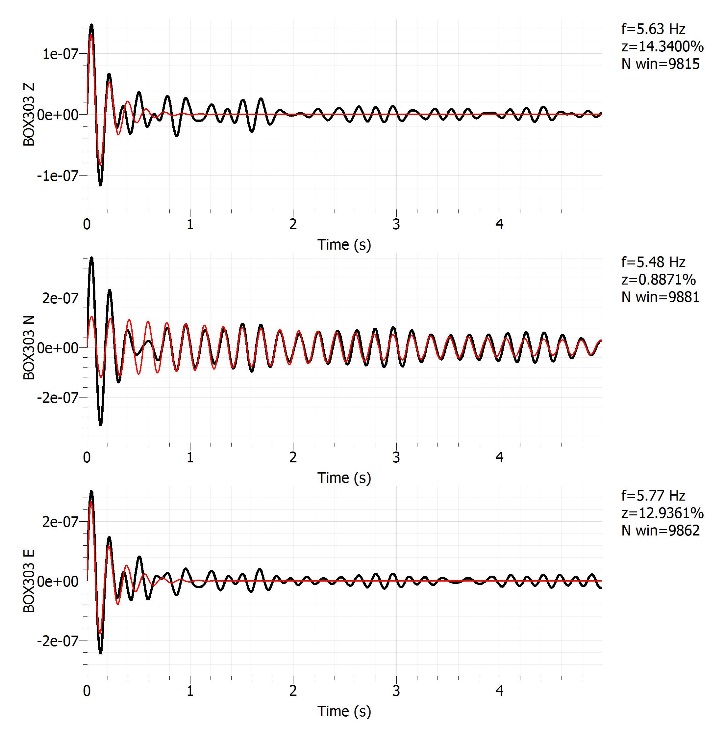 | | |
| --- | --- | --- | --- | --- | --- |
| Estimated damping | Up-Down | 14% | Estimated damping | Up-Down | 14% |
|  | North-South | 0.42% |  | North-South | 0.88% |
|  | East-West | 14% |  | East-West | 12% |
| Sustained frequency? | | Yes for North-South data only. | Sustained frequency? | | No |
| Trial#01 | | | Trial#2 | | |
| 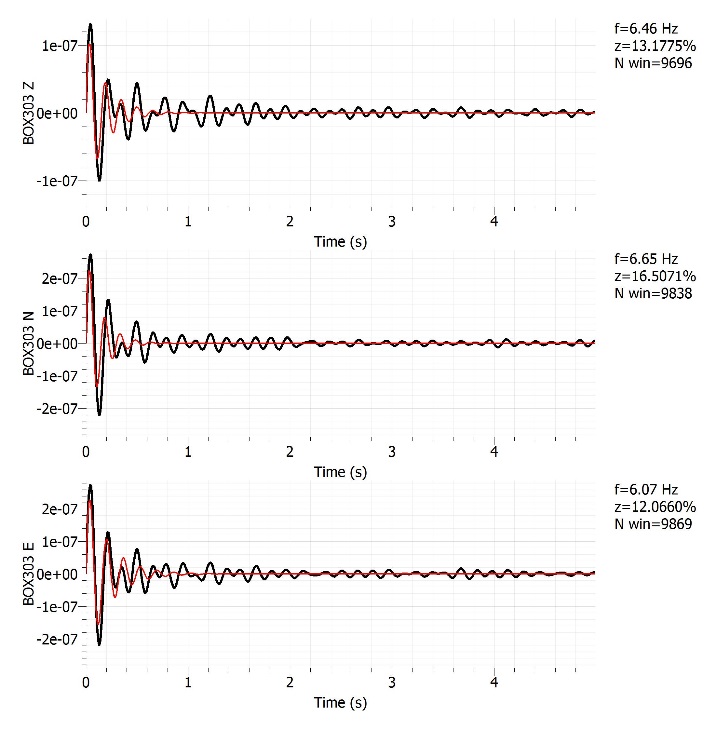 | | | 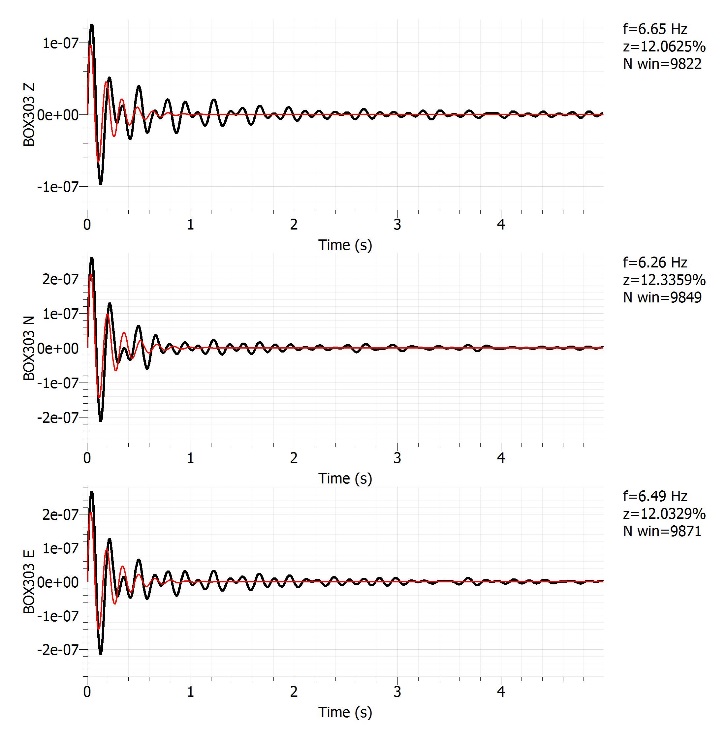 | | |
| Estimated damping | Up-Down | 13% | Estimated damping | Up-Down | 12% |
|  | North-South | 16% |  | North-South | 12% |
|  | East-West | 12% |  | East-West | 12% |
| Sustained frequency? | | No | Sustained frequency? | | No |
| Trial#03 | | | Trial#4 | | |
| **Industrial origin detection at Location #03** | | | | | |

| 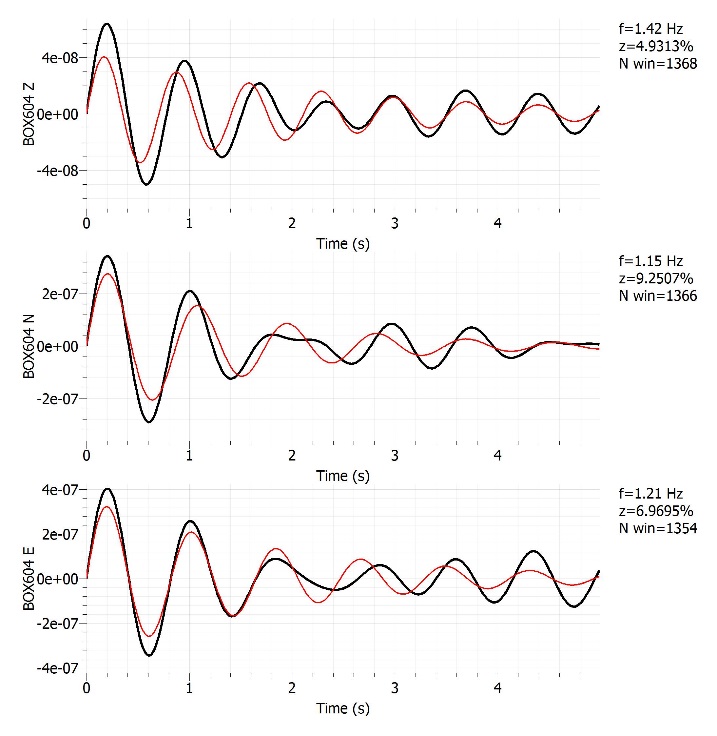 | | | 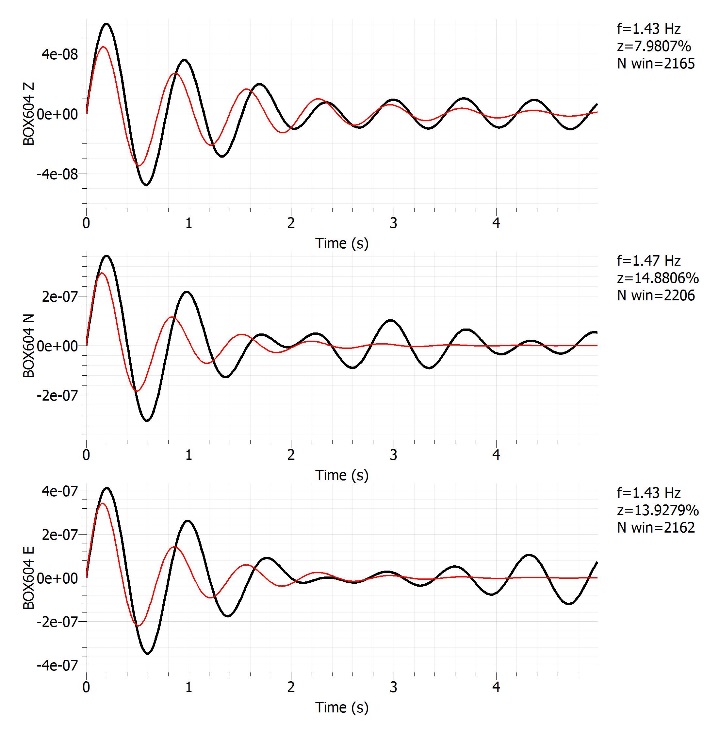 | | |
| --- | --- | --- | --- | --- | --- |
| Estimated damping | Up-Down | 4% | Estimated damping | Up-Down | 7% |
|  | North-South | 9% |  | North-South | 14% |
|  | East-West | 6% |  | East-West | 13% |
| Sustained frequency? | | No | Sustained frequency? | | No |
| Trial#01 | | | Trial#2 | | |
| 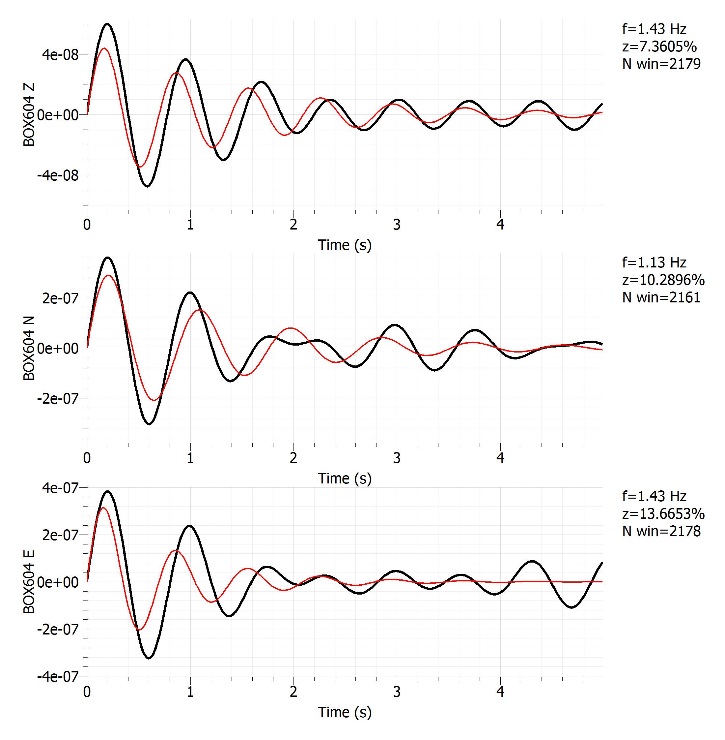 | | | 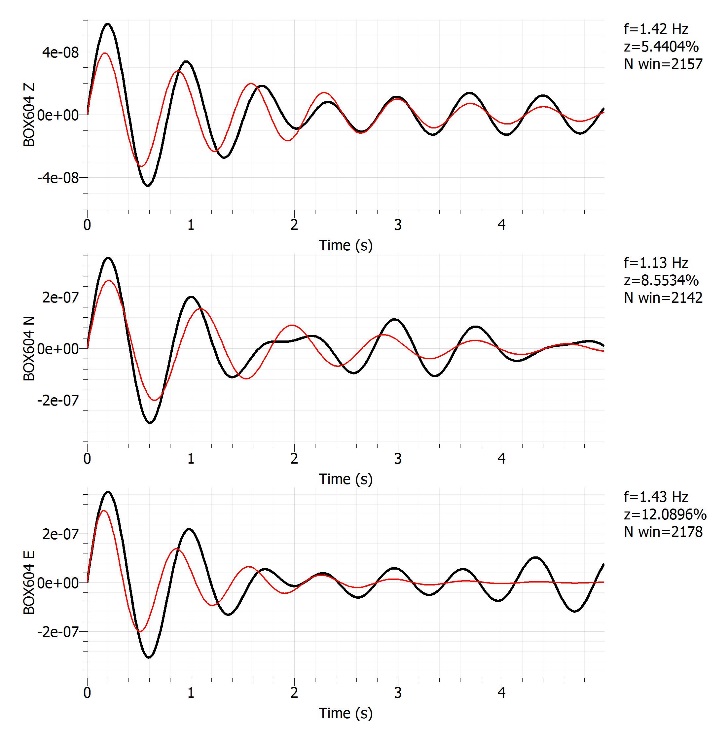 | | |
| Estimated damping | Up-Down | 7% | Estimated damping | Up-Down | 5% |
|  | North-South | 10% |  | North-South | 8% |
|  | East-West | 13% |  | East-West | 12% |
| Sustained frequency? | | No | Sustained frequency? | | No |
| Trial#03 | | | Trial#4 | | |
| **Industrial origin detection at Location #04** | | | | | |

| 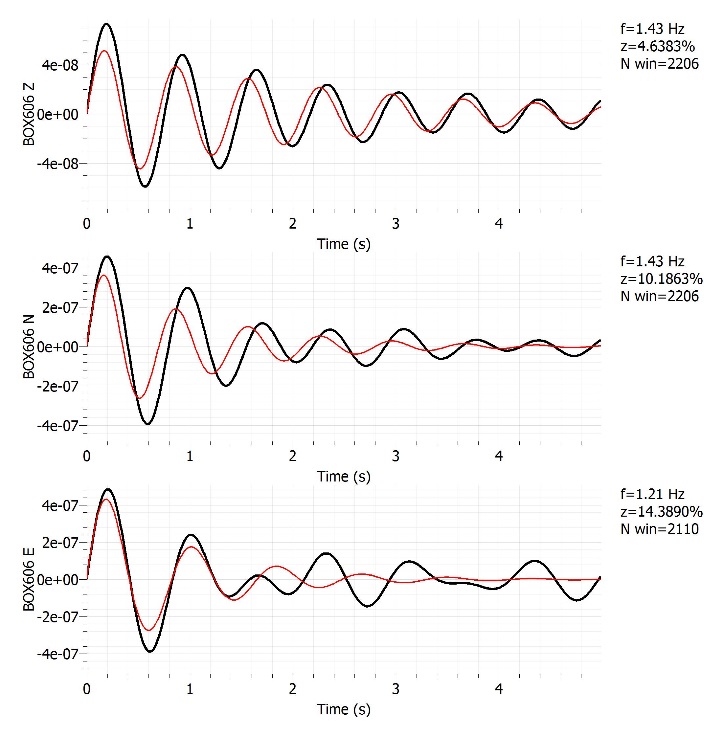 | | | 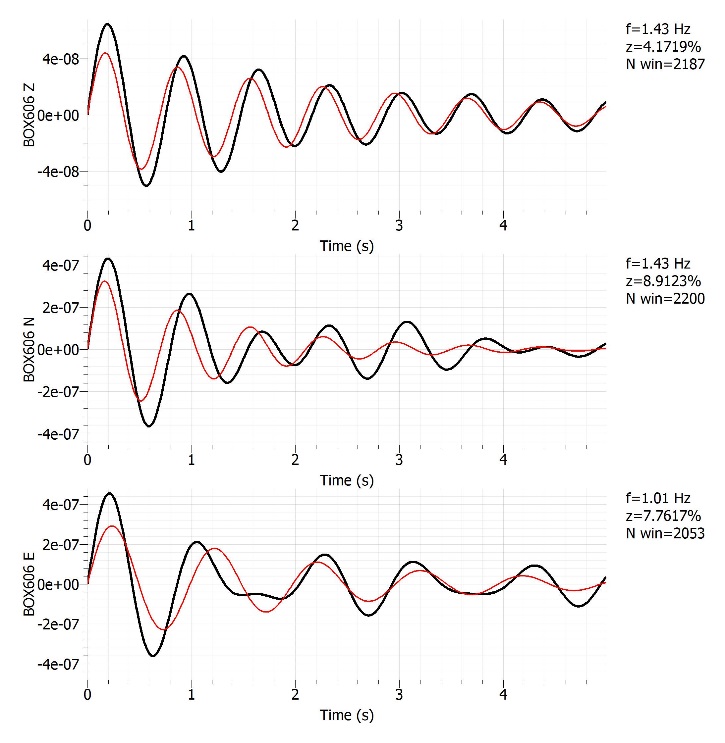 | | |
| --- | --- | --- | --- | --- | --- |
| Estimated damping | Up-Down | 4% | Estimated damping | Up-Down | 4% |
|  | North-South | 10% |  | North-South | 8% |
|  | East-West | 14% |  | East-West | 7% |
| Sustained frequency? | | No | Sustained frequency? | | No |
| Trial#01 | | | Trial#2 | | |
| 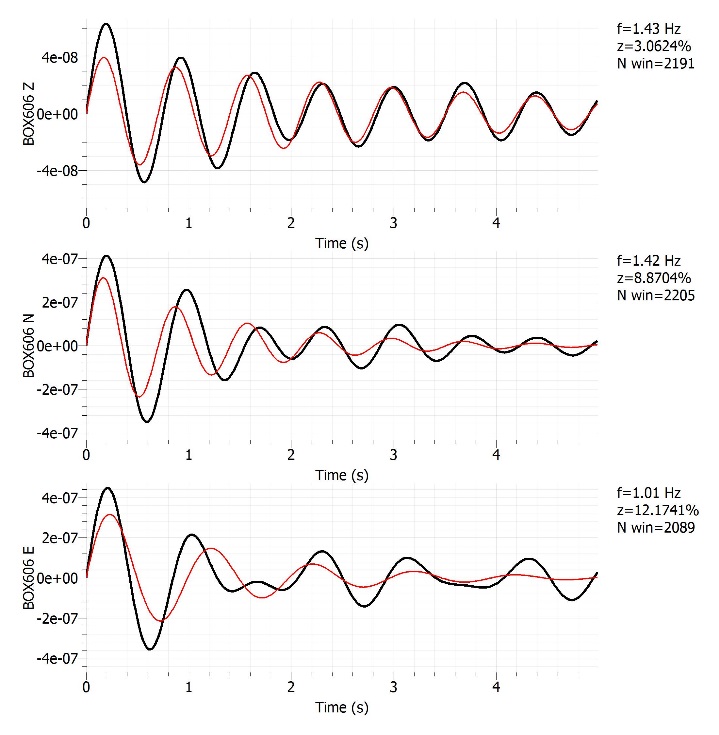 | | | 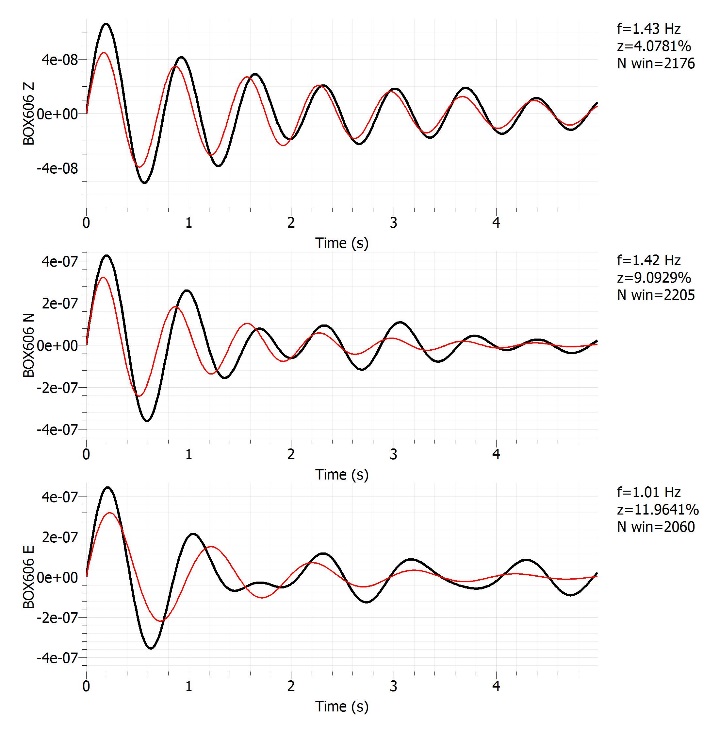 | | |
| Estimated damping | Up-Down | 3% | Estimated damping | Up-Down | 4% |
|  | North-South | 8% |  | North-South | 9% |
|  | East-West | 12% |  | East-West | 11% |
| Sustained frequency? | | No | Sustained frequency? | | No |
| Trial#03 | | | Trial#4 | | |
| **Industrial origin detection at Location #05** | | | | | |

| 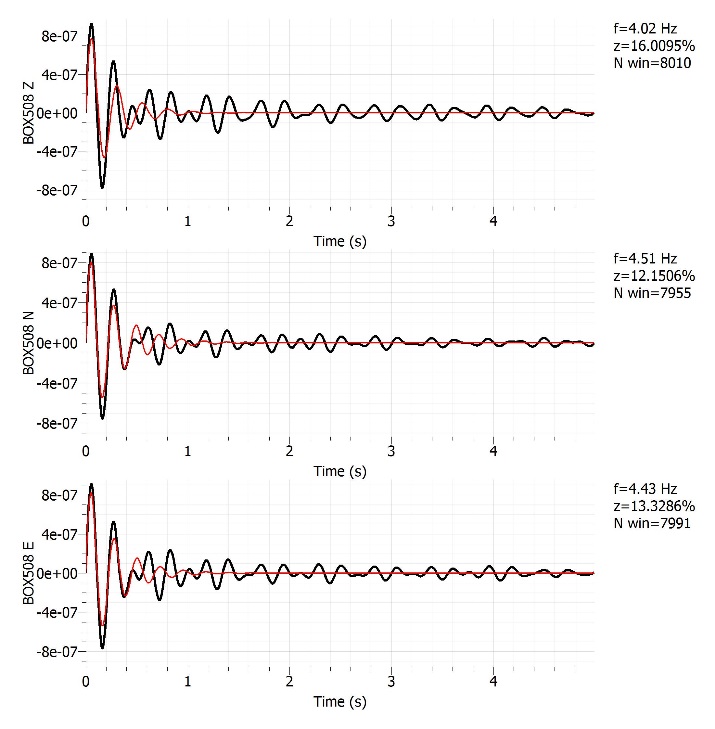 | | | 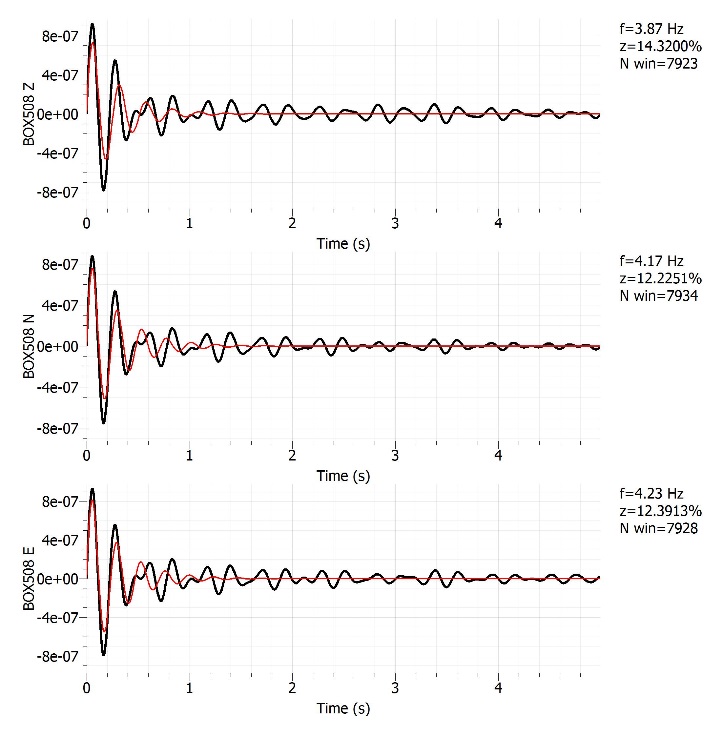 | | |
| --- | --- | --- | --- | --- | --- |
| Estimated damping | Up-Down | 16% | Estimated damping | Up-Down | 14% |
|  | North-South | 12% |  | North-South | 12% |
|  | East-West | 13% |  | East-West | 12% |
| Sustained frequency? | | No | Sustained frequency? | | No |
| Trial#01 | | | Trial#2 | | |
| 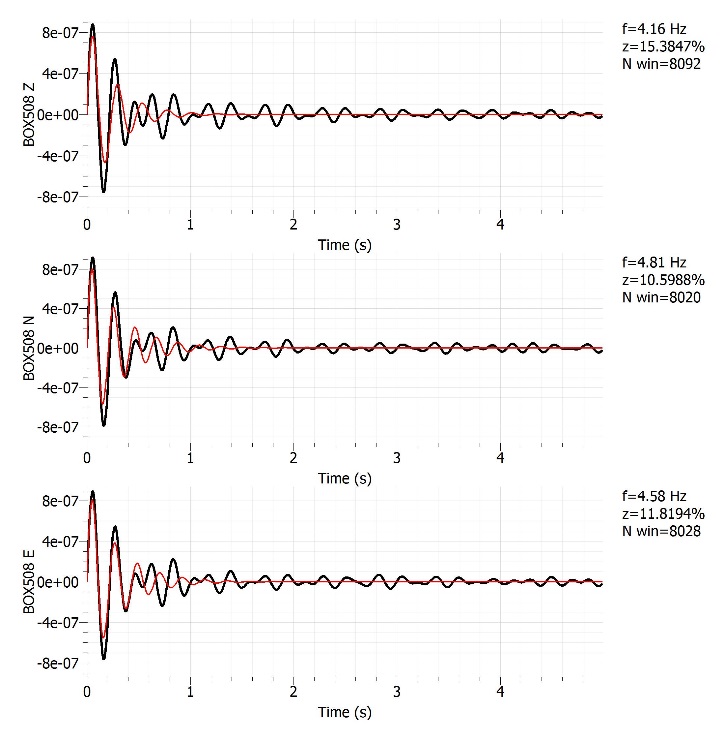 | | | 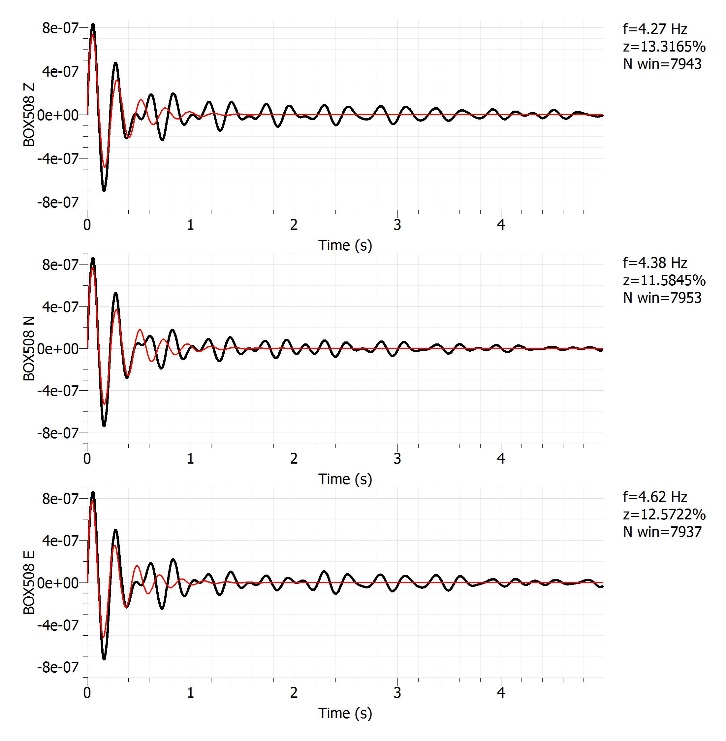 | | |
| Estimated damping | Up-Down | 15% | Estimated damping | Up-Down | 13% |
|  | North-South | 10% |  | North-South | 11% |
|  | East-West | 11% |  | East-West | 12% |
| Sustained frequency? | | No | Sustained frequency? | | No |
| Trial#03 | | | Trial#4 | | |
| **Industrial origin detection at Location #06** | | | | | |

| 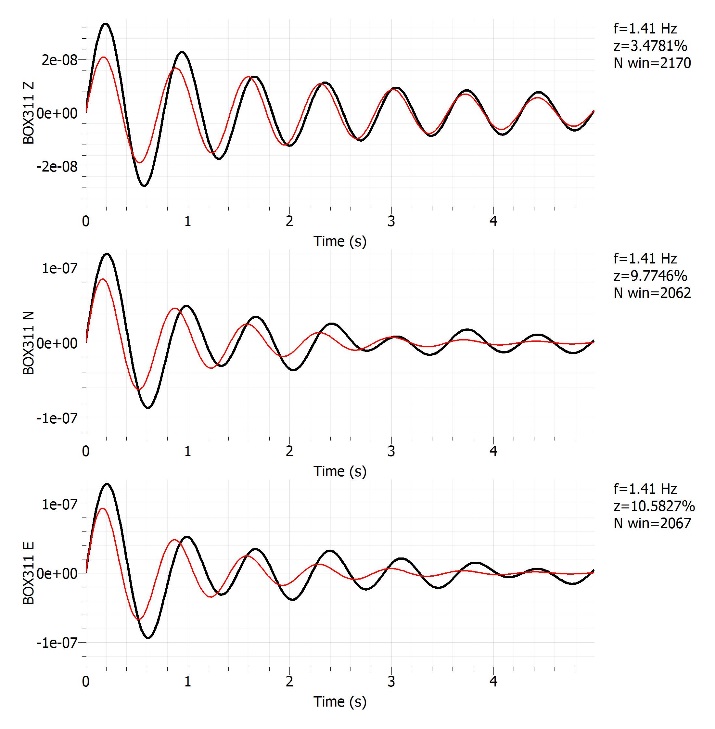 | | | 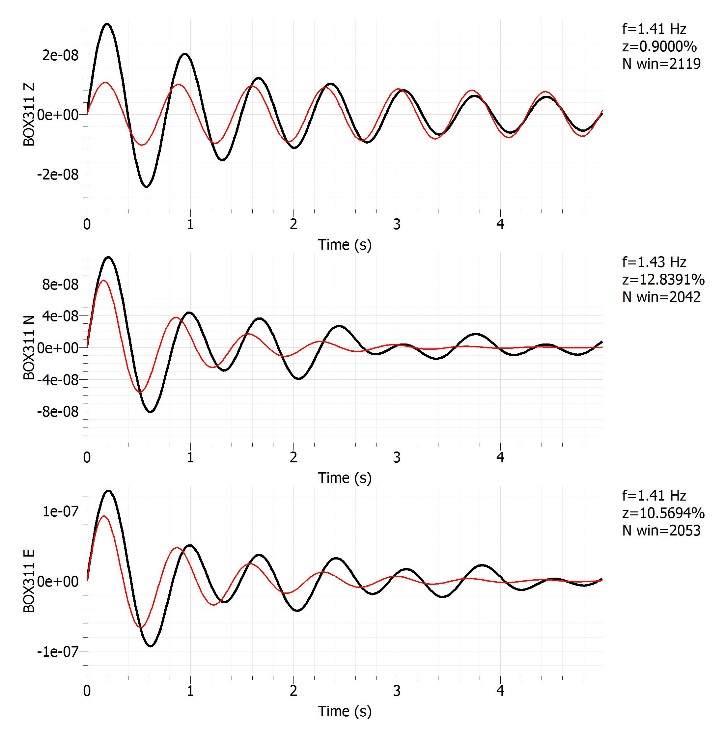 | | |
| --- | --- | --- | --- | --- | --- |
| Estimated damping | Up-Down | 3% | Estimated damping | Up-Down | 0.90% |
|  | North-South | 9% |  | North-South | 12% |
|  | East-West | 10% |  | East-West | 10% |
| Sustained frequency? | | No | Sustained frequency? | | No |
| Trial#01 | | | Trial#2 | | |
| 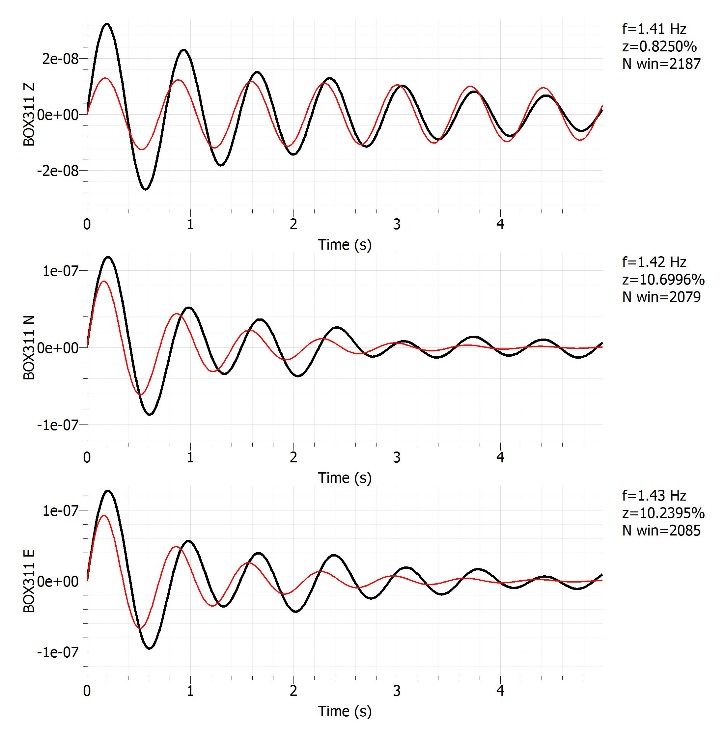 | | | 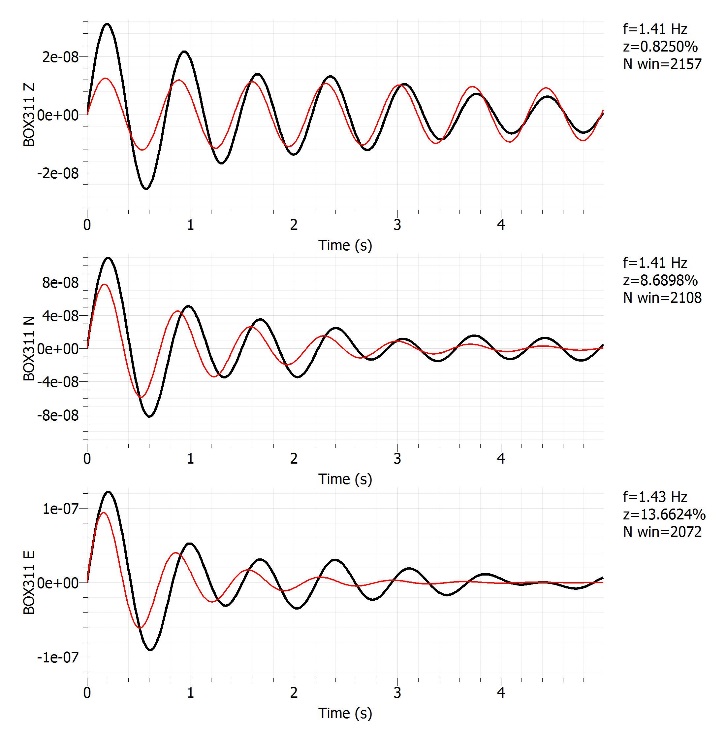 | | |
| Estimated damping | Up-Down | 0.82% | Estimated damping | Up-Down | 0.82% |
|  | North-South | 10% |  | North-South | 8% |
|  | East-West | 10% |  | East-West | 13% |
| Sustained frequency? | | No | Sustained frequency? | | No |
| Trial#03 | | | Trial#4 | | |
| **Industrial origin detection at Location #07** | | | | | |

| 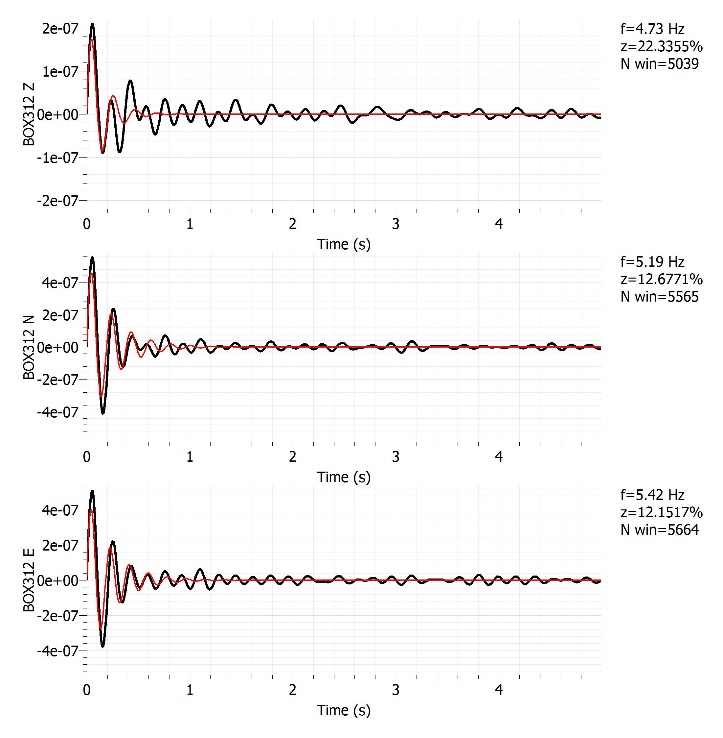 | | | 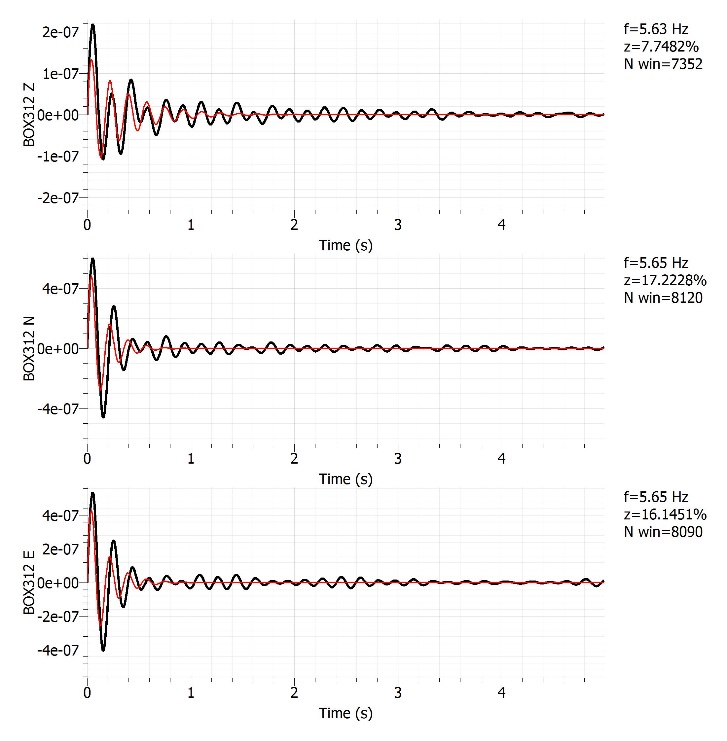 | | |
| --- | --- | --- | --- | --- | --- |
| Estimated damping | Up-Down | 22% | Estimated damping | Up-Down | 7% |
|  | North-South | 12% |  | North-South | 17% |
|  | East-West | 12% |  | East-West | 16% |
| Sustained frequency? | | No | Sustained frequency? | | No |
| Trial#01 | | | Trial#2 | | |
| 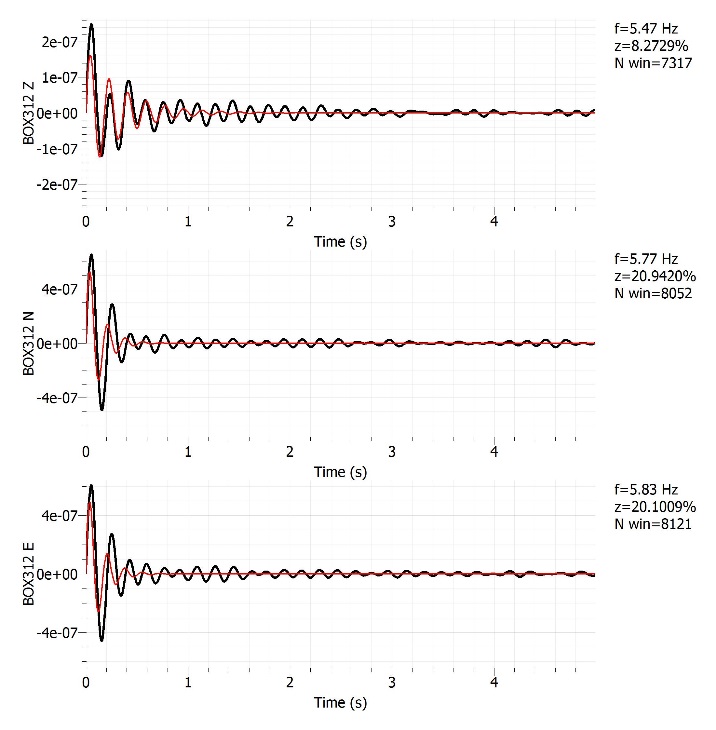 | | | 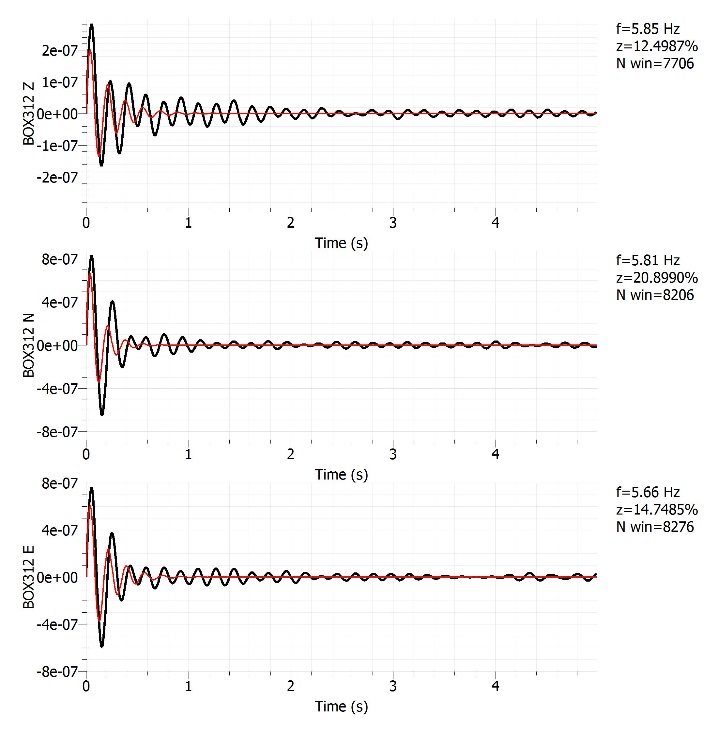 | | |
| Estimated damping | Up-Down | 8% | Estimated damping | Up-Down | 12% |
|  | North-South | 20% |  | North-South | 20% |
|  | East-West | 20% |  | East-West | 14% |
| Sustained frequency? | | No | Sustained frequency? | | No |
| Trial#03 | | | Trial#4 | | |
| **Industrial origin detection at Location #08** | | | | | |

| 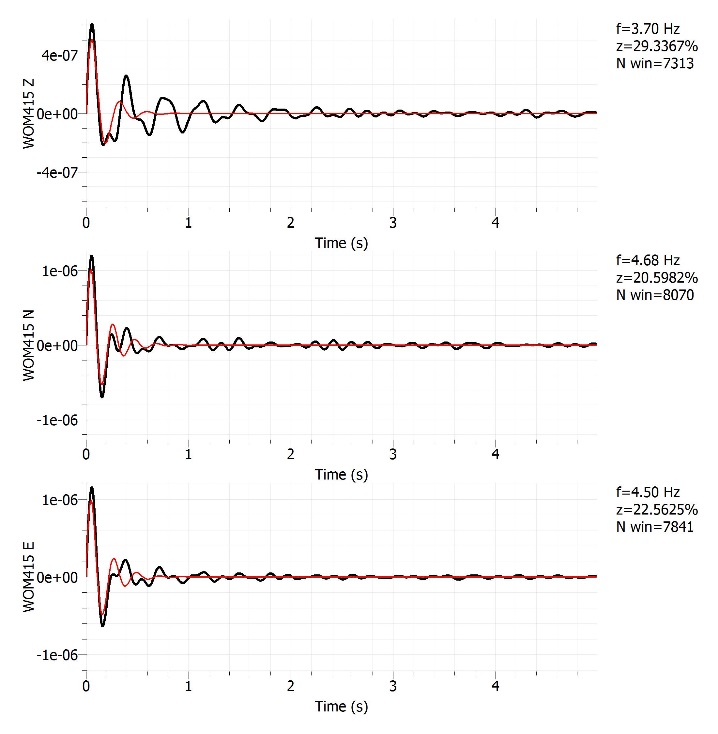 | | | 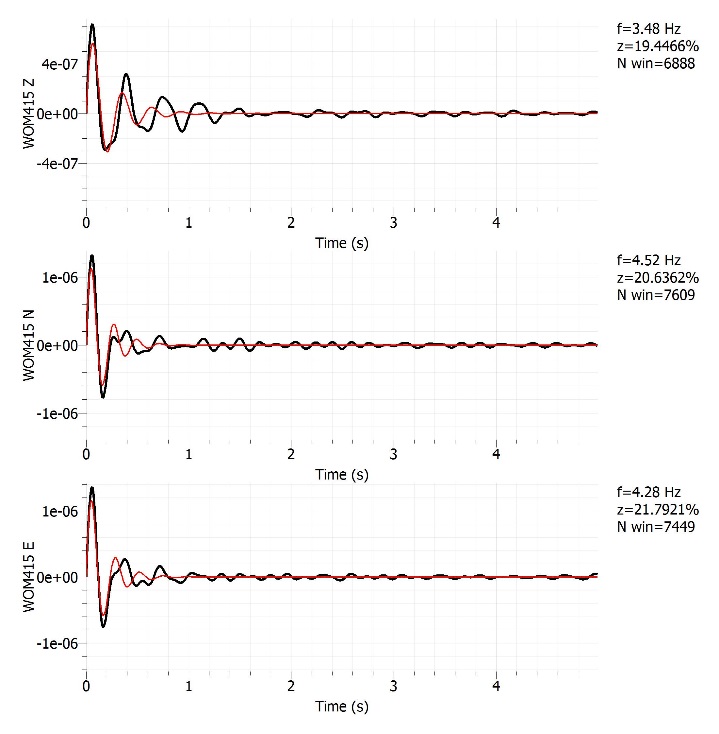 | | |
| --- | --- | --- | --- | --- | --- |
| Estimated damping | Up-Down | 29% | Estimated damping | Up-Down | 19% |
|  | North-South | 20% |  | North-South | 20% |
|  | East-West | 22% |  | East-West | 21% |
| Sustained frequency? | | No | Sustained frequency? | | No |
| Trial#01 | | | Trial#2 | | |
| 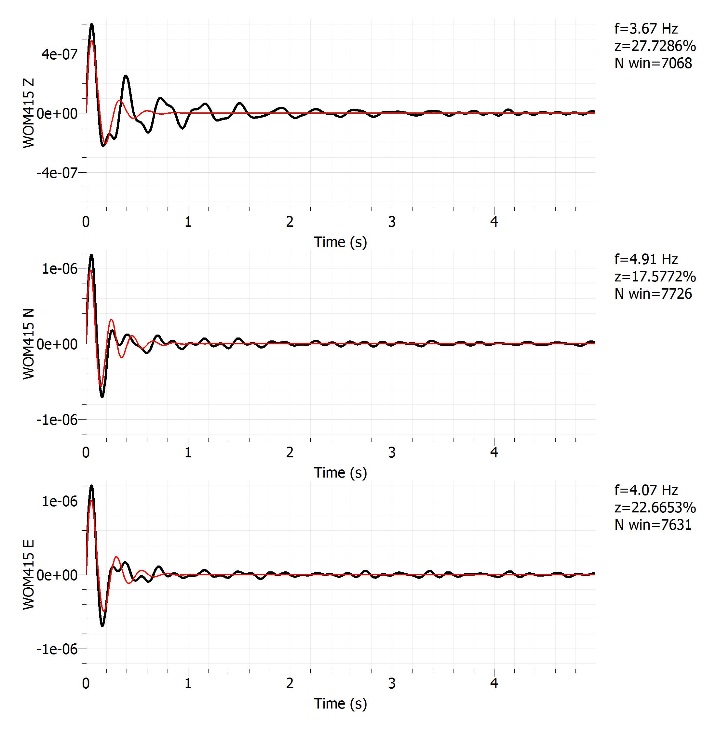 | | | 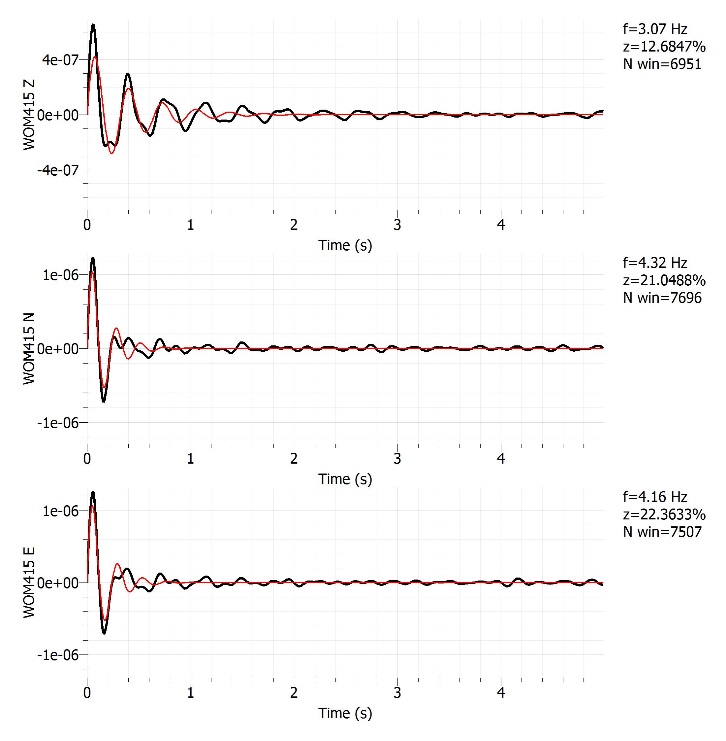 | | |
| Estimated damping | Up-Down | 27% | Estimated damping | Up-Down | 12% |
|  | North-South | 17% |  | North-South | 21% |
|  | East-West | 22% |  | East-West | 22% |
| Sustained frequency? | | No | Sustained frequency? | | No |
| Trial#03 | | | Trial#4 | | |
| **Industrial origin detection at Location #09** | | | | | |

| 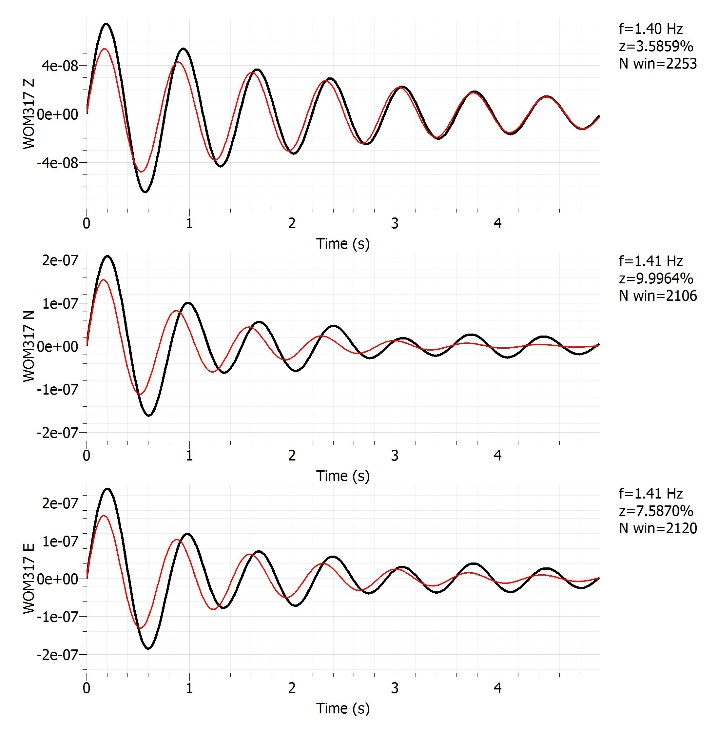 | | | 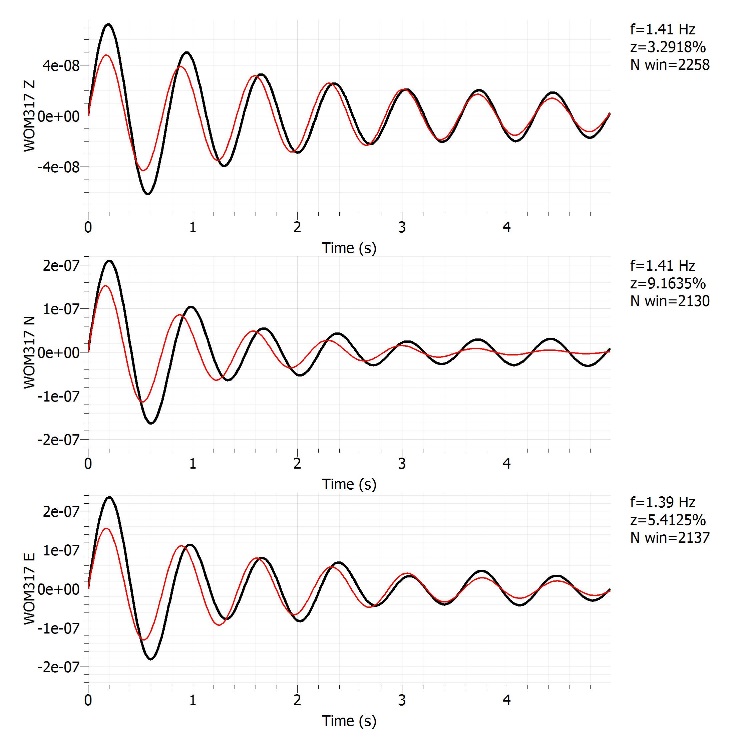 | | |
| --- | --- | --- | --- | --- | --- |
| Estimated damping | Up-Down | 3% | Estimated damping | Up-Down | 3% |
|  | North-South | 9% |  | North-South | 9% |
|  | East-West | 7% |  | East-West | 5% |
| Sustained frequency? | | No | Sustained frequency? | | No |
| Trial#01 | | | Trial#2 | | |
| 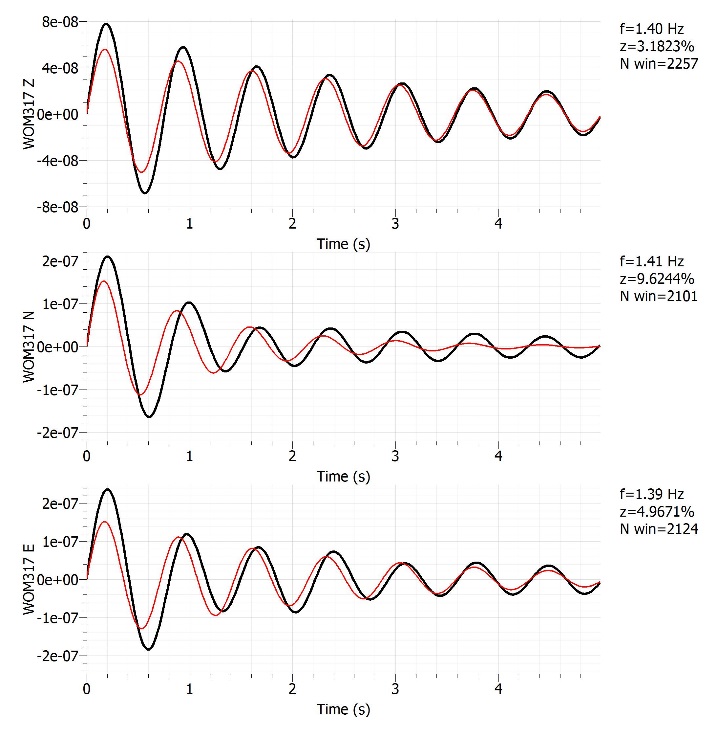 | | | 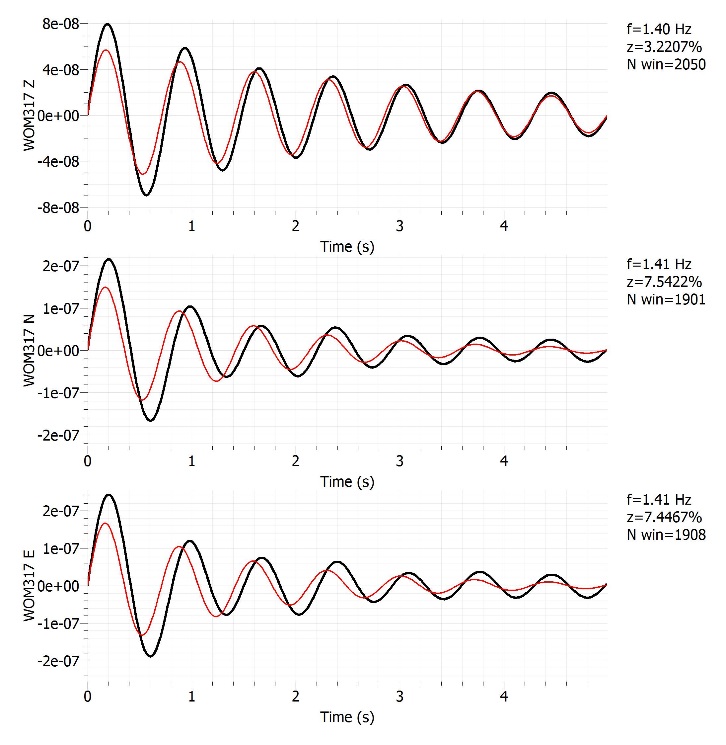 | | |
| Estimated damping | Up-Down | 3% | Estimated damping | Up-Down | 3% |
|  | North-South | 9% |  | North-South | 7% |
|  | East-West | 4% |  | East-West | 7% |
| Sustained frequency? | | No | Sustained frequency? | | No |
| Trial#03 | | | Trial#4 | | |
| **Industrial origin detection at Location #10** | | | | | |
