# Supplementary material for: Passive noise datasets at regolith sites
Source: Data Brief. 2018 Aug 31;20:735–47. doi: 10.1016/j.dib.2018.08.055 (PMC6129695; doi:10.1016/j.dib.2018.08.055)
Supplement: Supplementary file 3 — Supplementary material [file mmc3.docx]

**Appendix B: HVSR analysis curves of all trials**

| 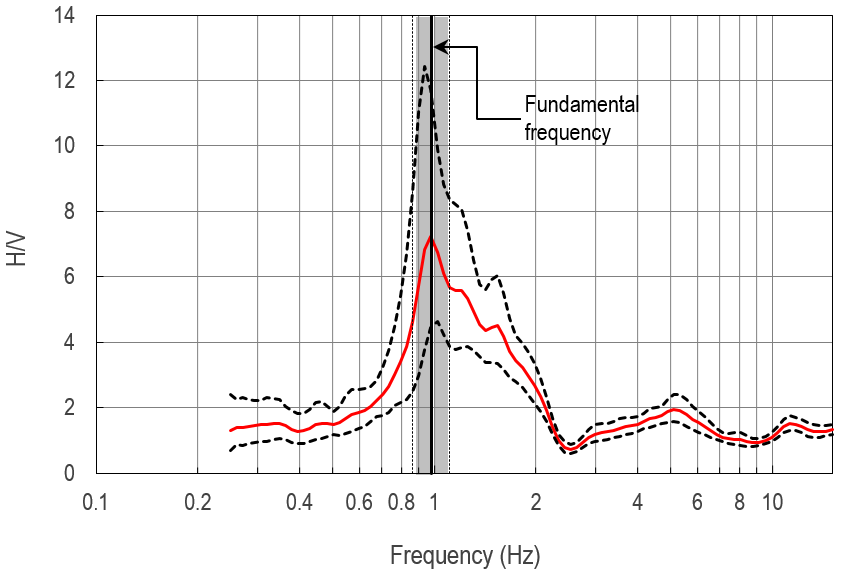 | | 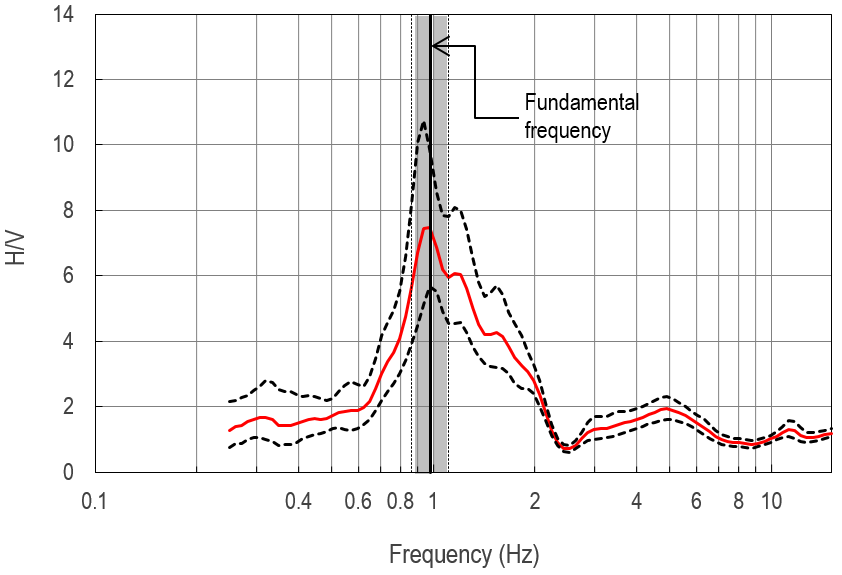 | |
| --- | --- | --- | --- |
| Fundamental frequency (Hz) | 0.970 | Fundamental frequency (Hz) | 0.979 |
| **Location 01 Instrument 01 Trial#01** | | **Location 01 Instrument 01 Trial#02** | |
| 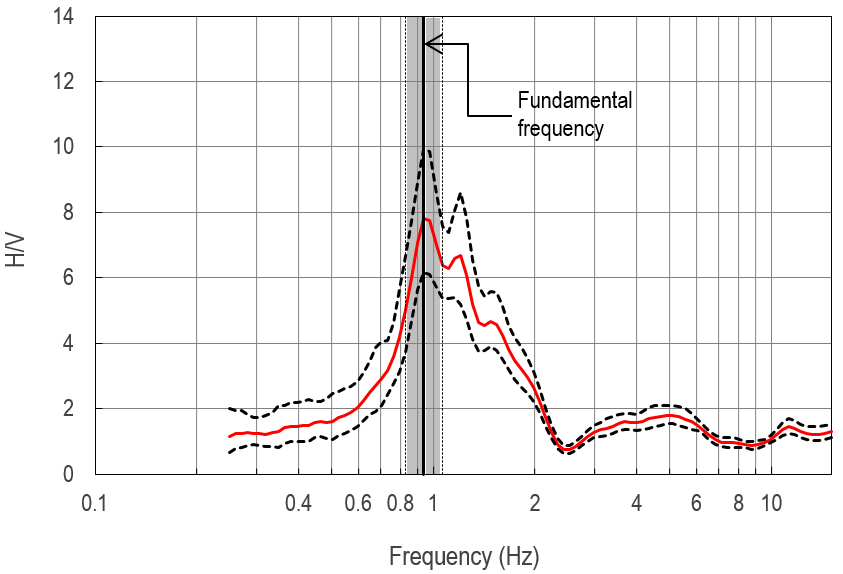 | | 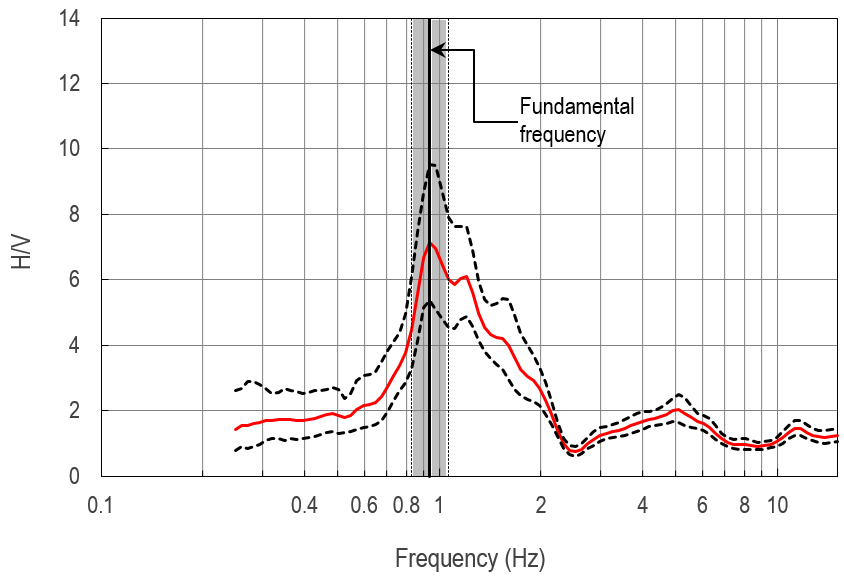 | |
| Fundamental frequency (Hz) | 0.939 | Fundamental frequency (Hz) | 0.939 |
| **Location 01 Instrument 01 Trial#03** | | **Location 01 Instrument 01 Trial#04** | |
| 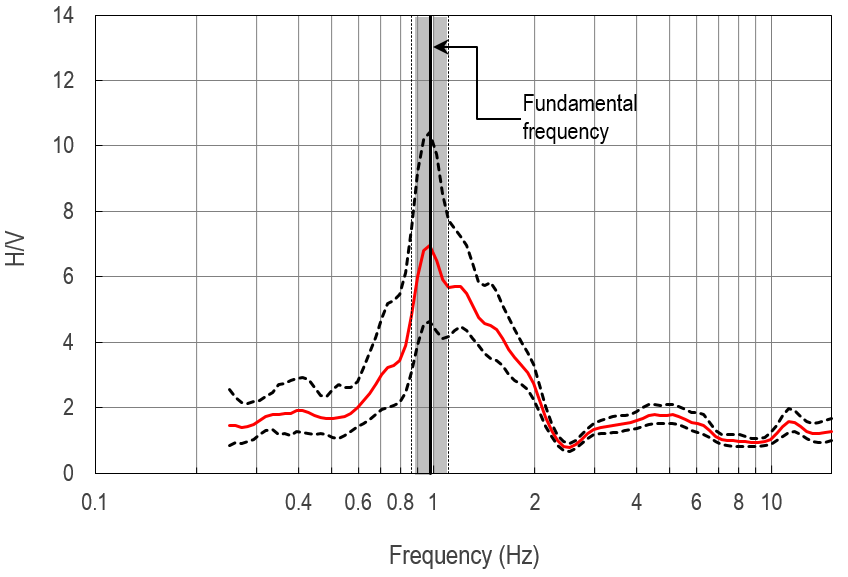 | | 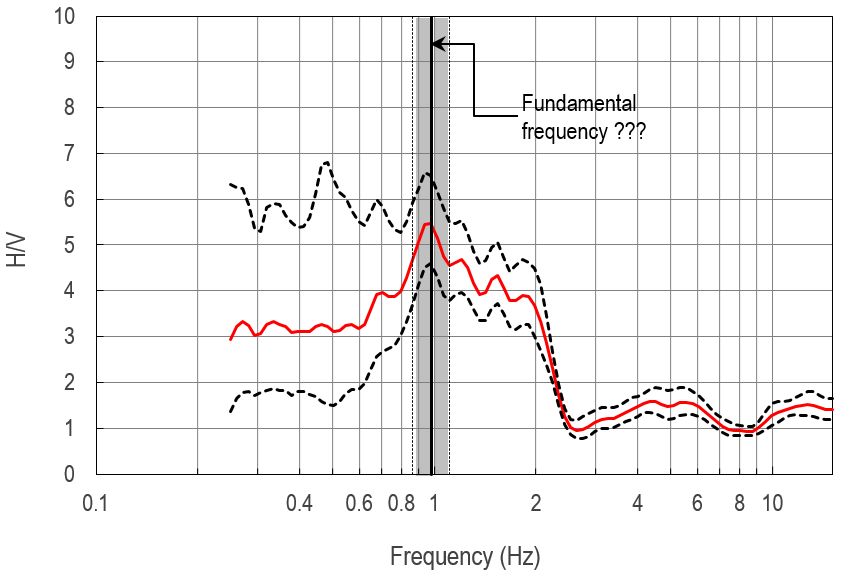 | |
| Fundamental frequency (Hz) | 0.979 | Fundamental frequency (Hz) | 0.979??? |
| **Location 01 Instrument 01 Trial#05** | | **Location 01 Instrument 02 Trial#01** | |

| 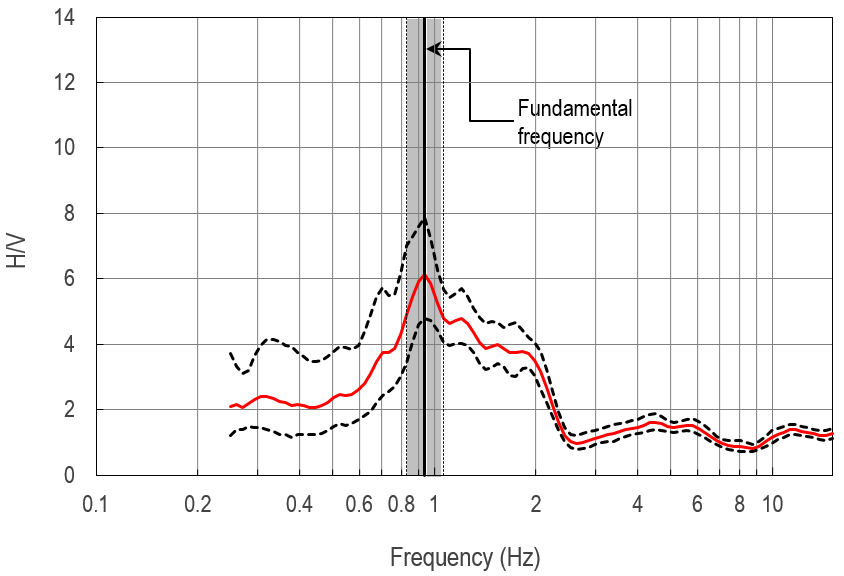 | | 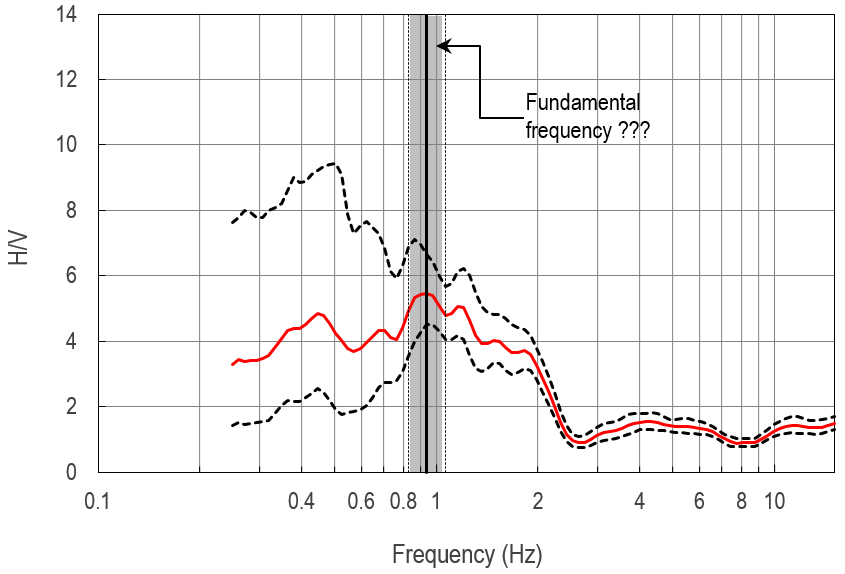 | |
| --- | --- | --- | --- |
| Fundamental frequency (Hz) | 0.939 | Fundamental frequency (Hz) | 0.939??? |
| **Location 01 Instrument 02 Trial#02** | | **Location 01 Instrument 02 Trial#03** | |
| 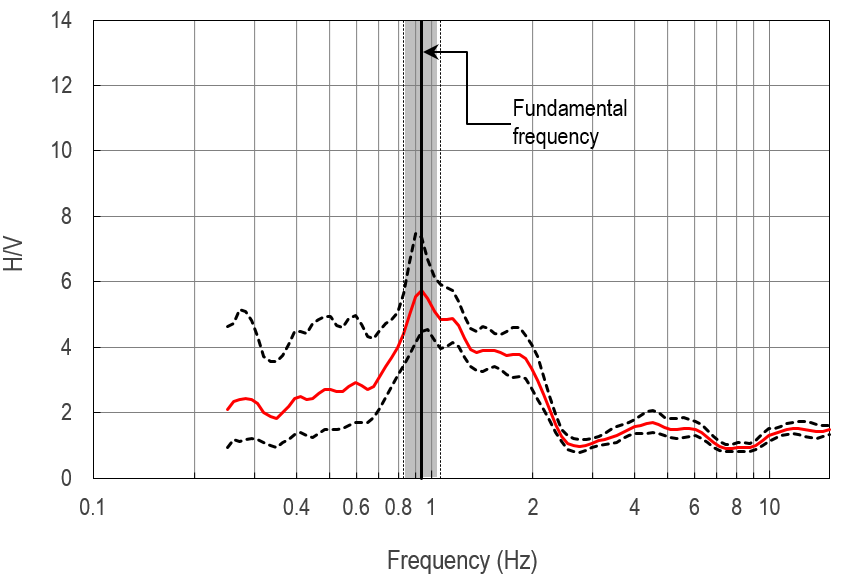 | | 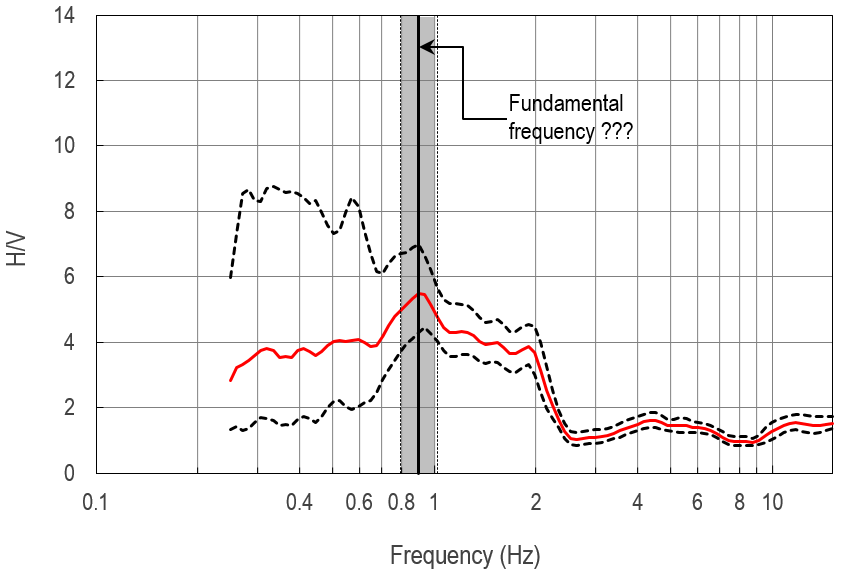 | |
| Fundamental frequency (Hz) | 0.939 | Fundamental frequency (Hz) | 0.901??? |
| **Location 01 Instrument 02 Trial#04** | | **Location 01 Instrument 02 Trial#05** | |
| 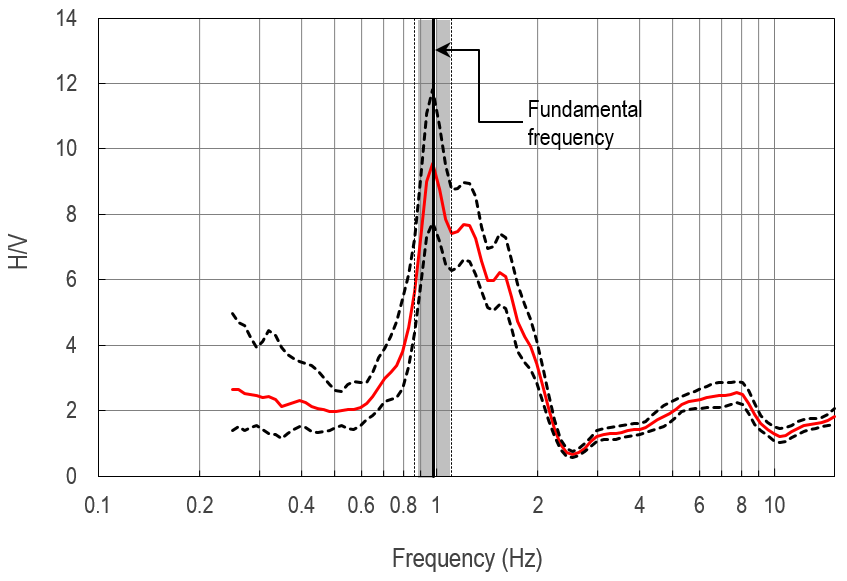 | | 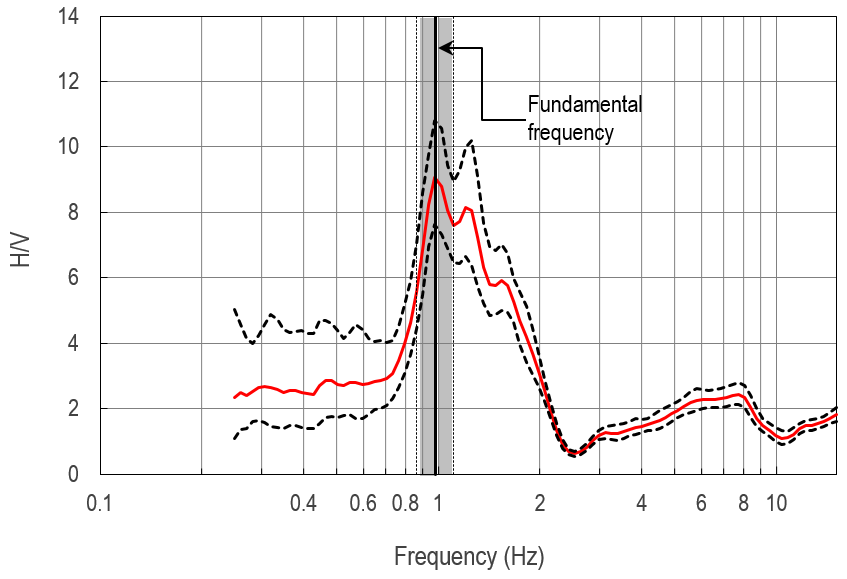 | |
| Fundamental frequency (Hz) | 0.979 | Fundamental frequency (Hz) | 0.979 |
| **Location 01 Instrument 03 Trial#01** | | **Location 01 Instrument 03 Trial#02** | |

| 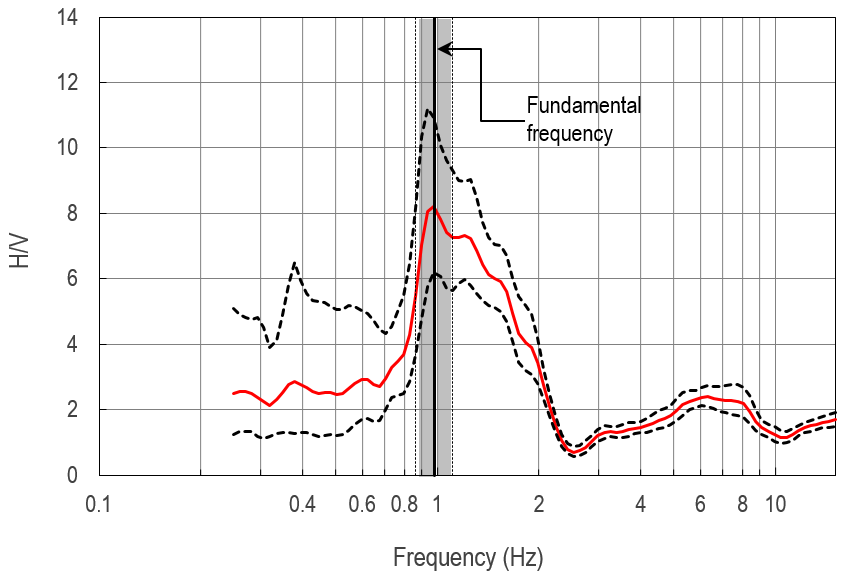 | | 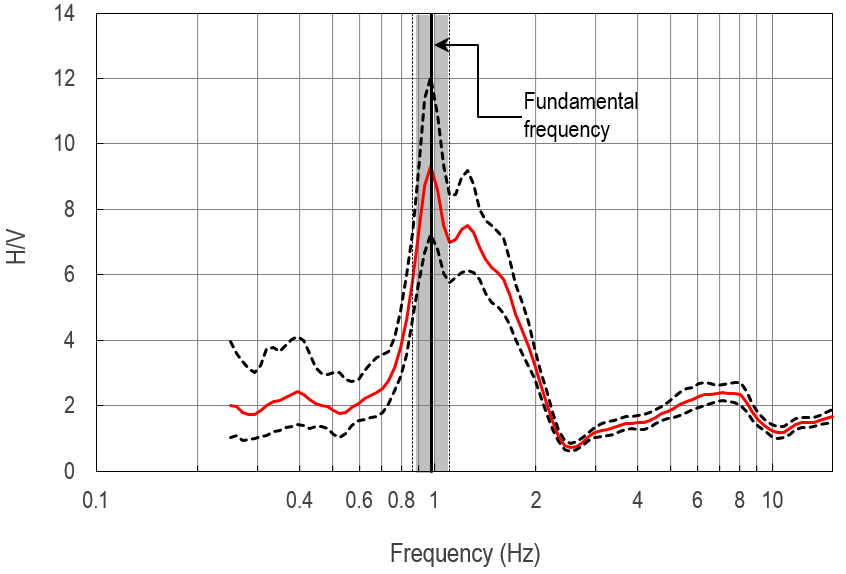 | |
| --- | --- | --- | --- |
| Fundamental frequency (Hz) | 0.979 | Fundamental frequency (Hz) | 0.979 |
| **Location 01 Instrument 03 Trial#03** | | **Location 01 Instrument 03 Trial#04** | |
| 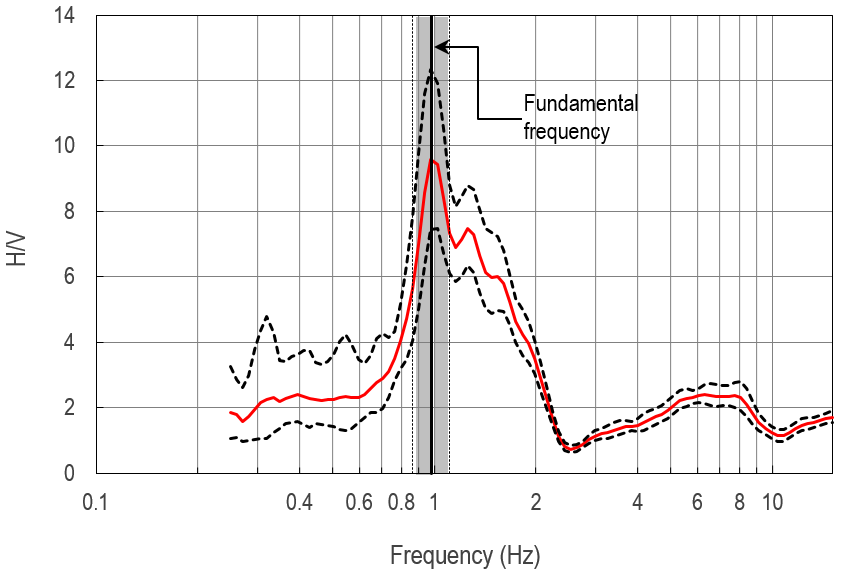 | | 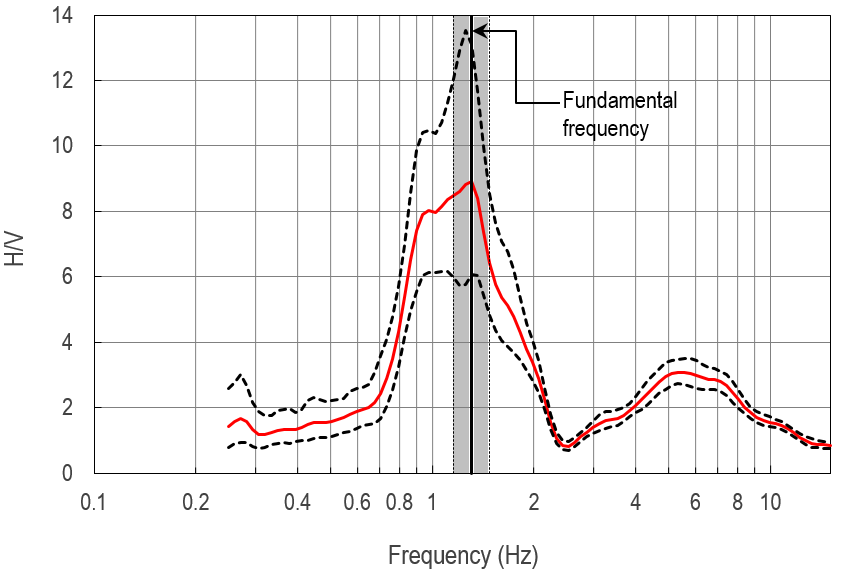 | |
| Fundamental frequency (Hz) | 0.979 | Fundamental frequency (Hz) | 1.307 |
| **Location 01 Instrument 03 Trial#05** | | **Location 02 Instrument 01 Trial#01** | |
| 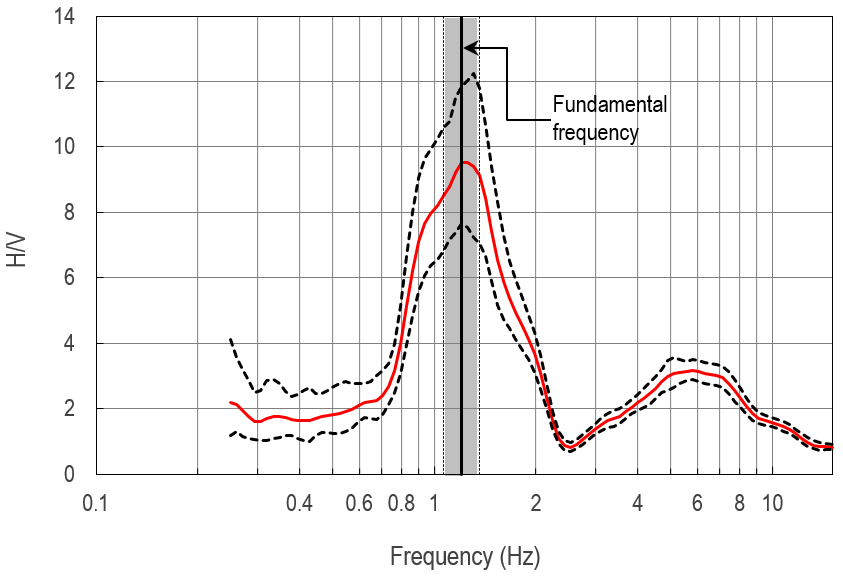 | | 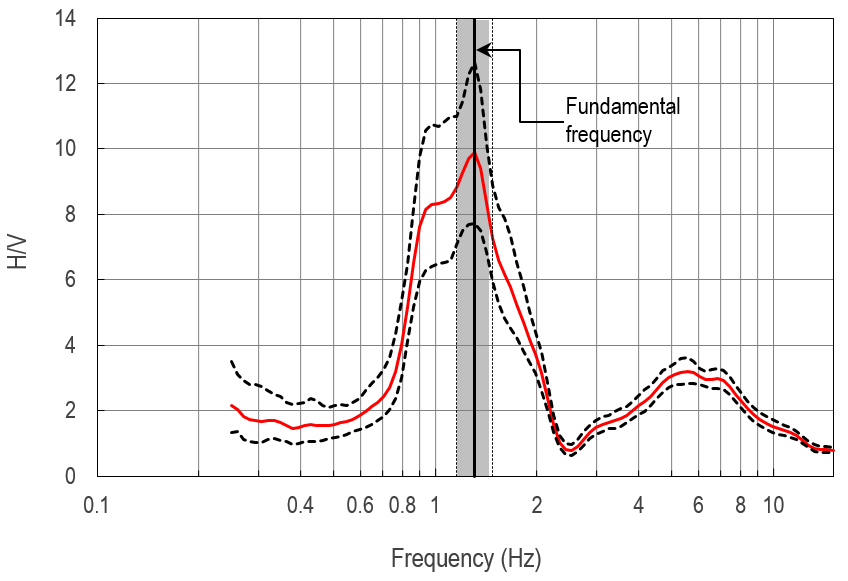 | |
| Fundamental frequency (Hz) | 1.204 | Fundamental frequency (Hz) | 1.307 |
| **Location 02 Instrument 01 Trial#02** | | **Location 02 Instrument 01 Trial#03** | |

| 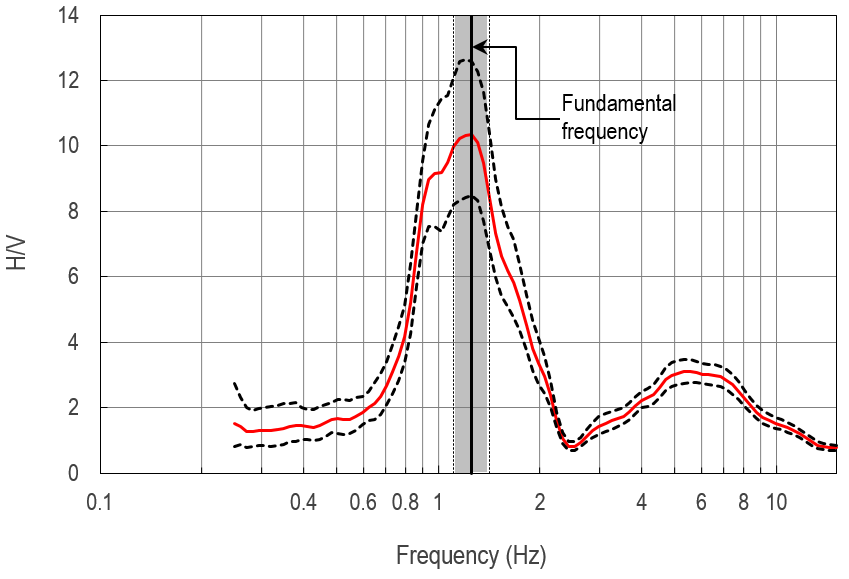 | | 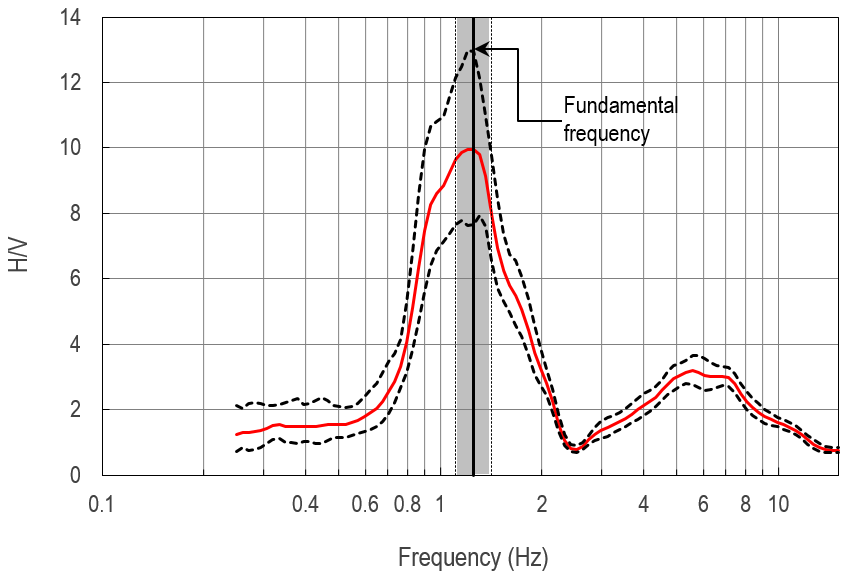 | |
| --- | --- | --- | --- |
| Fundamental frequency (Hz) | 1.254 | Fundamental frequency (Hz) | 1.254 |
| **Location 02 Instrument 01 Trial#04** | | **Location 02 Instrument 01 Trial#05** | |
| 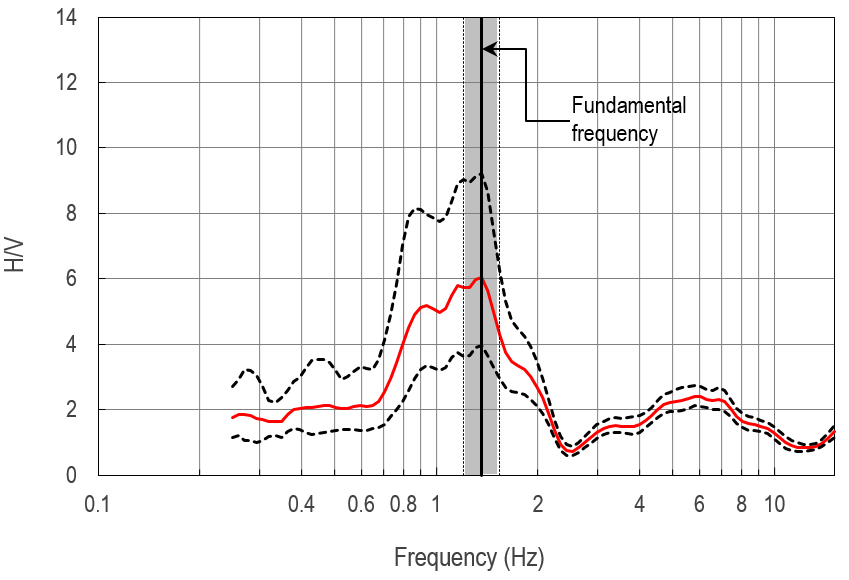 | | 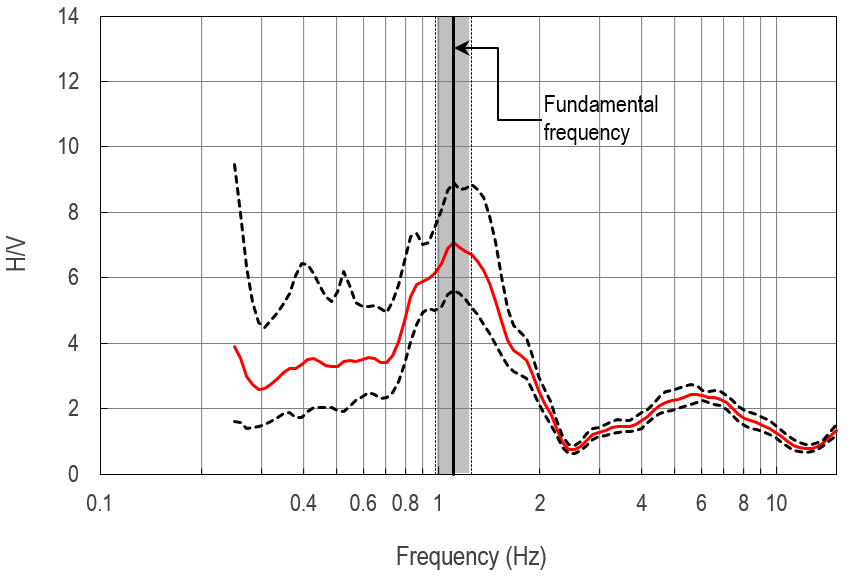 | |
| Fundamental frequency (Hz) | 1.363 | Fundamental frequency (Hz) | 1.108 |
| **Location 02 Instrument 02 Trial#01** | | **Location 02 Instrument 02 Trial#02** | |
| 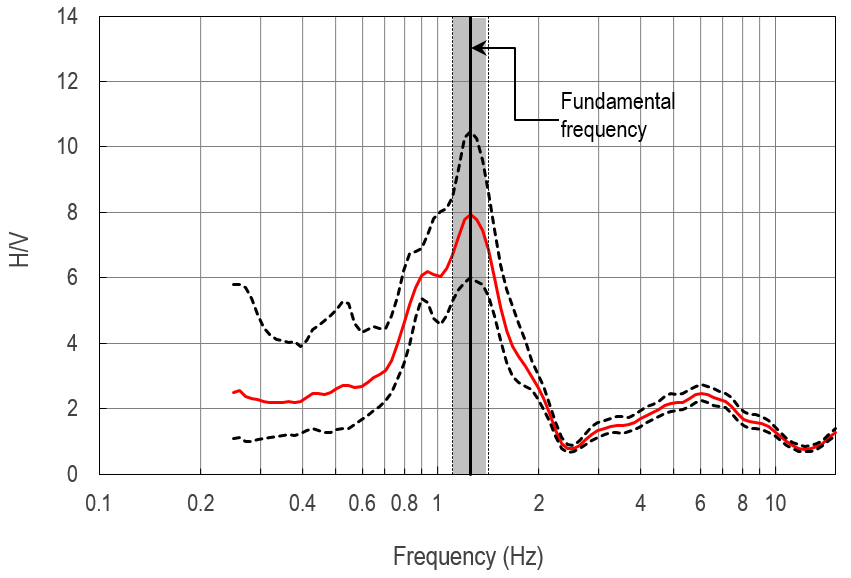 | | 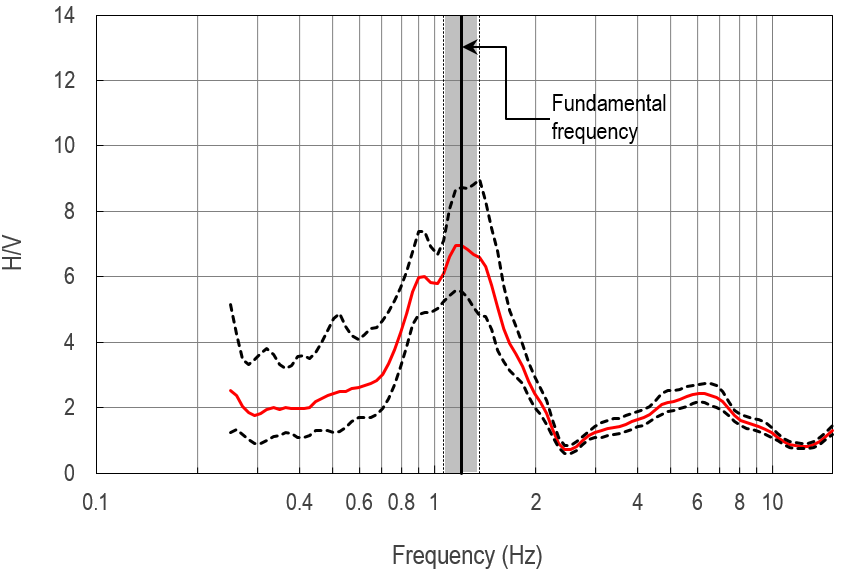 | |
| Fundamental frequency (Hz) | 1.254 | Fundamental frequency (Hz) | 1.204 |
| **Location 02 Instrument 02 Trial#03** | | **Location 02 Instrument 02 Trial#04** | |

| 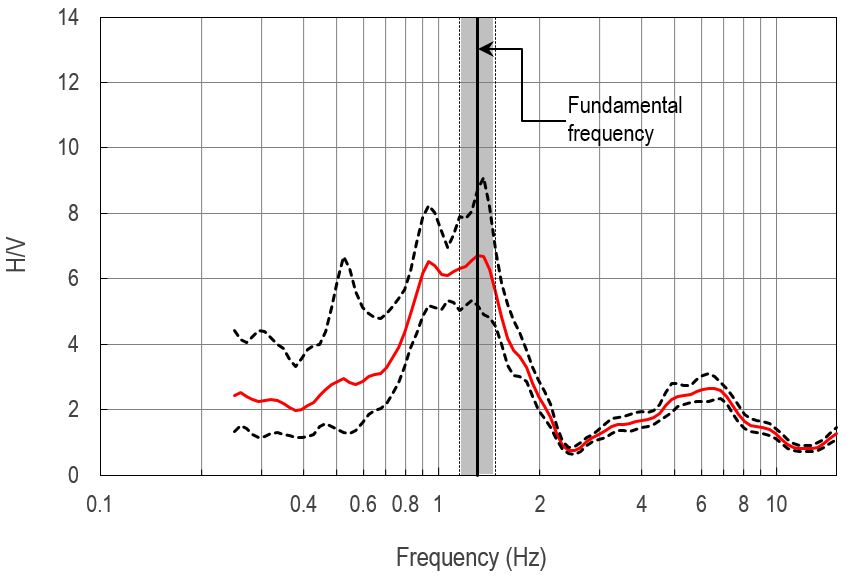 | | 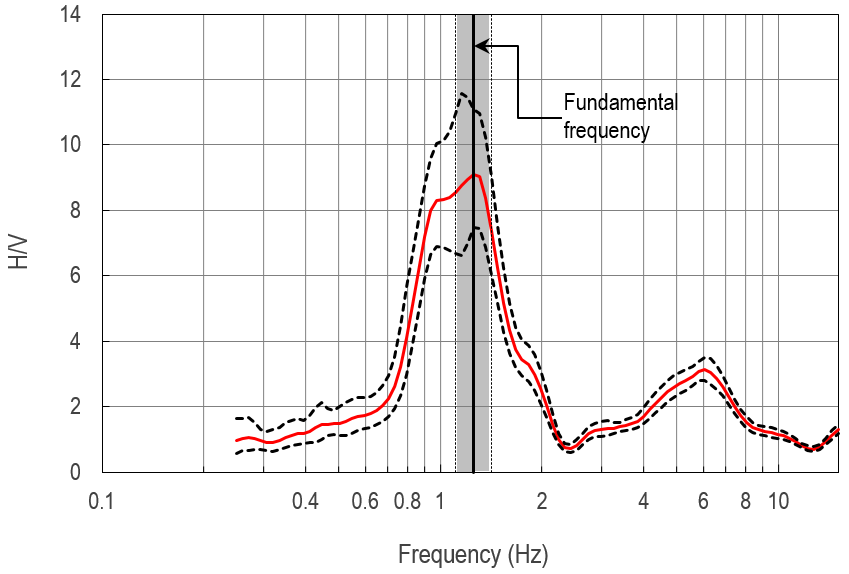 | |
| --- | --- | --- | --- |
| Fundamental frequency (Hz) | 1.307 | Fundamental frequency (Hz) | 1.254 |
| **Location 02 Instrument 02 Trial#05** | | **Location 02 Instrument 03 Trial#01** | |
| 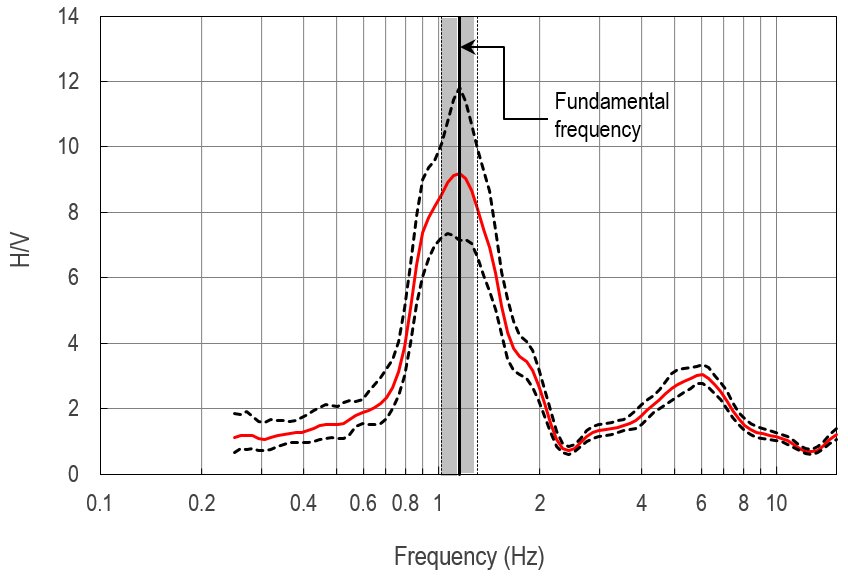 | | 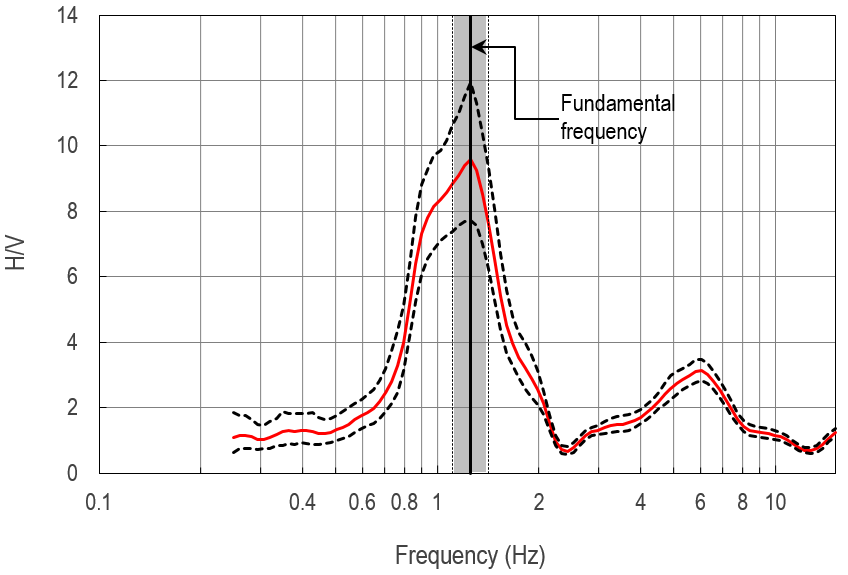 | |
| Fundamental frequency (Hz) | 1.154 | Fundamental frequency (Hz) | 1.254 |
| **Location 02 Instrument 03 Trial#02** | | **Location 02 Instrument 03 Trial#03** | |
| 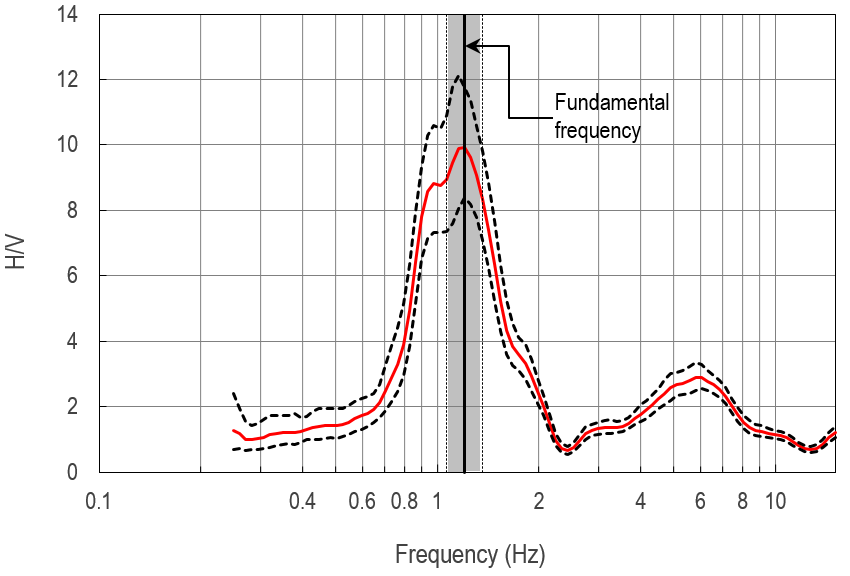 | | 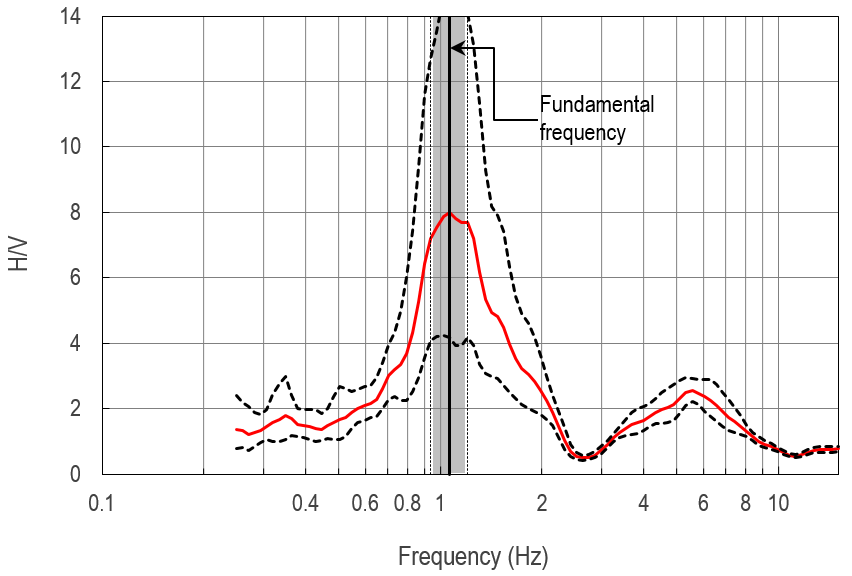 | |
| Fundamental frequency (Hz) | 1.204 | Fundamental frequency (Hz) | 1.063 |
| **Location 02 Instrument 03 Trial#04** | | **Location 03 Instrument 01 Trial#01** | |

| 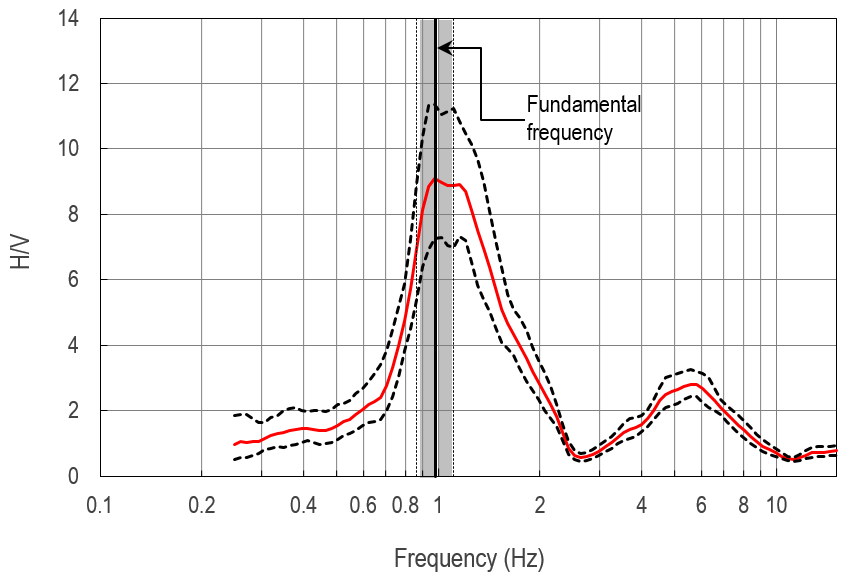 | | 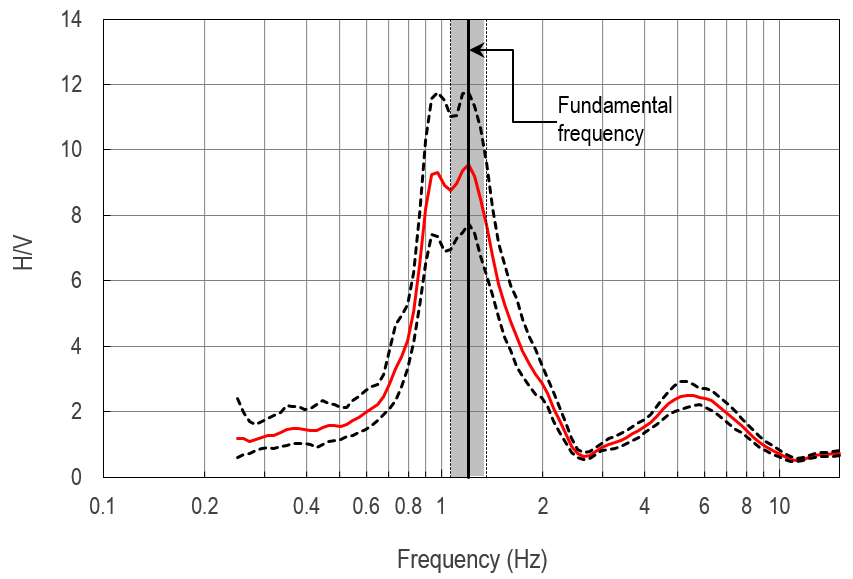 | |
| --- | --- | --- | --- |
| Fundamental frequency (Hz) | 0.978 | Fundamental frequency (Hz) | 1.204 |
| **Location 03 Instrument 01 Trial#02** | | **Location 03 Instrument 01 Trial#03** | |
| 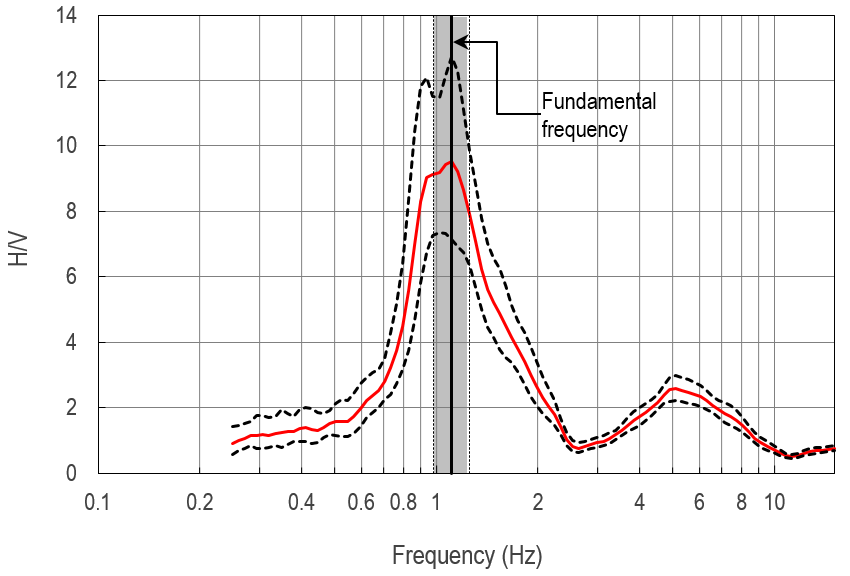 | | 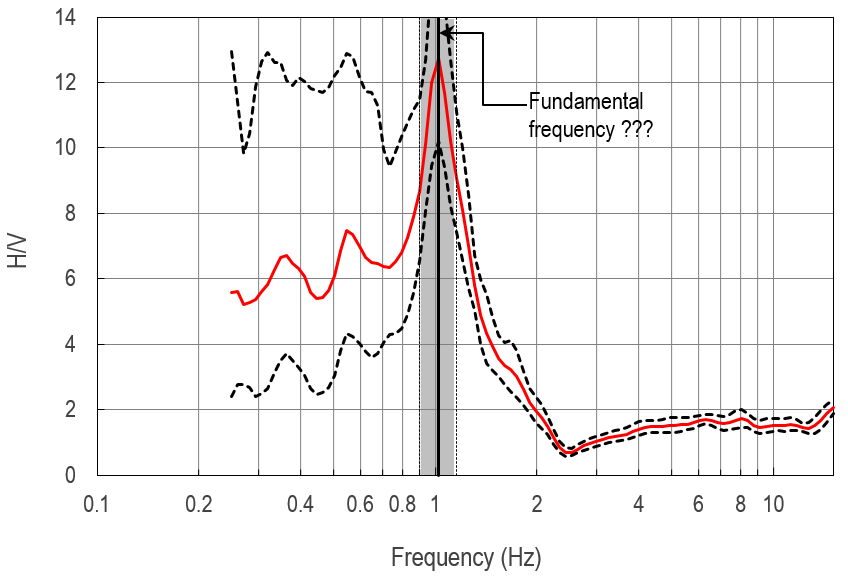 | |
| Fundamental frequency (Hz) | 1.108 | Fundamental frequency (Hz) | 1.020??? |
| **Location 03 Instrument 01 Trial#04** | | **Location 04 Instrument 01 Trial#01** | |
| 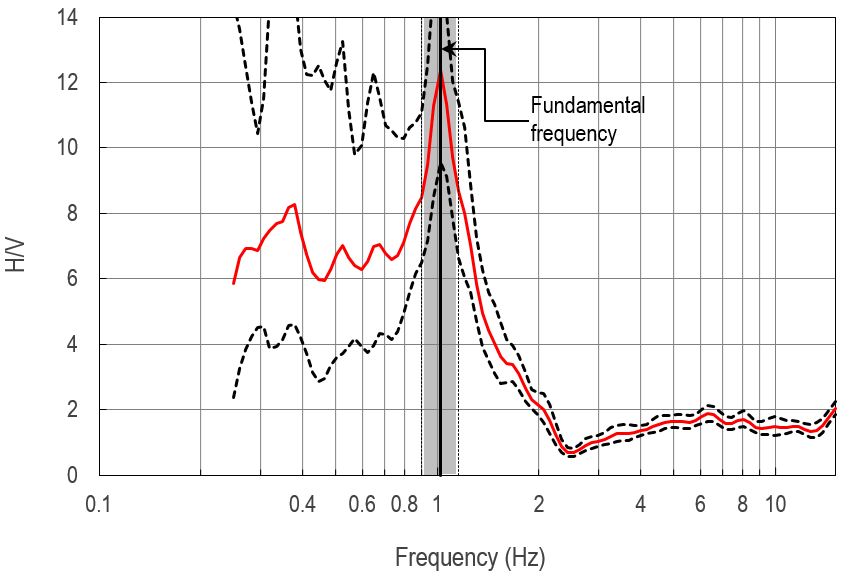 | | 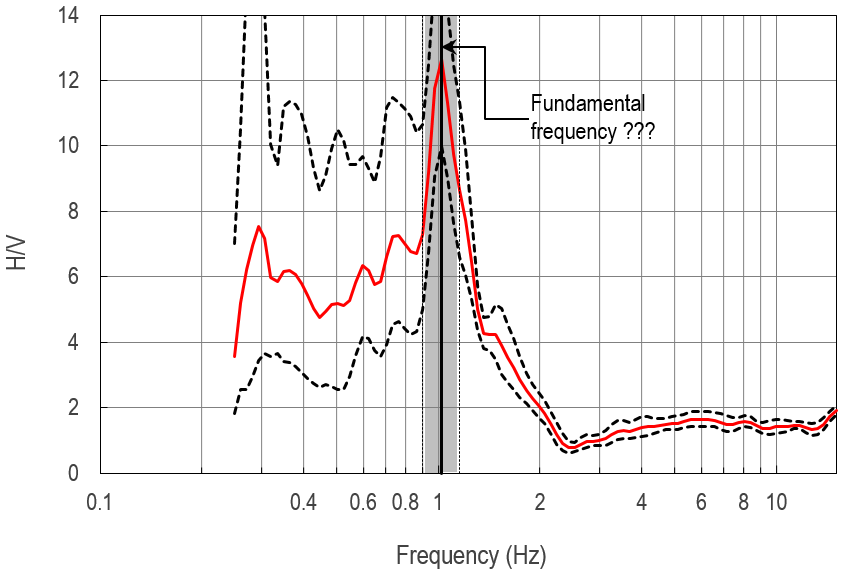 | |
| Fundamental frequency (Hz) | 1.020 | Fundamental frequency (Hz) | 1.020??? |
| **Location 04 Instrument 01 Trial#02** | | **Location 04 Instrument 01 Trial#03** | |

| 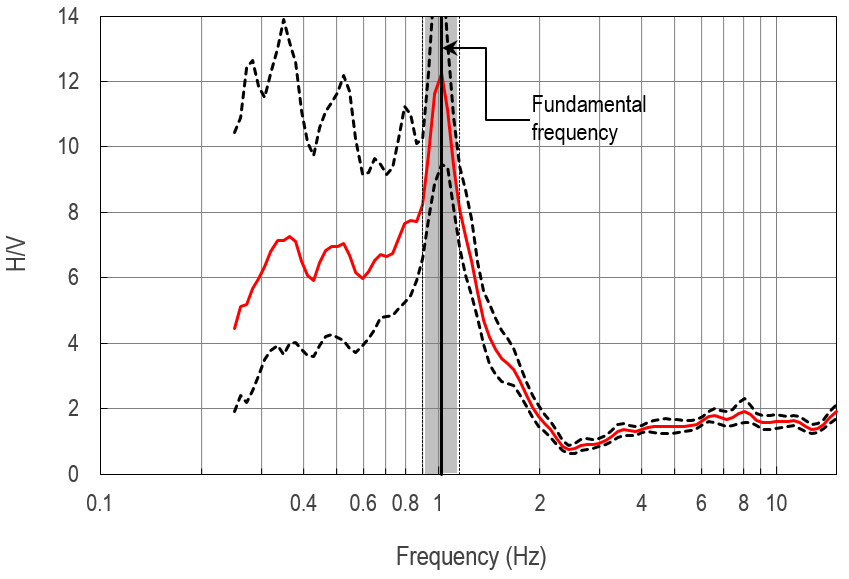 | | 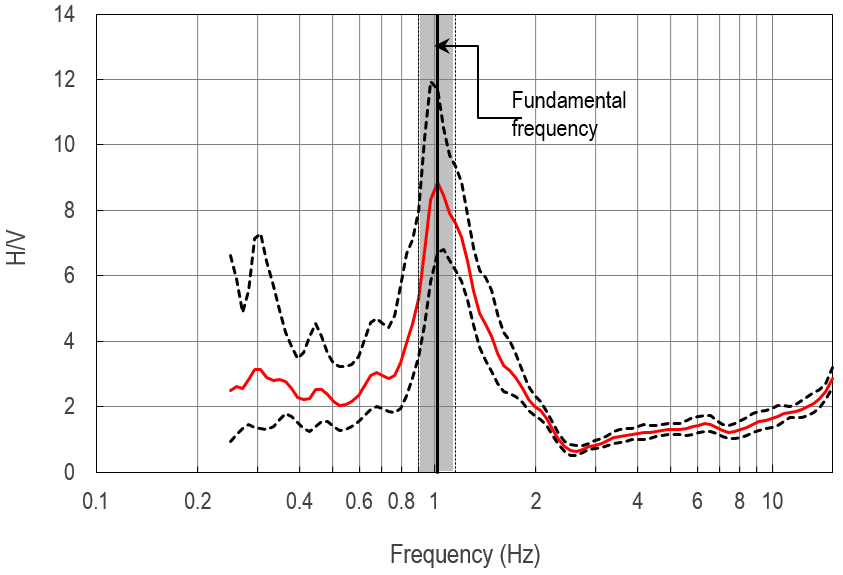 | |
| --- | --- | --- | --- |
| Fundamental frequency (Hz) | 1.020 | Fundamental frequency (Hz) | 1.020 |
| **Location 04 Instrument 01 Trial#04** | | **Location 04 Instrument 02 Trial#01** | |
| 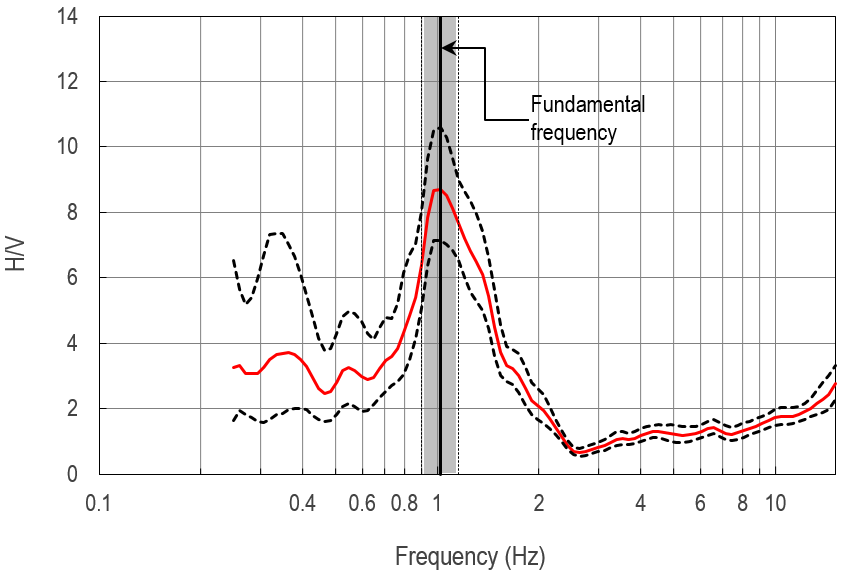 | | 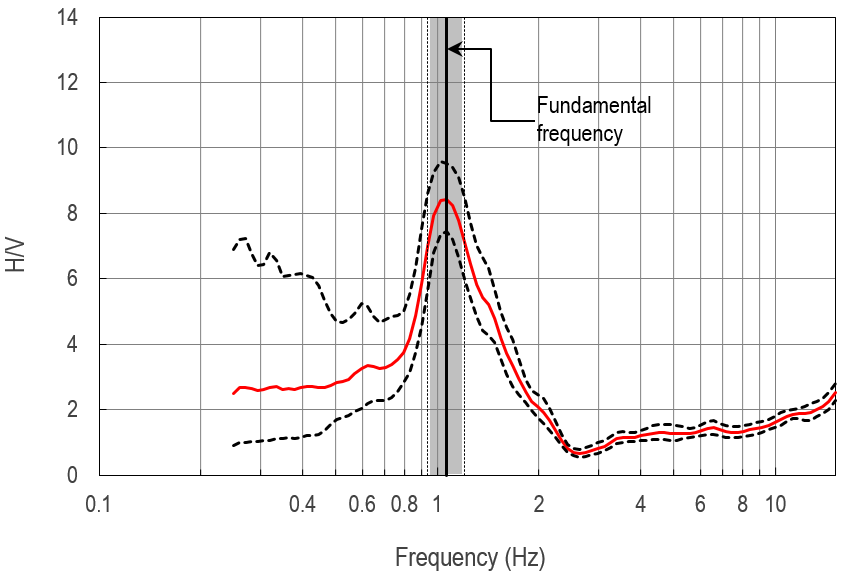 | |
| Fundamental frequency (Hz) | 1.020 | Fundamental frequency (Hz) | 1.063 |
| **Location 04 Instrument 02 Trial#02** | | **Location 04 Instrument 02 Trial#03** | |
| 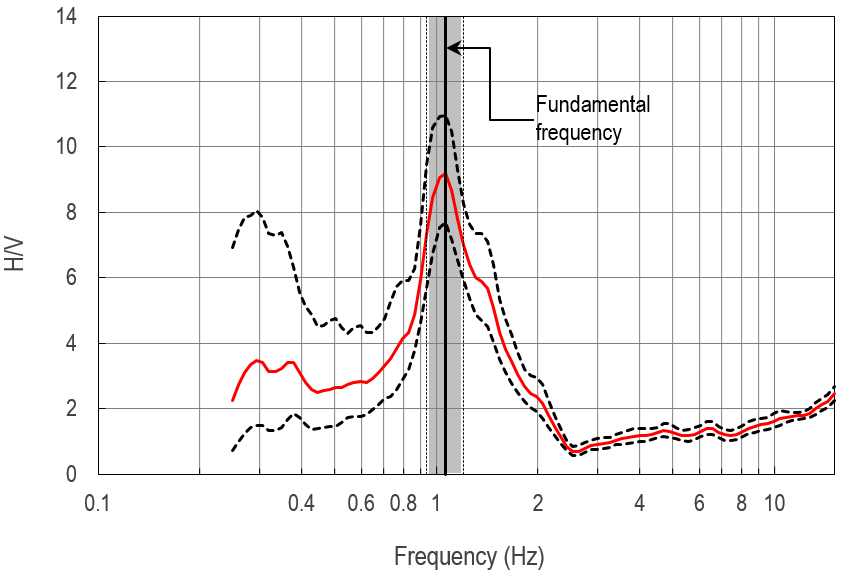 | | 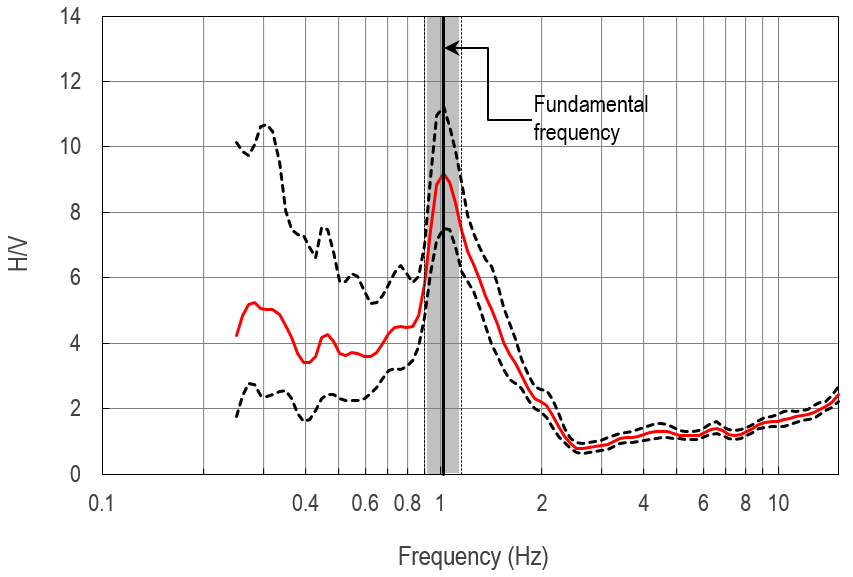 | |
| Fundamental frequency (Hz) | 1.063 | Fundamental frequency (Hz) | 1.020 |
| **Location 04 Instrument 02 Trial#04** | | **Location 04 Instrument 02 Trial#05** | |

| 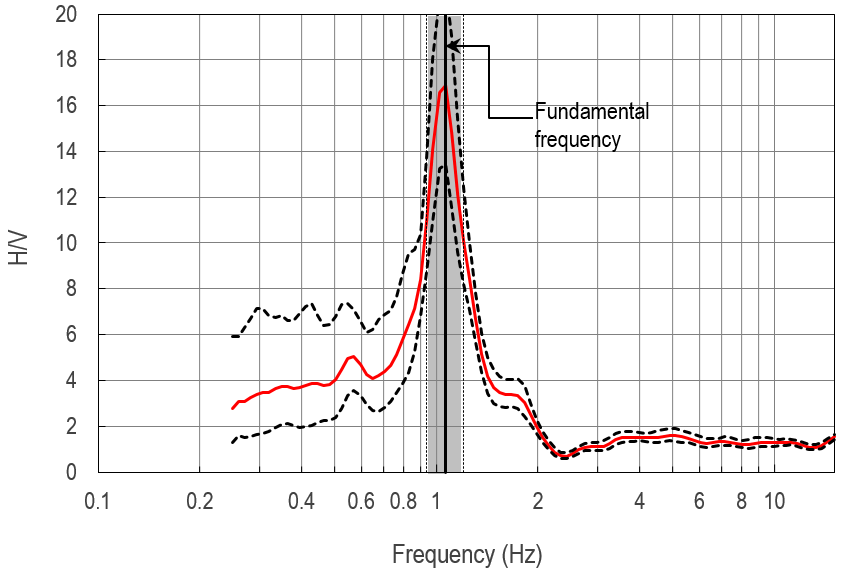 | | 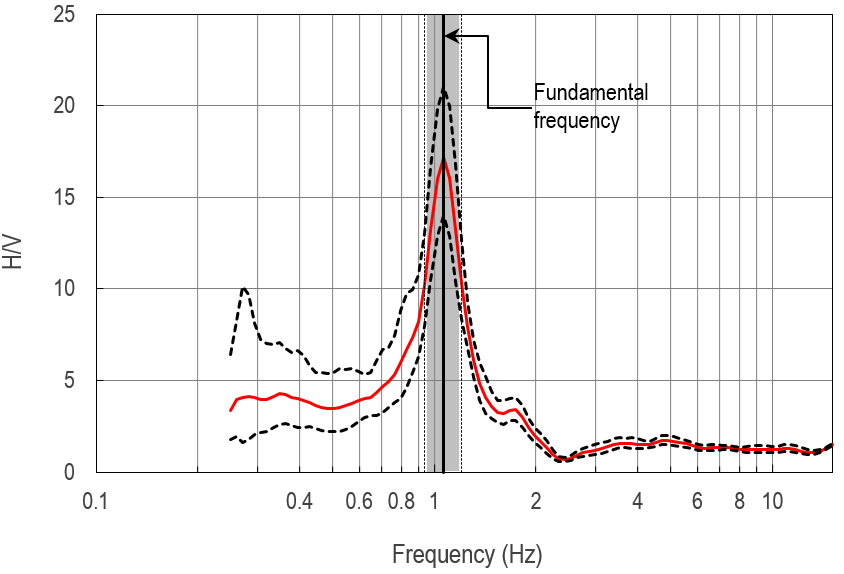 | |
| --- | --- | --- | --- |
| Fundamental frequency (Hz) | 1.063 | Fundamental frequency (Hz) | 1.063 |
| **Location 04 Instrument 03 Trial#01** | | **Location 04 Instrument 03 Trial#02** | |
| 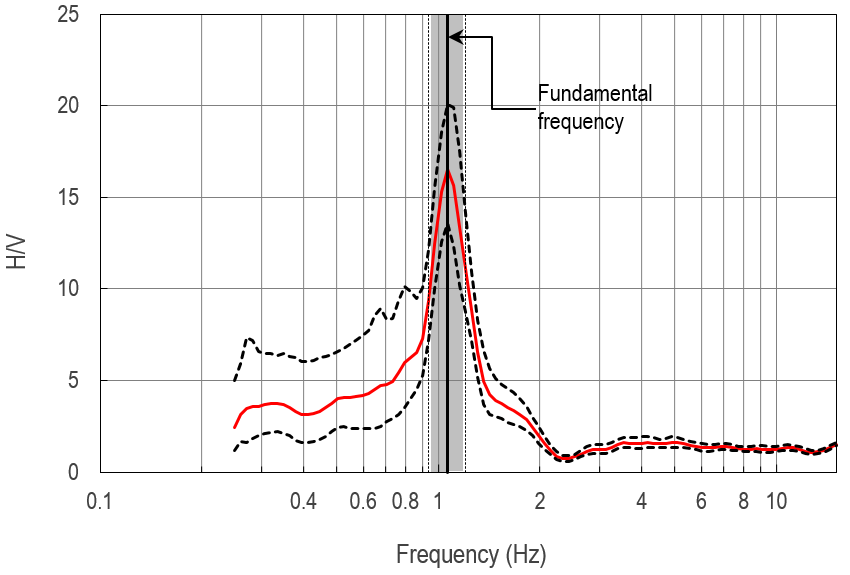 | | 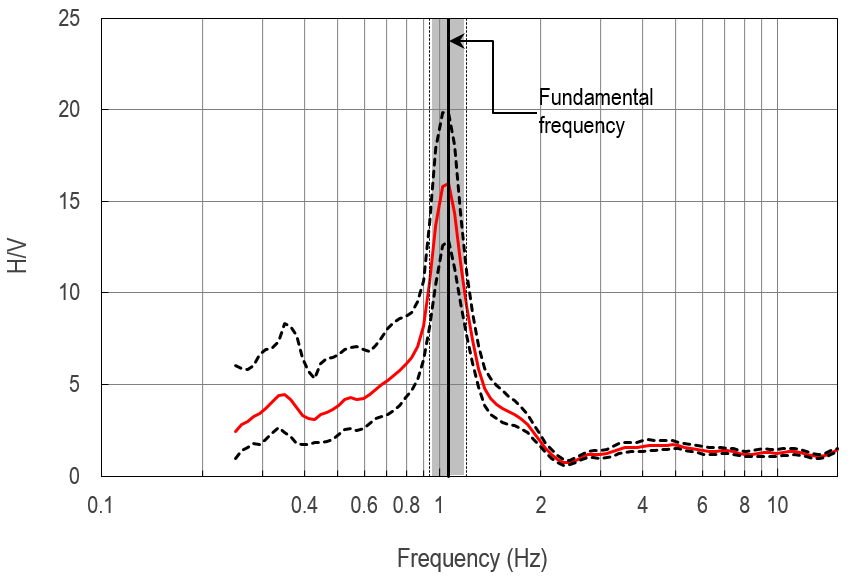 | |
| Fundamental frequency (Hz) | 1.063 | Fundamental frequency (Hz) | 1.063 |
| **Location 04 Instrument 03 Trial#03** | | **Location 04 Instrument 03 Trial#04** | |
| 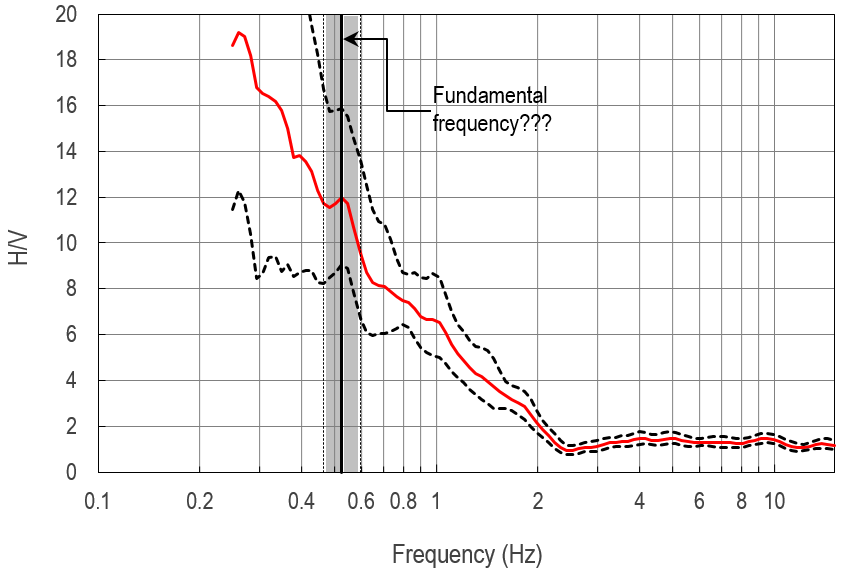 | | 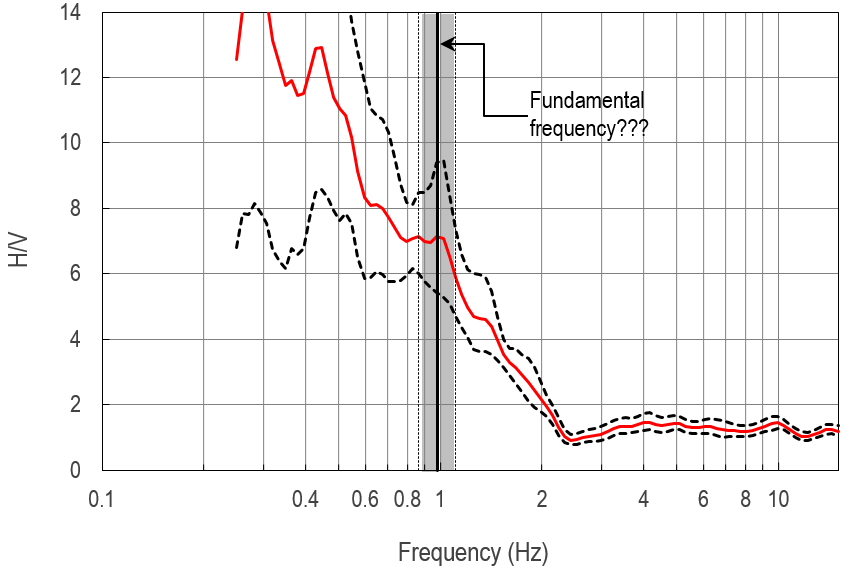 | |
| Fundamental frequency (Hz) | 0.526??? | Fundamental frequency (Hz) | 0.978??? |
| **Location 05 Instrument 01 Trial#01** | | **Location 05 Instrument 01 Trial#02** | |

| 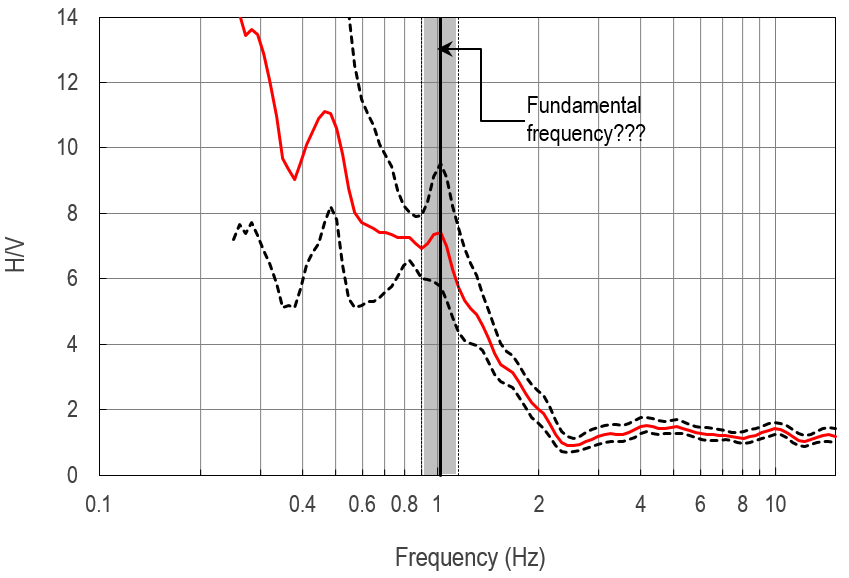 | | 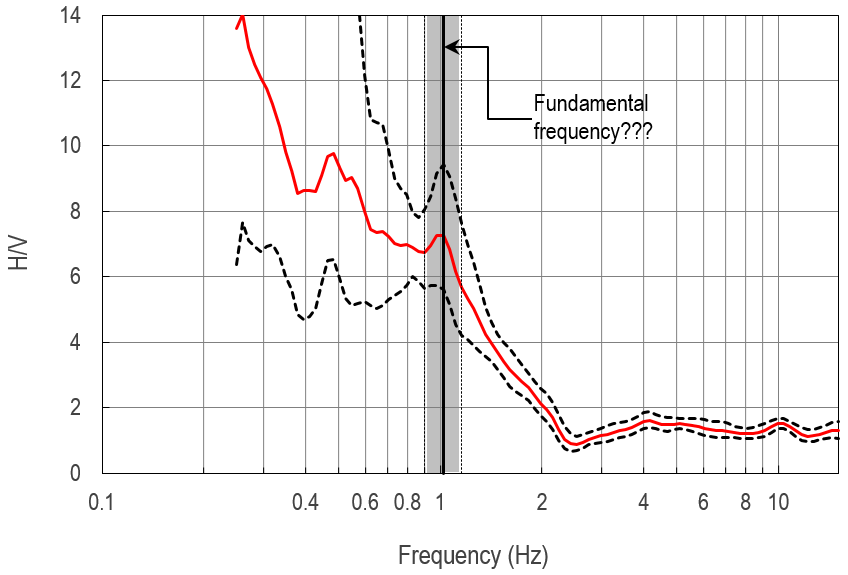 | |
| --- | --- | --- | --- |
| Fundamental frequency (Hz) | 1.020??? | Fundamental frequency (Hz) | 1.020??? |
| **Location 05 Instrument 01 Trial#03** | | **Location 05 Instrument 01 Trial#04** | |
| 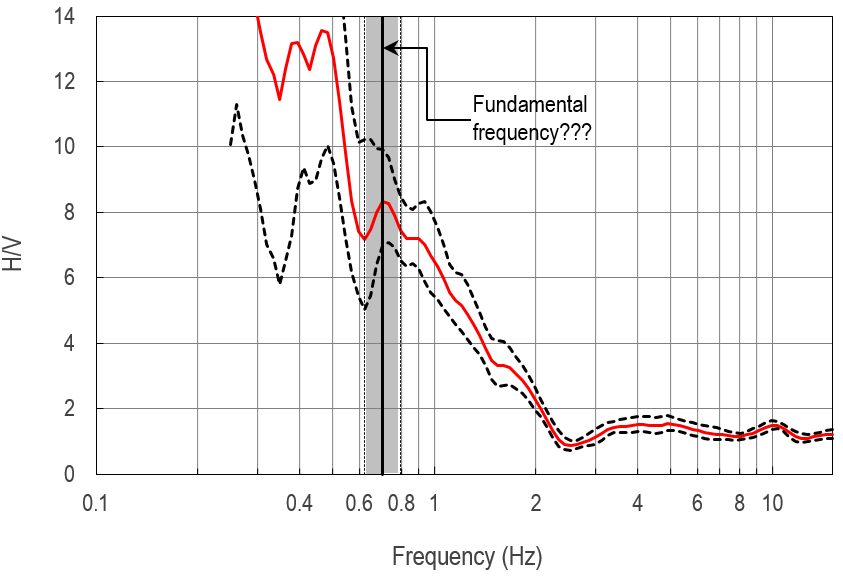 | | 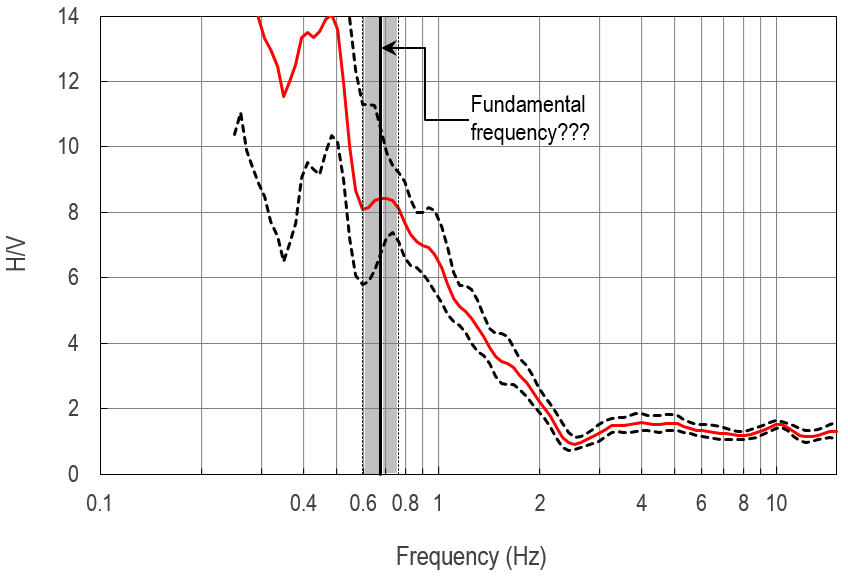 | |
| Fundamental frequency (Hz) | 0.703??? | Fundamental frequency (Hz) | 0.675??? |
| **Location 05 Instrument 01 Trial#05** | | **Location 05 Instrument 01 Trial#06** | |
| 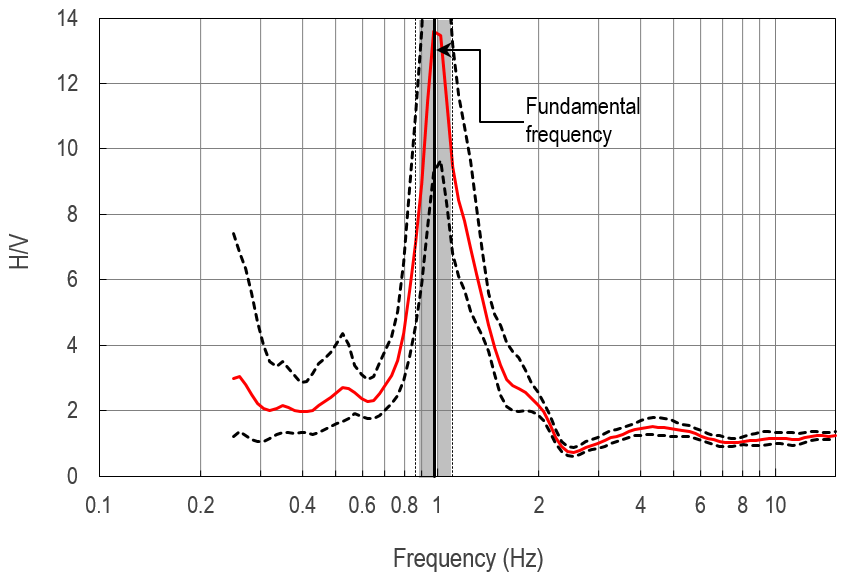 | | 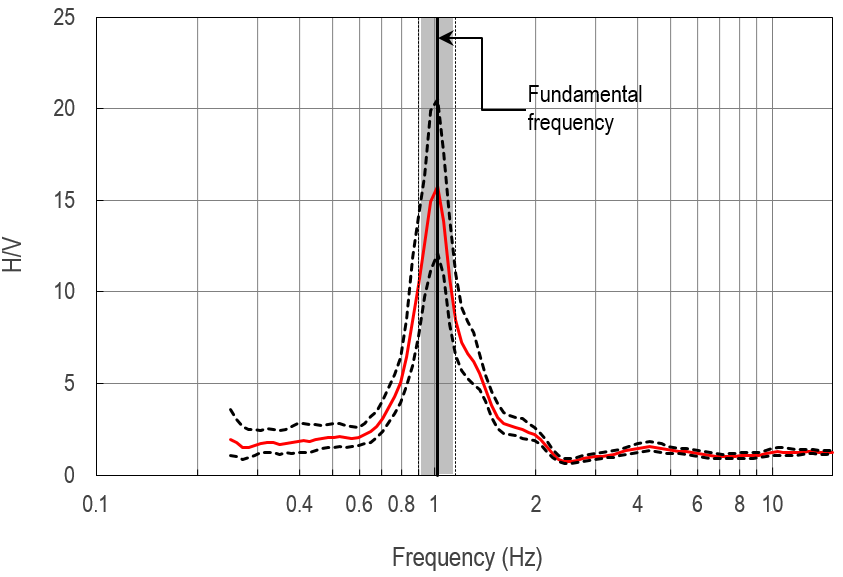 | |
| Fundamental frequency (Hz) | 0.979 | Fundamental frequency (Hz) | 1.020 |
| **Location 05 Instrument 02 Trial#01** | | **Location 05 Instrument 02 Trial#02** | |

| 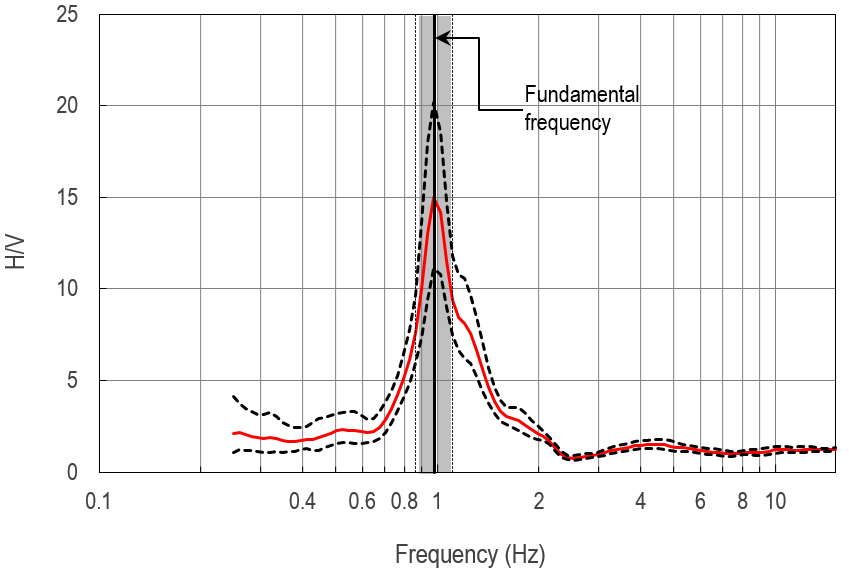 | | 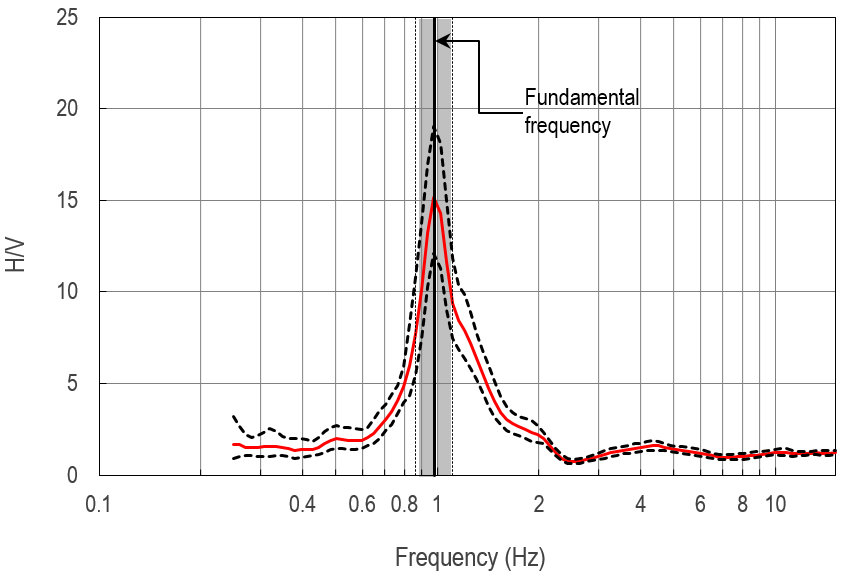 | |
| --- | --- | --- | --- |
| Fundamental frequency (Hz) | 0.979 | Fundamental frequency (Hz) | 0.979 |
| **Location 05 Instrument 02 Trial#03** | | **Location 05 Instrument 02 Trial#04** | |
| 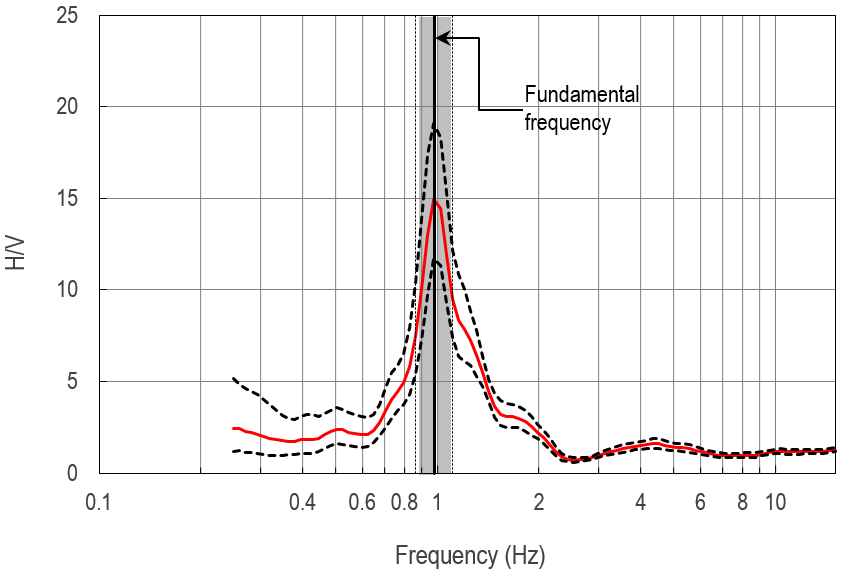 | | 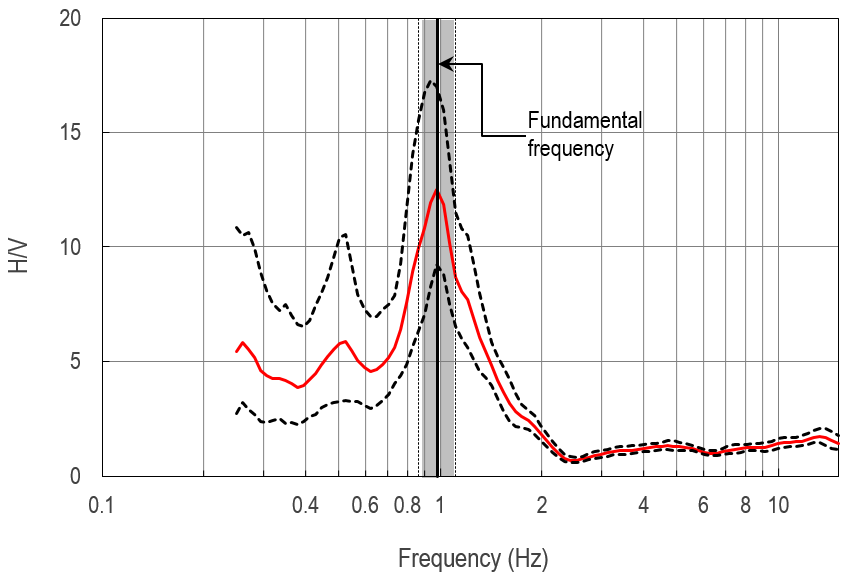 | |
| Fundamental frequency (Hz) | 0.979 | Fundamental frequency (Hz) | 0.979 |
| **Location 05 Instrument 02 Trial#05** | | **Location 05 Instrument 03 Trial#01** | |
| 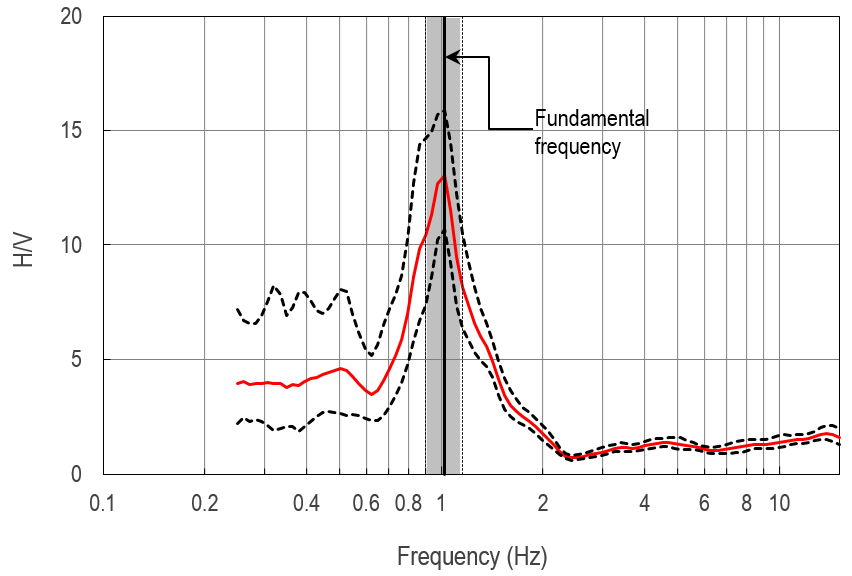 | | 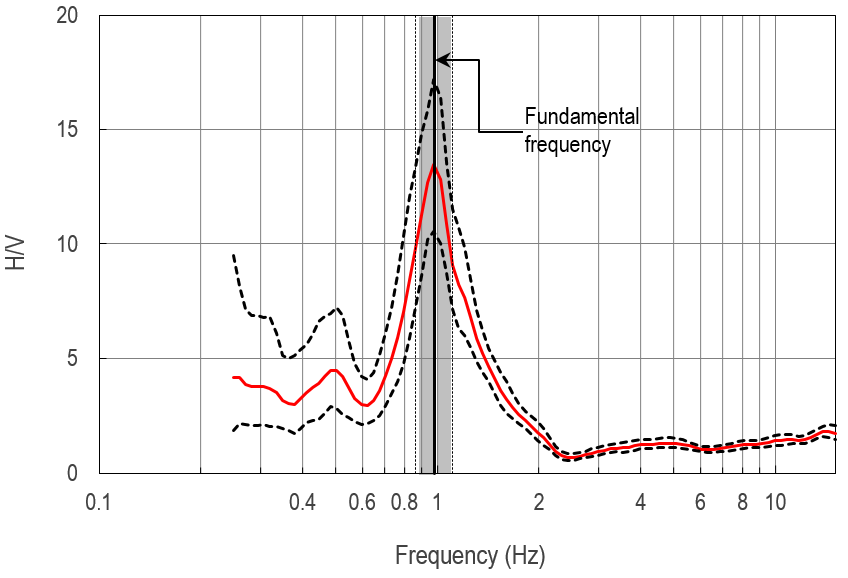 | |
| Fundamental frequency (Hz) | 1.020 | Fundamental frequency (Hz) | 0.979 |
| **Location 05 Instrument 03 Trial#02** | | **Location 05 Instrument 03 Trial#03** | |

| 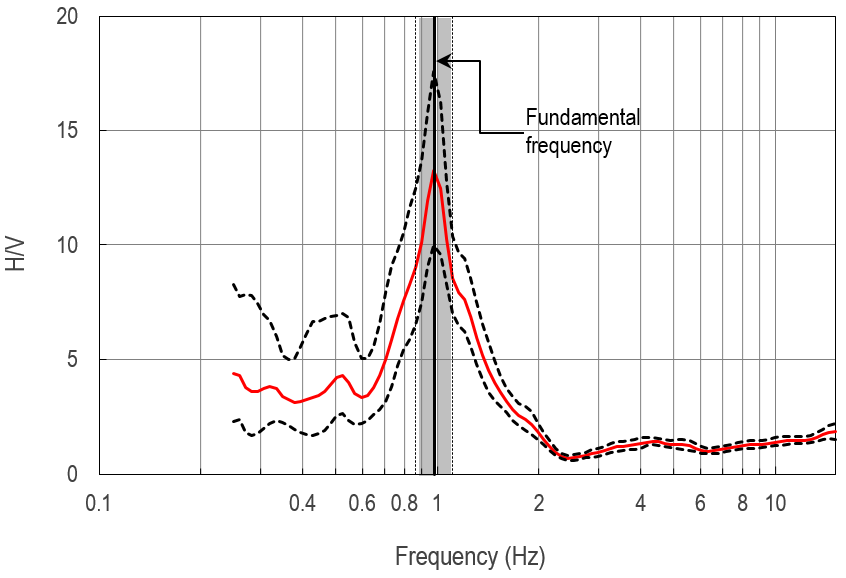 | | 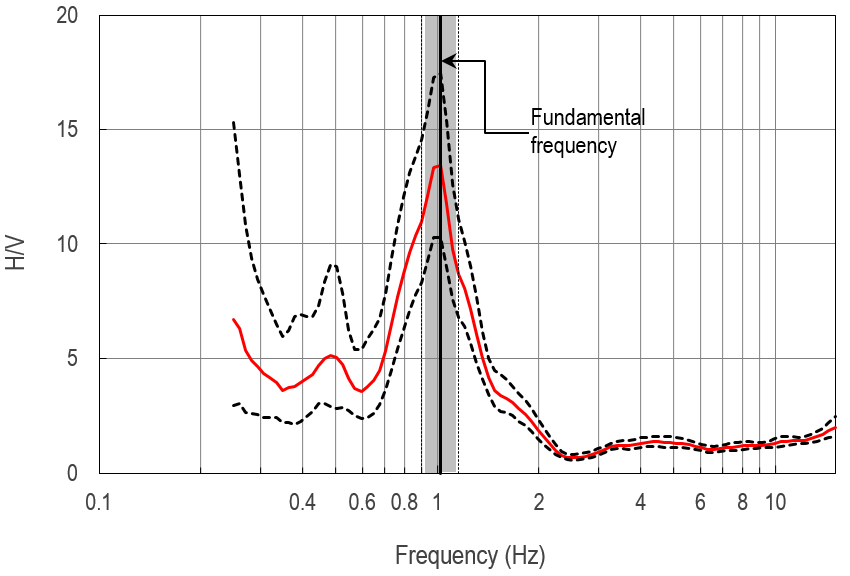 | |
| --- | --- | --- | --- |
| Fundamental frequency (Hz) | 0.979 | Fundamental frequency (Hz) | 1.020 |
| **Location 05 Instrument 03 Trial#04** | | **Location 05 Instrument 03 Trial#05** | |
| 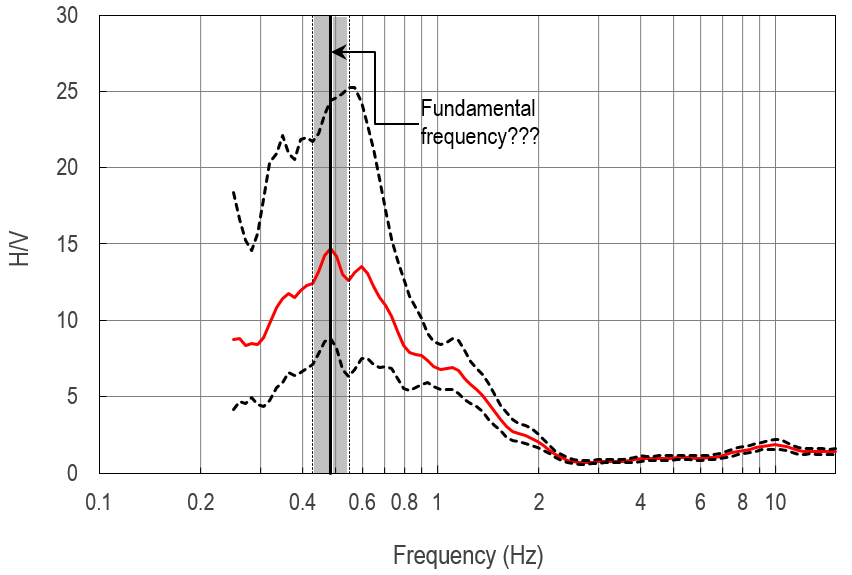 | | 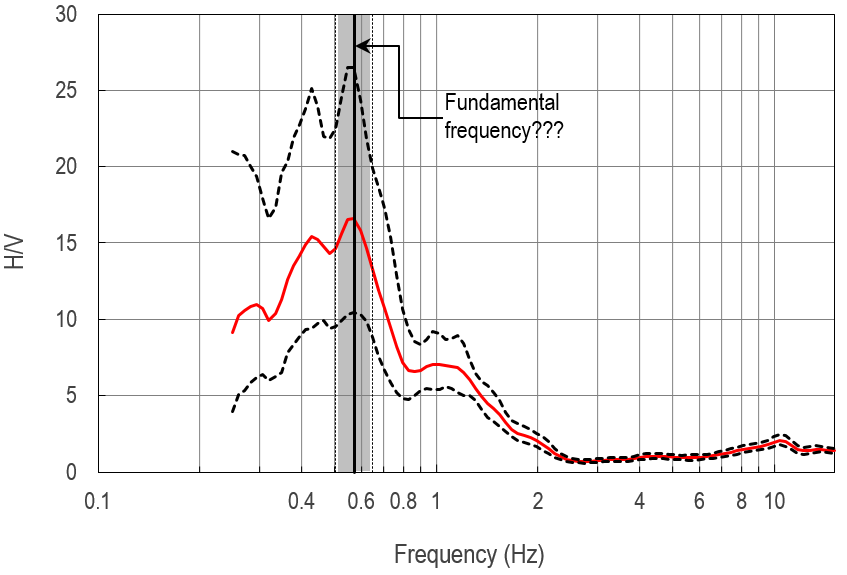 | |
| Fundamental frequency (Hz) | 0.485??? | Fundamental frequency (Hz) | 0.572??? |
| **Location 06 Instrument 01 Trial#01** | | **Location 06 Instrument 01 Trial#02** | |
| 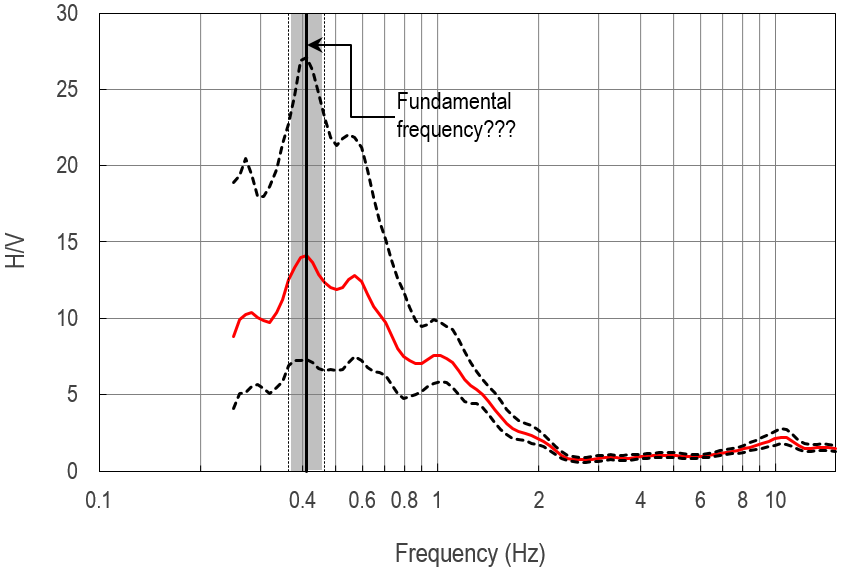 | | 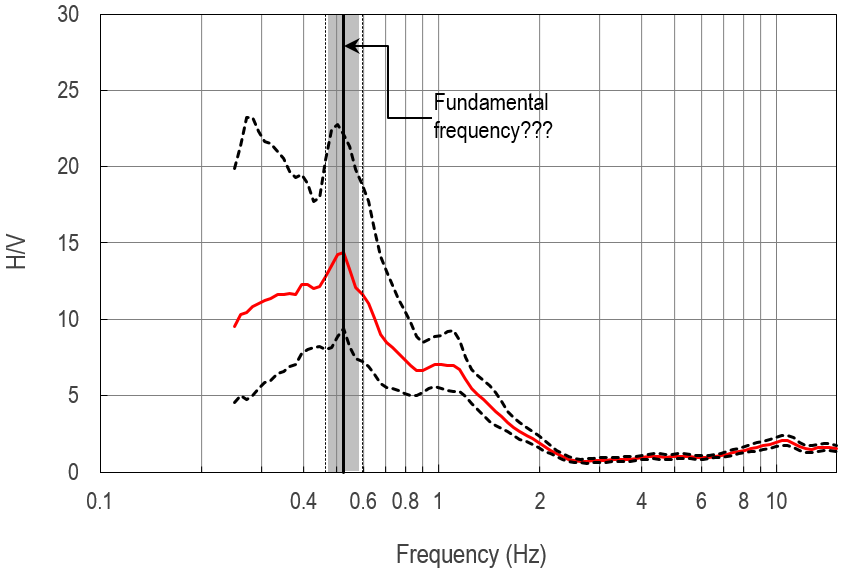 | |
| Fundamental frequency (Hz) | 0.411??? | Fundamental frequency (Hz) | 0.526??? |
| **Location 06 Instrument 01 Trial#03** | | **Location 06 Instrument 01 Trial#04** | |

| 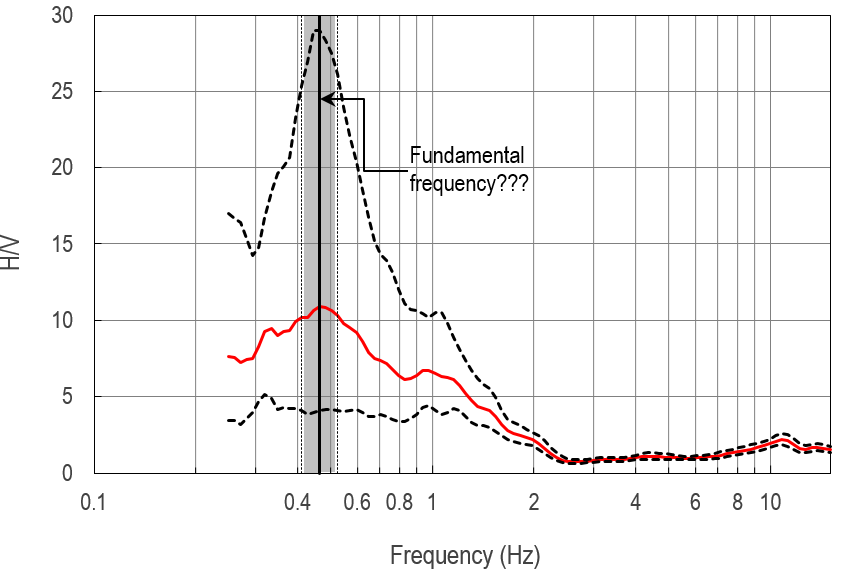 | | 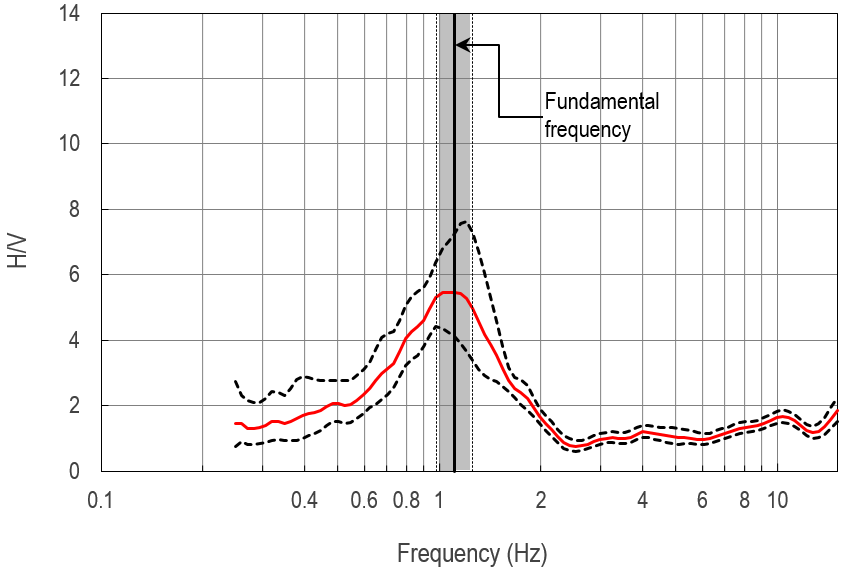 | |
| --- | --- | --- | --- |
| Fundamental frequency (Hz) | 0.465??? | Fundamental frequency (Hz) | 1.108 |
| **Location 06 Instrument 01 Trial#05** | | **Location 06 Instrument 02 Trial#01** | |
| 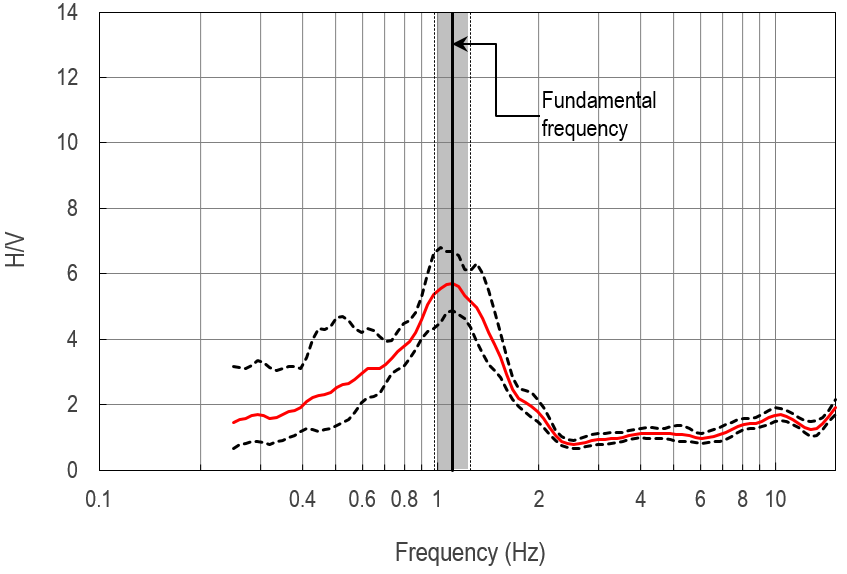 | | 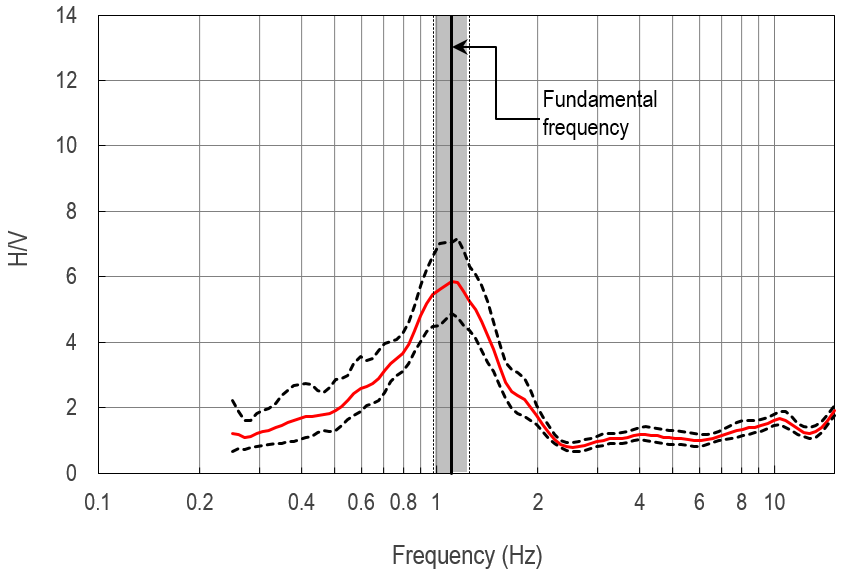 | |
| Fundamental frequency (Hz) | 1.108 | Fundamental frequency (Hz) | 1.108 |
| **Location 06 Instrument 02 Trial#02** | | **Location 06 Instrument 02 Trial#03** | |
| 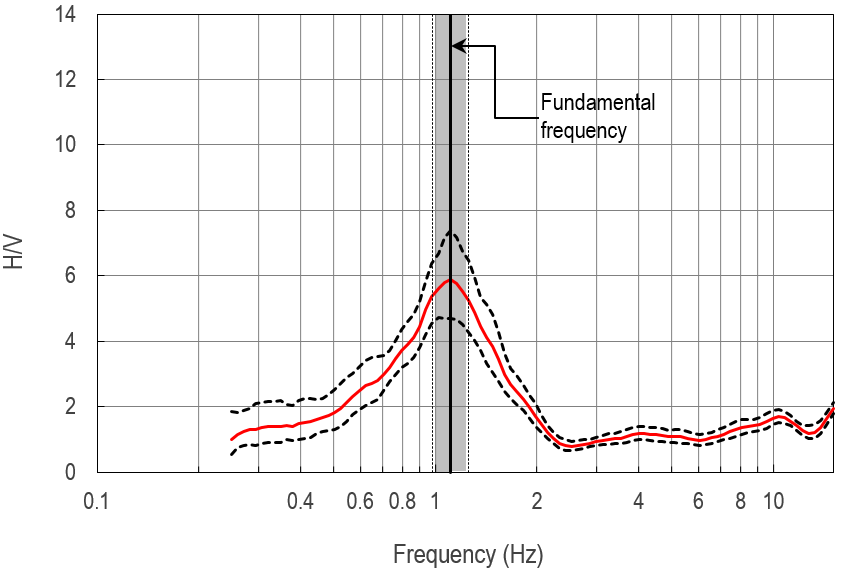 | | 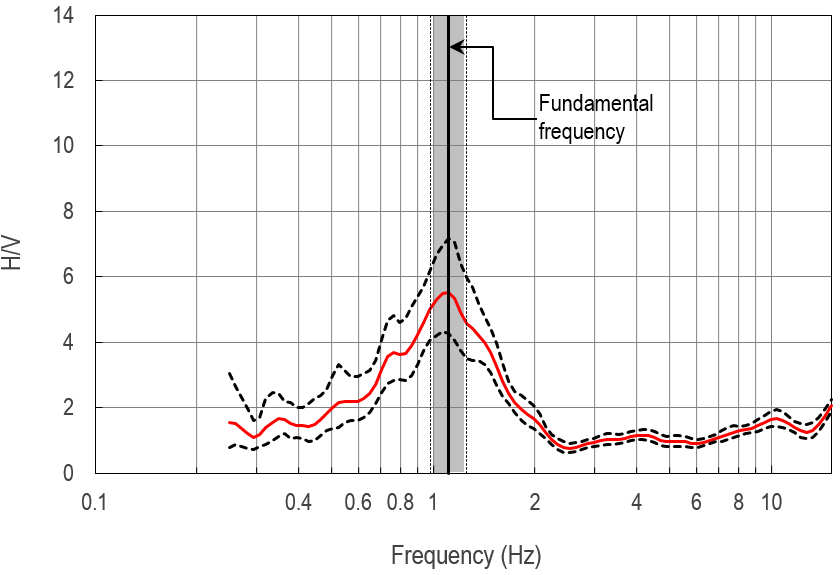 | |
| Fundamental frequency (Hz) | 1.108 | Fundamental frequency (Hz) | 1.108 |
| **Location 06 Instrument 02 Trial#04** | | **Location 06 Instrument 02 Trial#05** | |

| 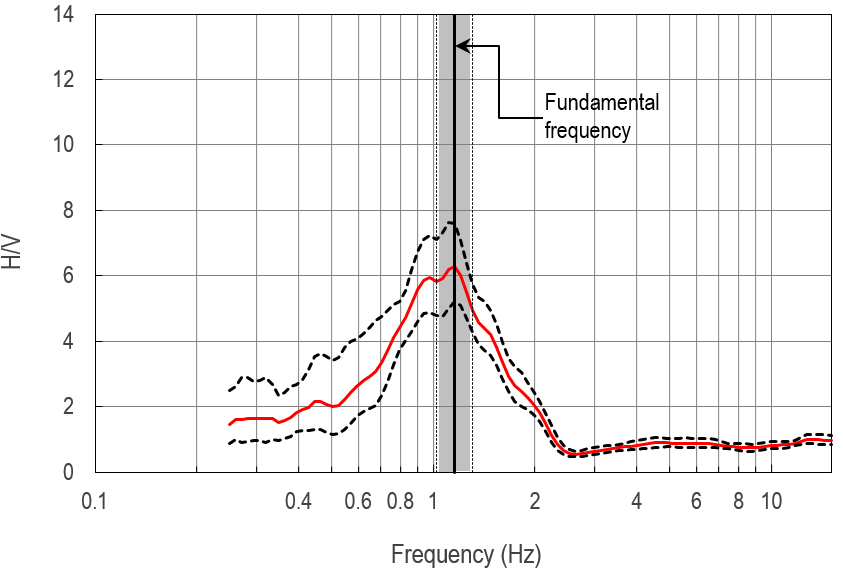 | | 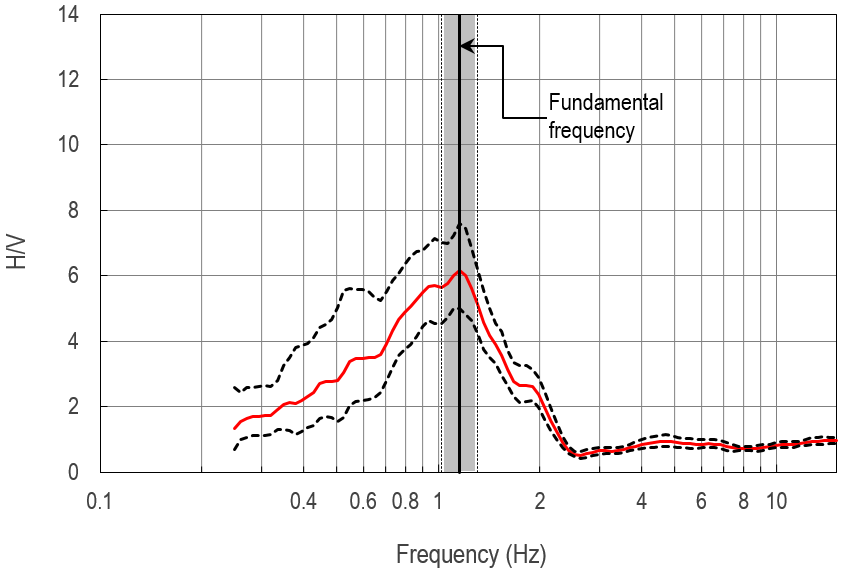 | |
| --- | --- | --- | --- |
| Fundamental frequency (Hz) | 1.155 | Fundamental frequency (Hz) | 1.155 |
| **Location 06 Instrument 03 Trial#01** | | **Location 06 Instrument 03 Trial#02** | |
| 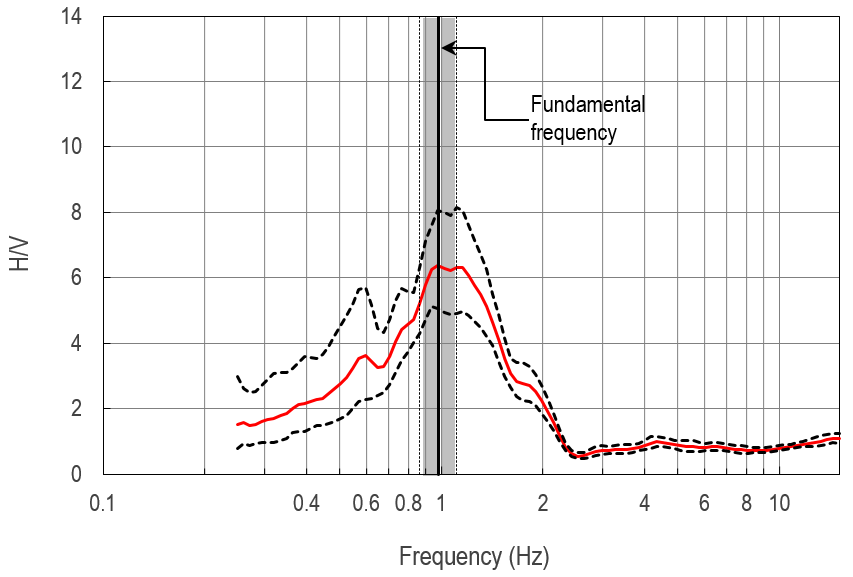 | | 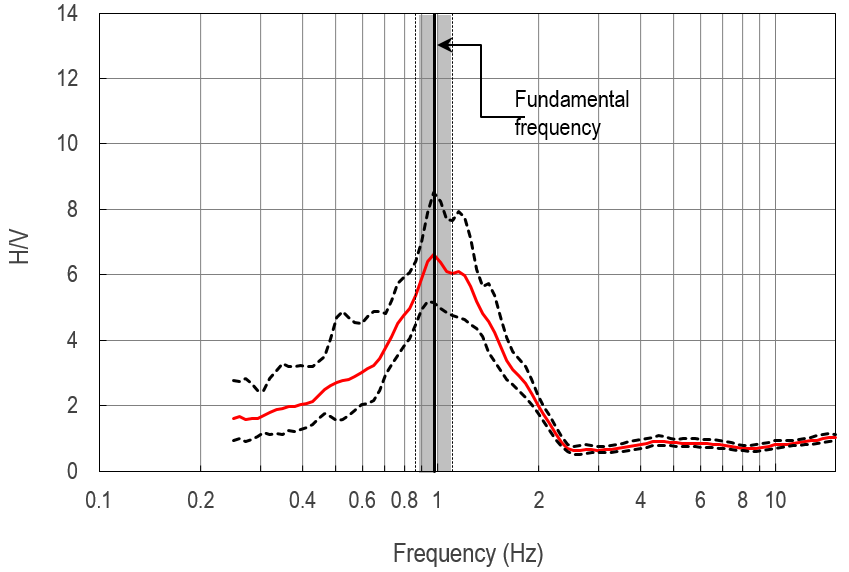 | |
| Fundamental frequency (Hz) | 0.979 | Fundamental frequency (Hz) | 0.979 |
| **Location 06 Instrument 03 Trial#03** | | **Location 06 Instrument 03 Trial#04** | |
| 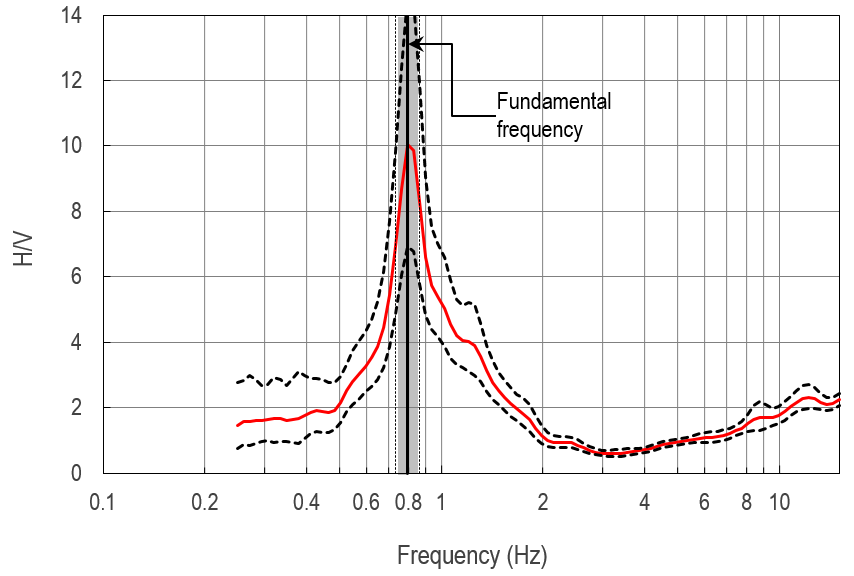 | | 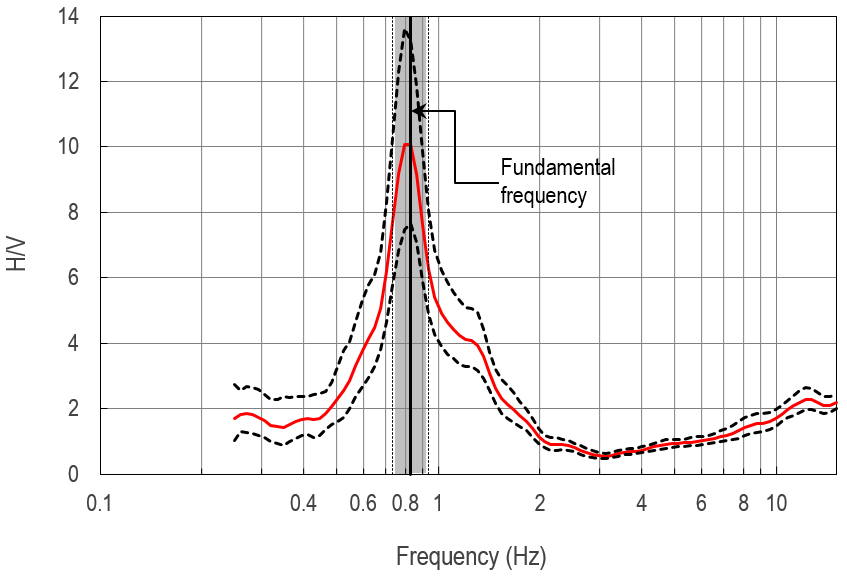 | |
| Fundamental frequency (Hz) | 0.796 | Fundamental frequency (Hz) | 0.829 |
| **Location 07 Instrument 01 Trial#01** | | **Location 01 Instrument 07 Trial#02** | |

| 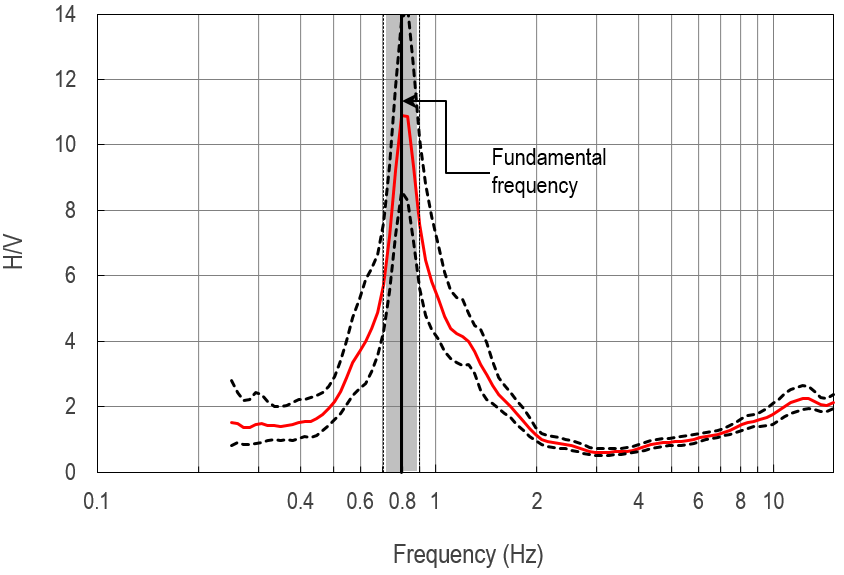 | | 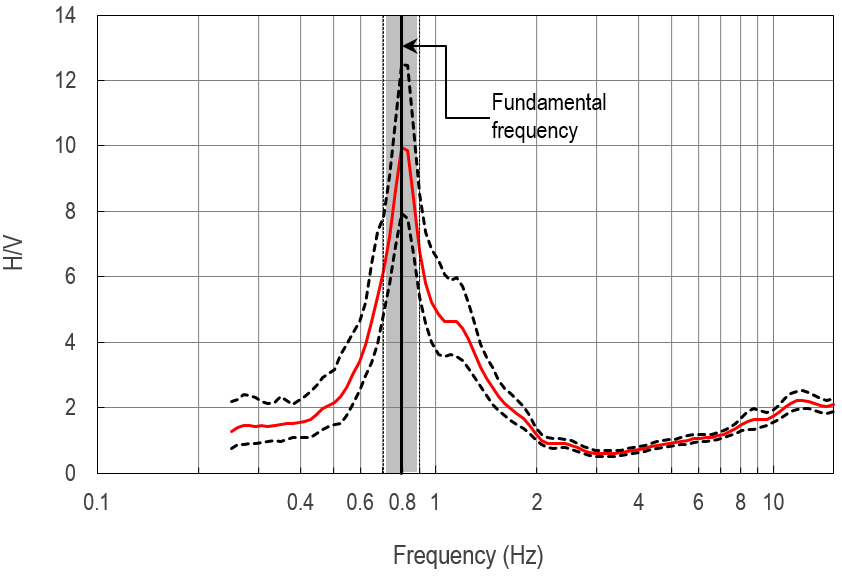 | |
| --- | --- | --- | --- |
| Fundamental frequency (Hz) | 0.796 | Fundamental frequency (Hz) | 0.796 |
| **Location 07 Instrument 01 Trial#03** | | **Location 01 Instrument 07 Trial#04** | |
| 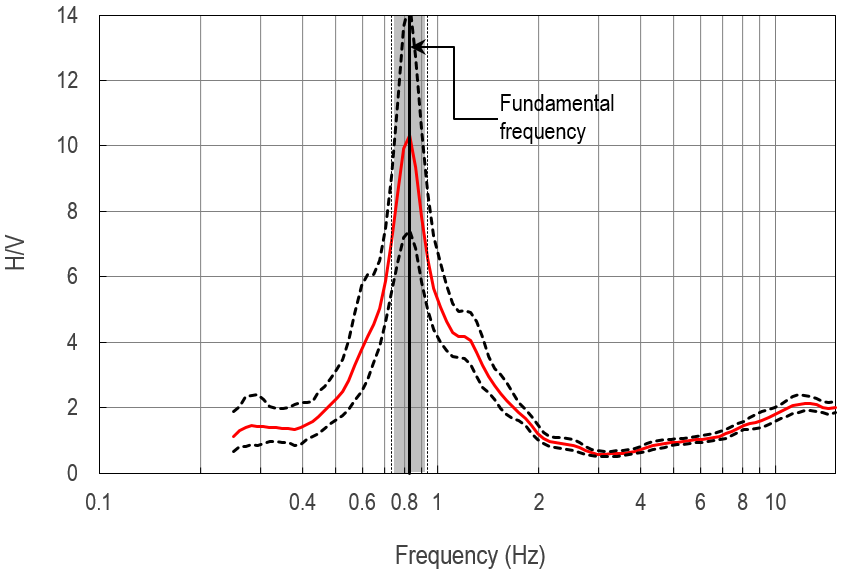 | | 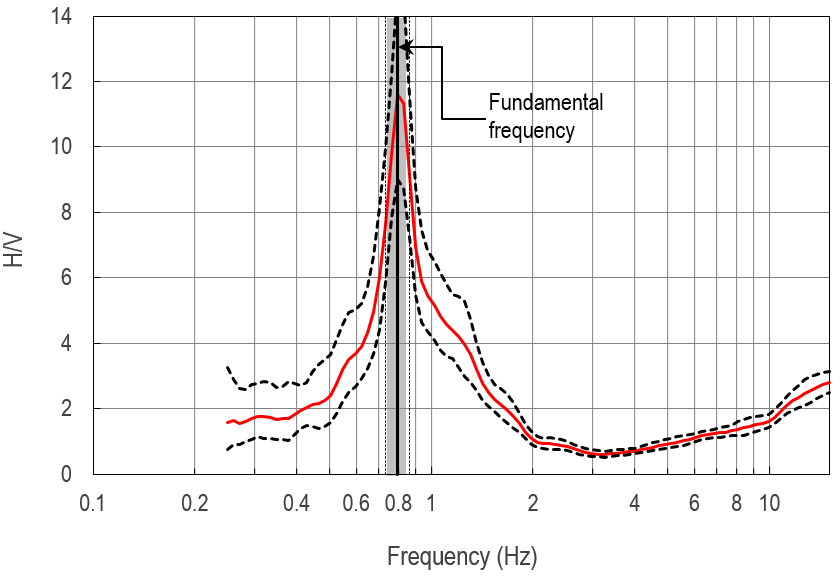 | |
| Fundamental frequency (Hz) | 0.829 | Fundamental frequency (Hz) | 0.796 |
| **Location 07 Instrument 01 Trial#05** | | **Location 07 Instrument 02 Trial#01** | |
| 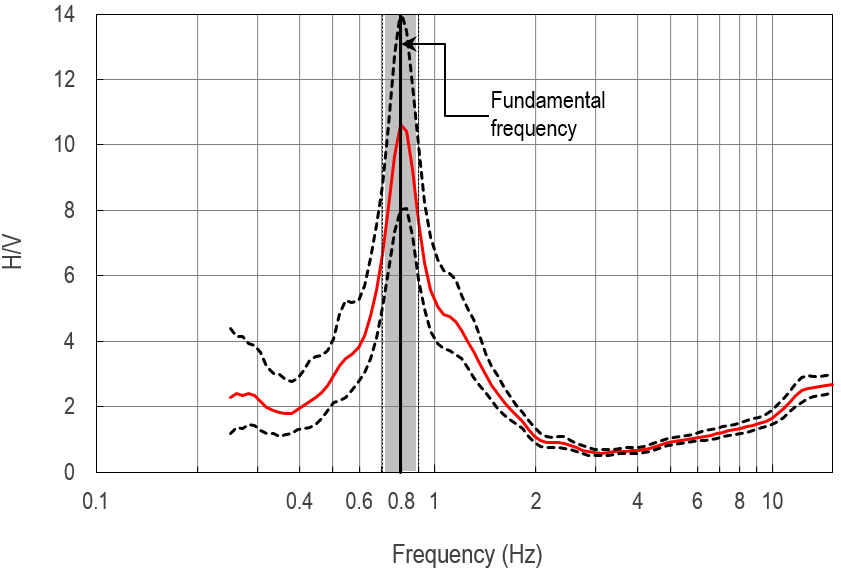 | | 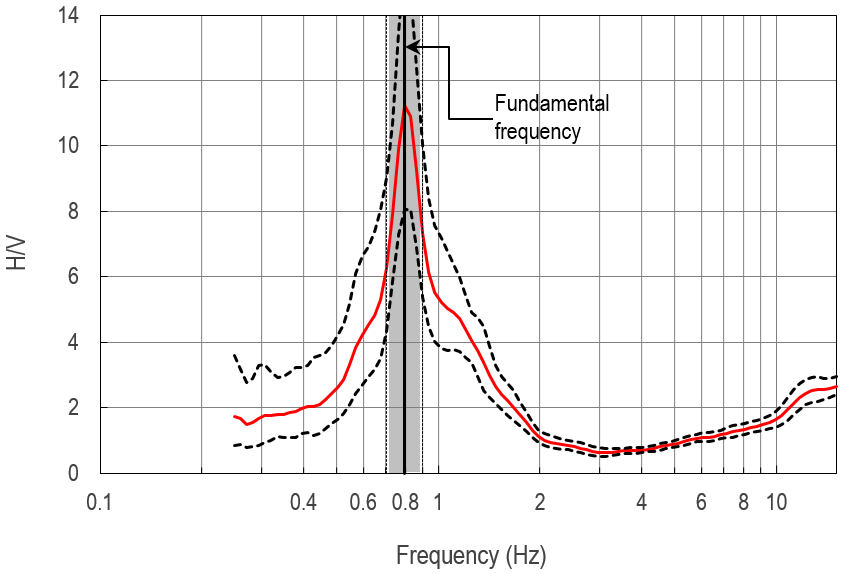 | |
| Fundamental frequency (Hz) | 0.796 | Fundamental frequency (Hz) | 0.796 |
| **Location 07 Instrument 02 Trial#02** | | **Location 07 Instrument 02 Trial#03** | |

| 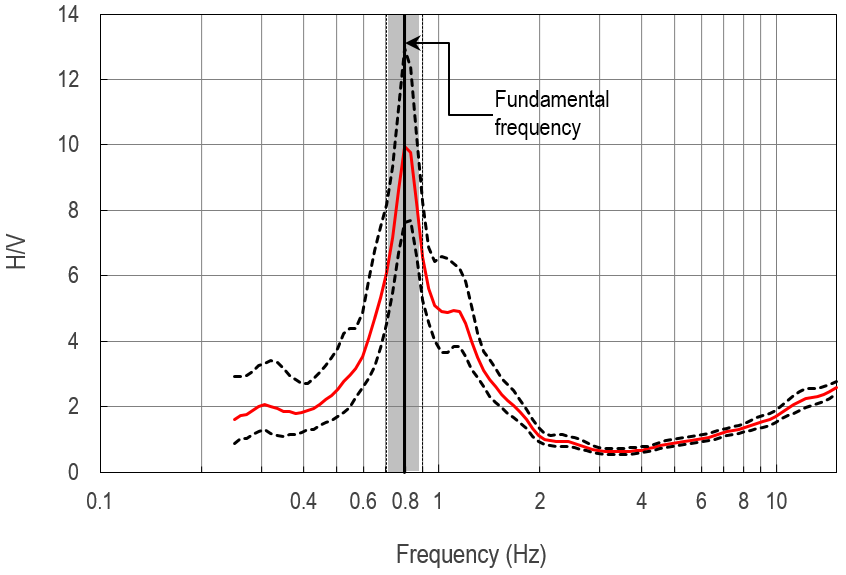 | | 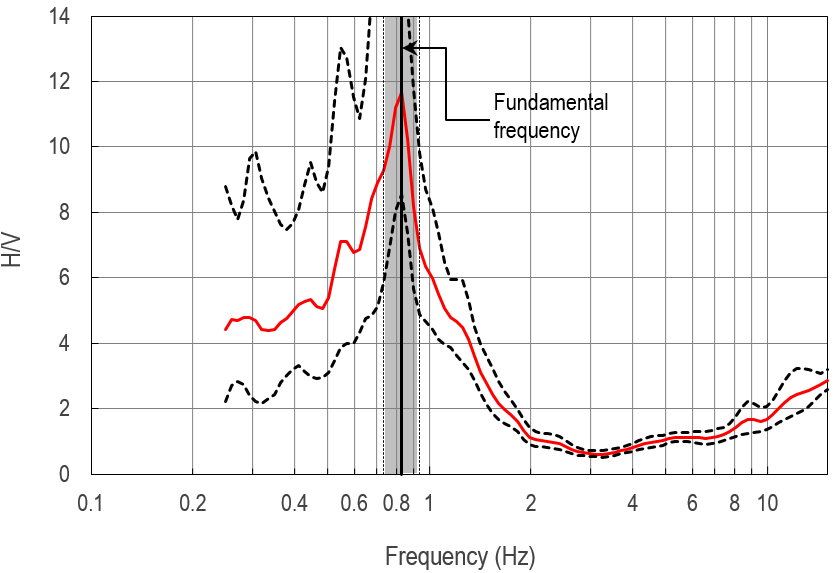 | |
| --- | --- | --- | --- |
| Fundamental frequency (Hz) | 0.796 | Fundamental frequency (Hz) | 0.829 |
| **Location 07 Instrument 02 Trial#04** | | **Location 07 Instrument 03 Trial#01** | |
| 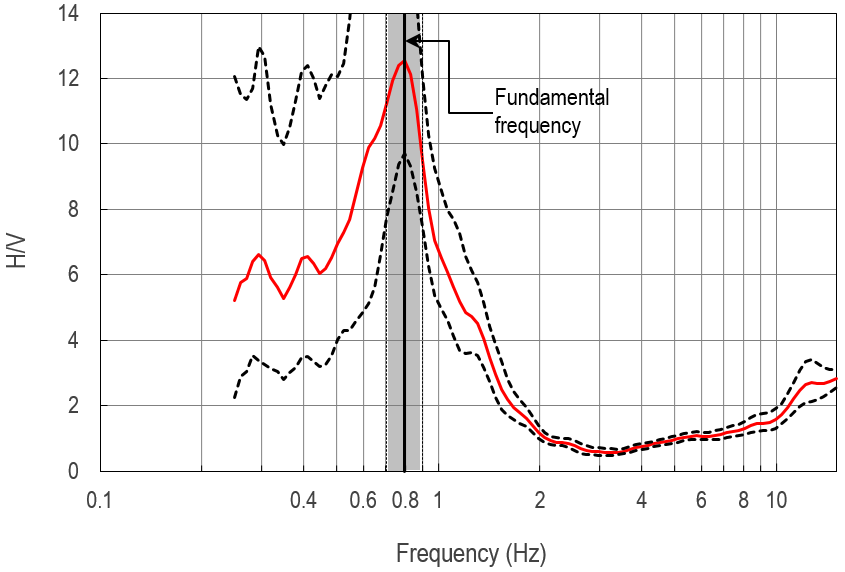 | | 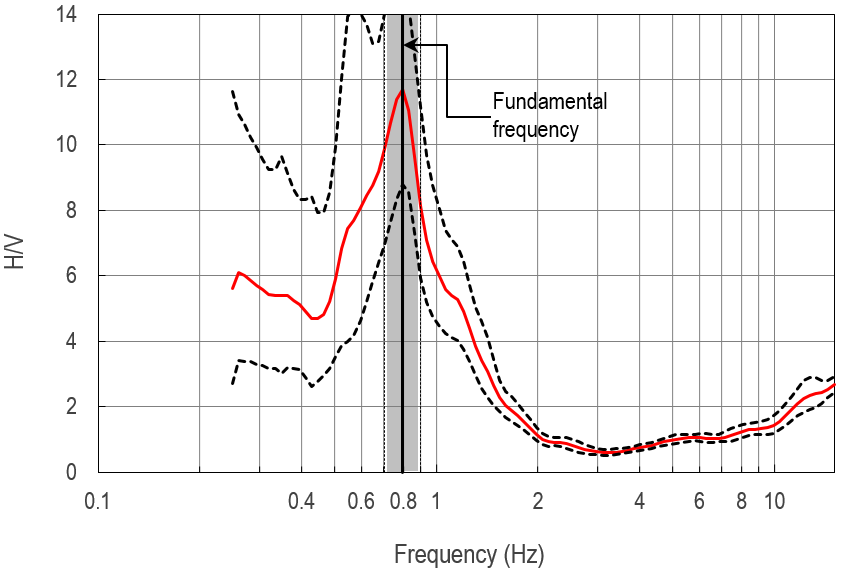 | |
| Fundamental frequency (Hz) | 0.796 | Fundamental frequency (Hz) | 0.796 |
| **Location 07 Instrument 03 Trial#02** | | **Location 07 Instrument 03 Trial#03** | |
| 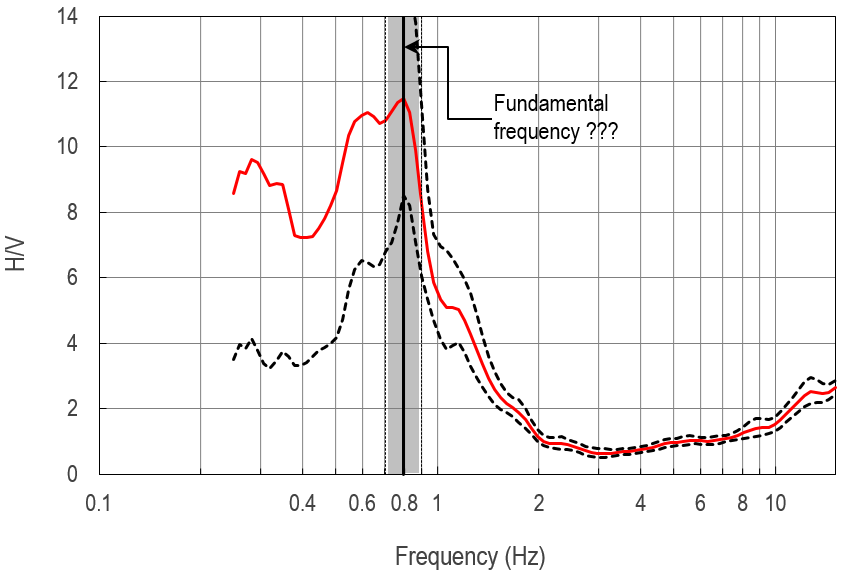 | | 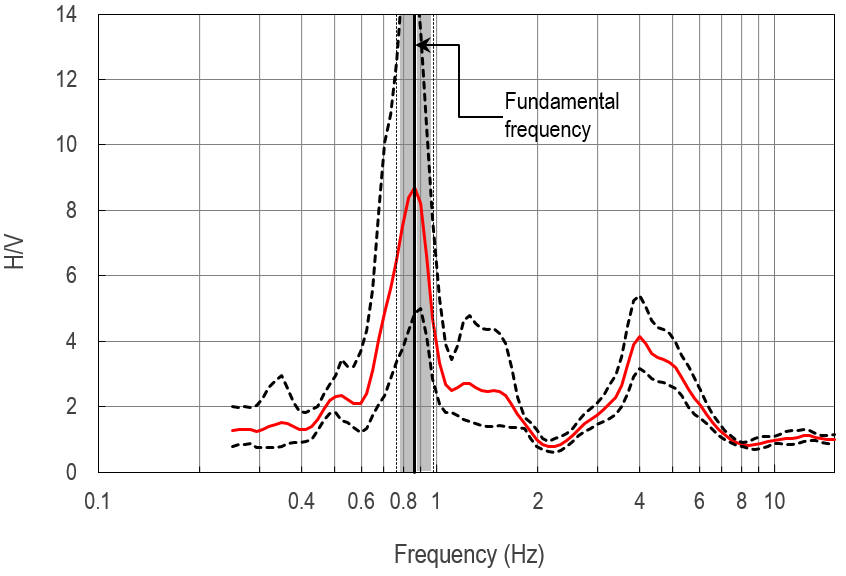 | |
| Fundamental frequency (Hz) | 0.796??? | Fundamental frequency (Hz) | 0.865 |
| **Location 07 Instrument 03 Trial#04** | | **Location 08 Instrument 01 Trial#01** | |

| 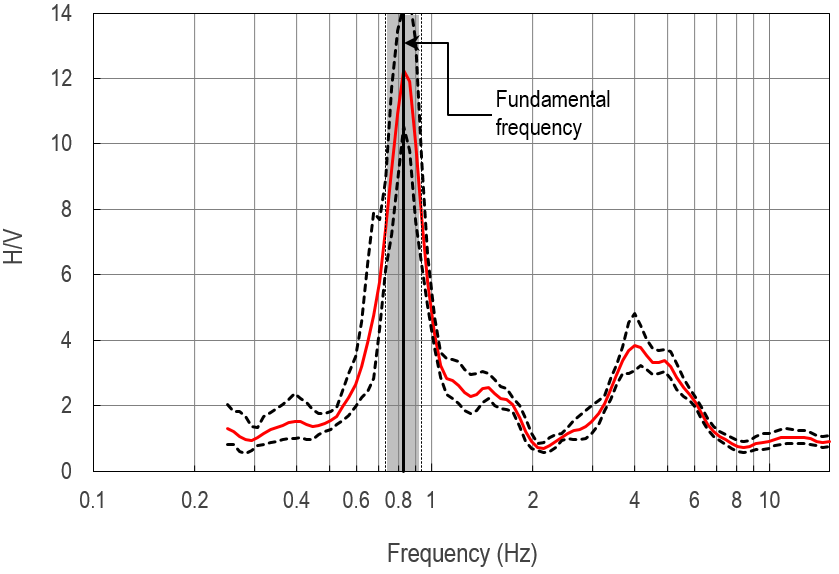 | | 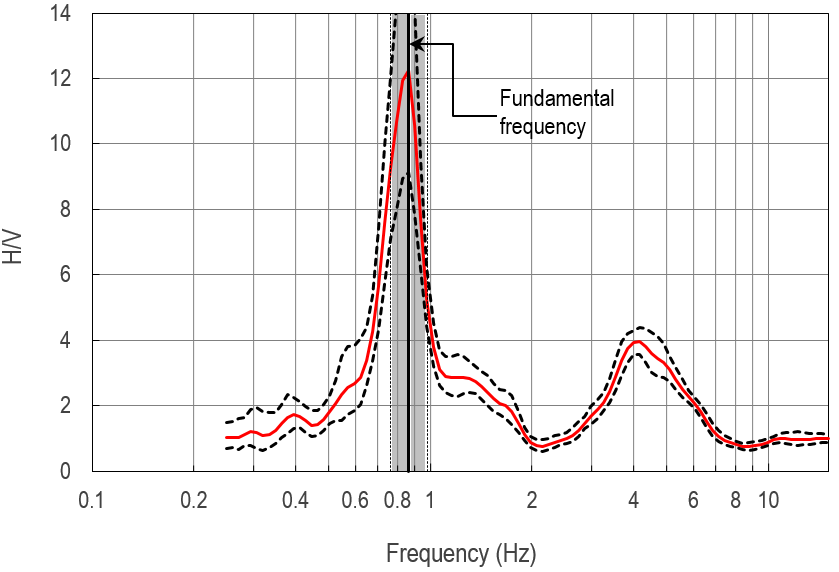 | |
| --- | --- | --- | --- |
| Fundamental frequency (Hz) | 0.829 | Fundamental frequency (Hz) | 0.865 |
| **Location 08 Instrument 01 Trial#02** | | **Location 08 Instrument 01 Trial#03** | |
| 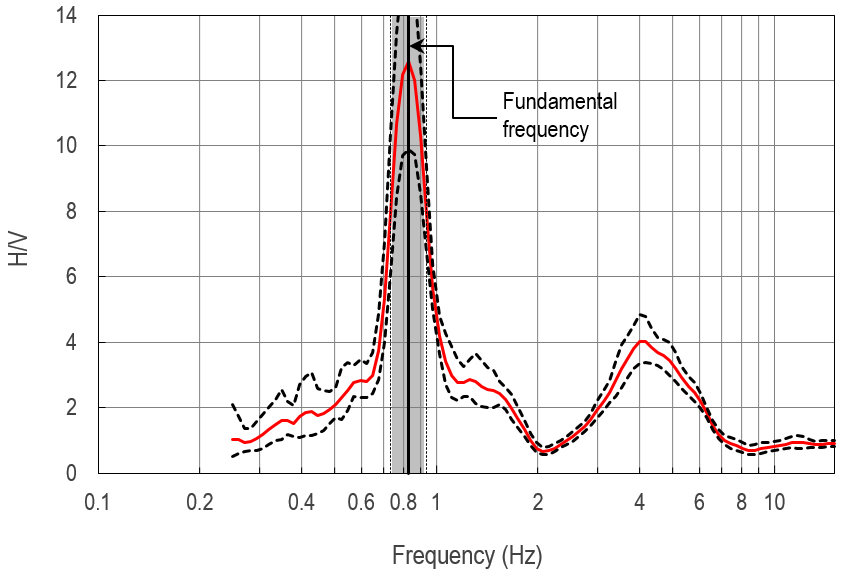 | | 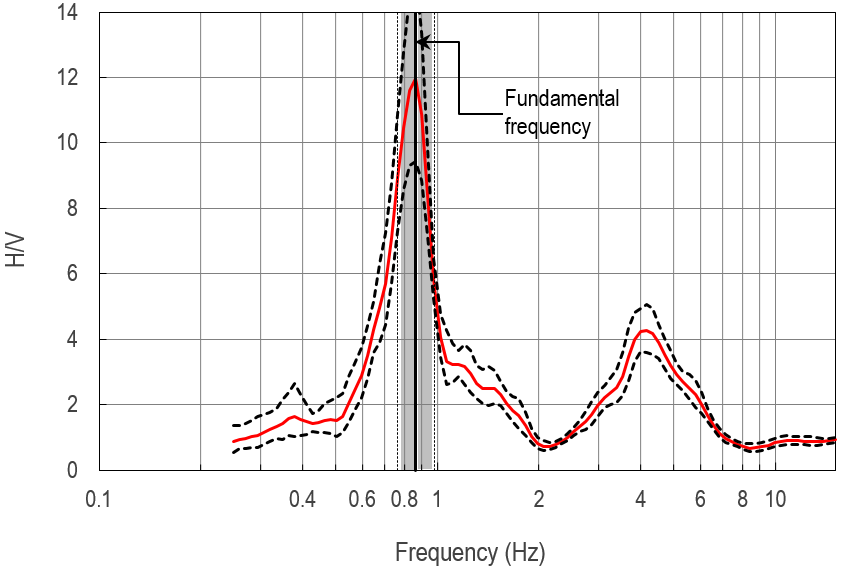 | |
| Fundamental frequency (Hz) | 0.829 | Fundamental frequency (Hz) | 0.865 |
| **Location 08 Instrument 01 Trial#04** | | **Location 08 Instrument 01 Trial#05** | |
| 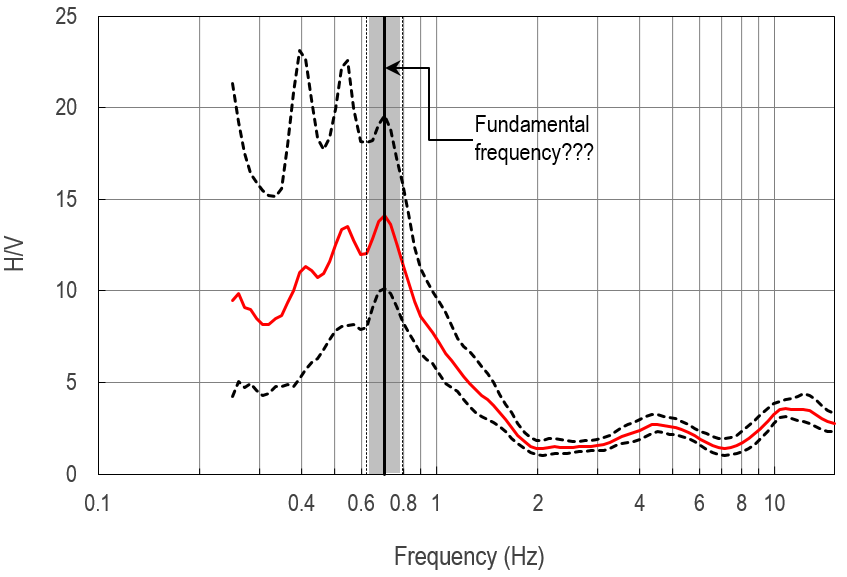 | | 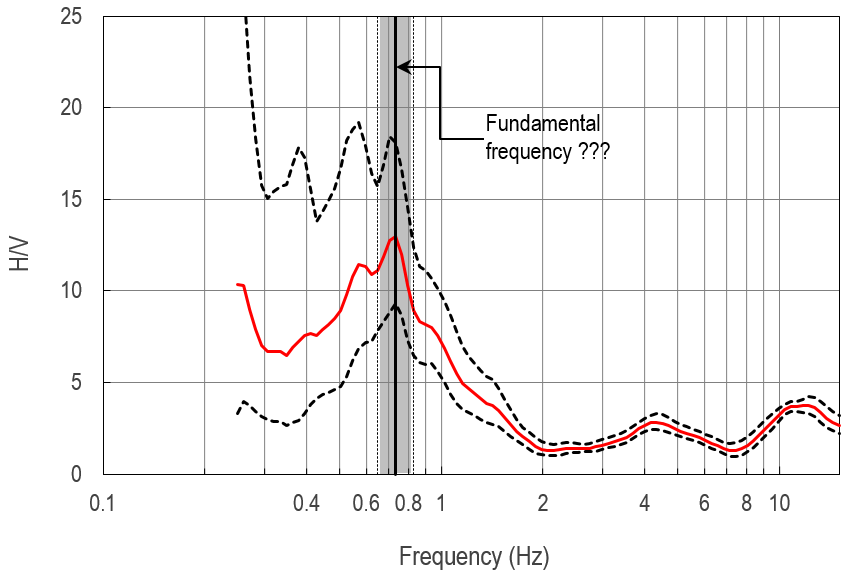 | |
| Fundamental frequency (Hz) | 0.703??? | Fundamental frequency (Hz) | 0.733??? |
| **Location 09 Instrument 01 Trial#01** | | **Location 09 Instrument 01 Trial#02** | |

| 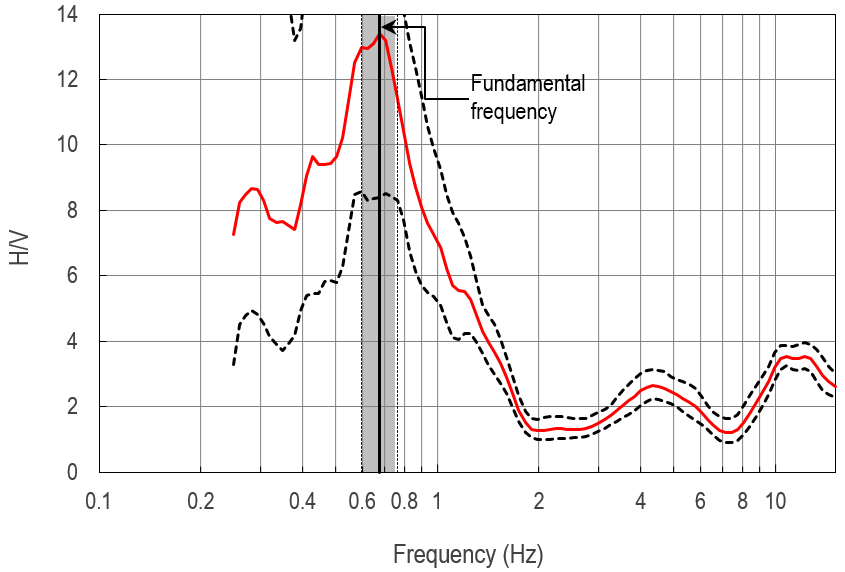 | | 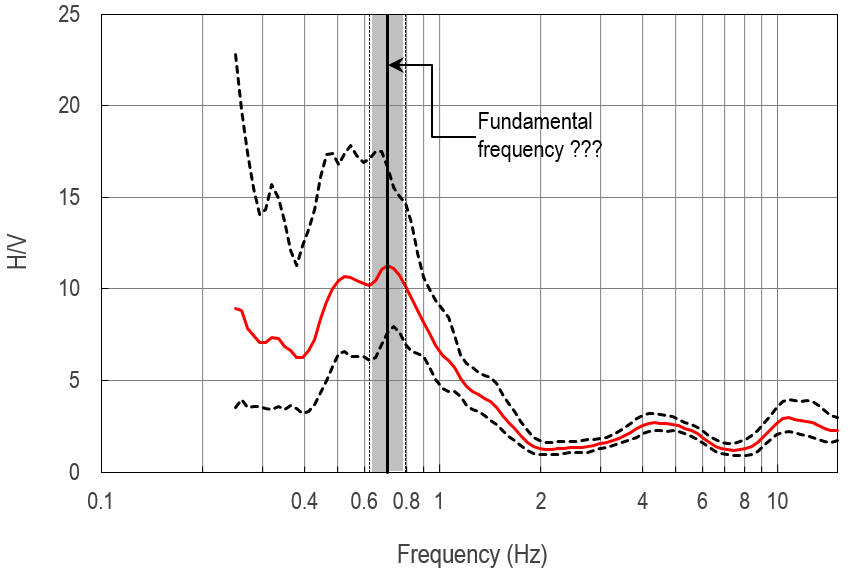 | |
| --- | --- | --- | --- |
| Fundamental frequency (Hz) | 0.675 | Fundamental frequency (Hz) | 0.703??? |
| **Location 09 Instrument 01 Trial#03** | | **Location 09 Instrument 01 Trial#04** | |
| 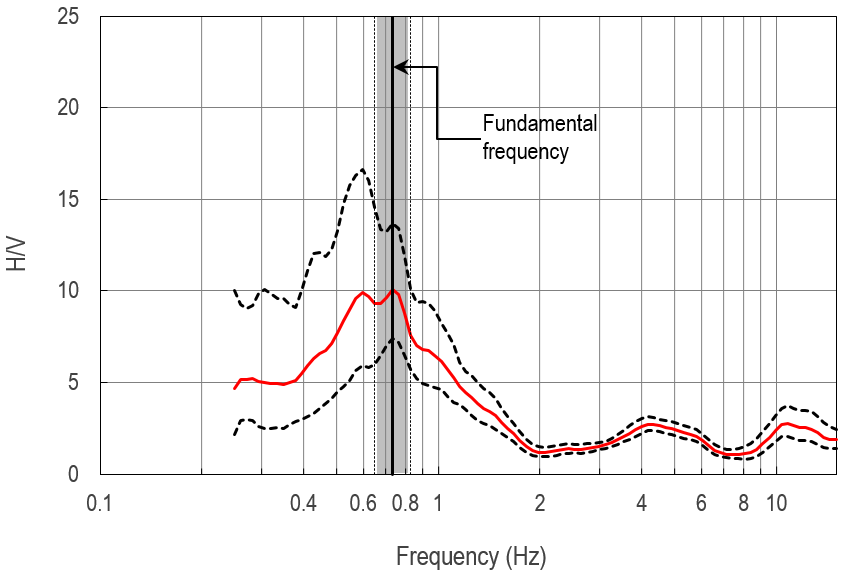 | | 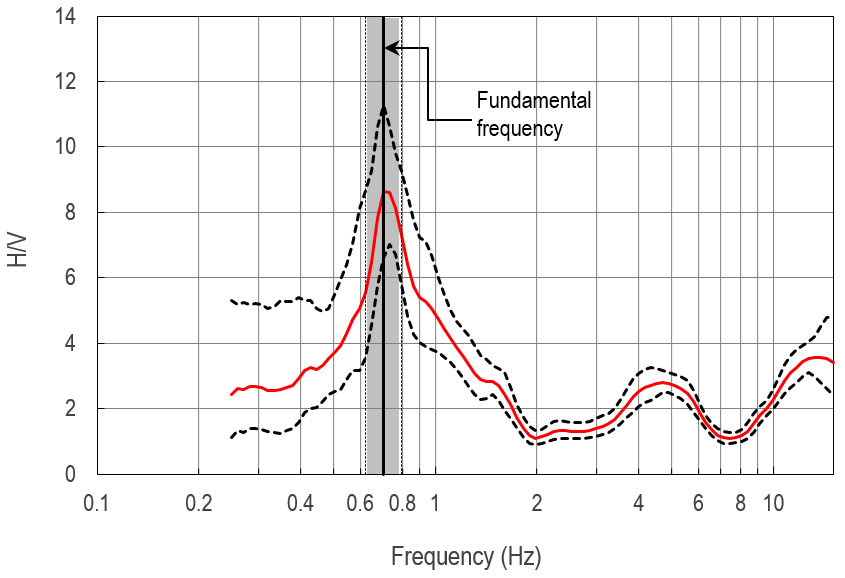 | |
| Fundamental frequency (Hz) | 0.733 | Fundamental frequency (Hz) | 0.703 |
| **Location 09 Instrument 01 Trial#05** | | **Location 09 Instrument 02 Trial#01** | |
|  | |  | |
| Fundamental frequency (Hz) | 0.733 | Fundamental frequency (Hz) | 0.733??? |
| **Location 09 Instrument 02 Trial#02** | | **Location 09 Instrument 02 Trial#03** | |

|  | |  | |
| --- | --- | --- | --- |
| Fundamental frequency (Hz) | 0.733 | Fundamental frequency (Hz) | 0.733 |
| **Location 09 Instrument 02 Trial#04** | | **Location 09 Instrument 03 Trial#01** | |
|  | |  | |
| Fundamental frequency (Hz) | 0.733 | Fundamental frequency (Hz) | 0.733 |
| **Location 09 Instrument 03 Trial#02** | | **Location 09 Instrument 03 Trial#03** | |
|  | |  | |
| Fundamental frequency (Hz) | 0.733 | Fundamental frequency (Hz) | 0.733 |
| **Location 09 Instrument 03 Trial#04** | | **Location 09 Instrument 03 Trial#05** | |

|  | |  | |
| --- | --- | --- | --- |
| Fundamental frequency (Hz) | 0.764??? | Fundamental frequency (Hz) | 0.596??? |
| **Location 10 Instrument 01 Trial#01** | | **Location 10 Instrument 01 Trial#02** | |
|  | |  | |
| Fundamental frequency (Hz) | 0.796??? | Fundamental frequency (Hz) | 0.796??? |
| **Location 10 Instrument 01 Trial#03** | | **Location 10 Instrument 01 Trial#04** | |
|  | |  | |
| Fundamental frequency (Hz) | 0.596??? | Fundamental frequency (Hz) | 0.572??? |
| **Location 10 Instrument 02 Trial#01** | | **Location 10 Instrument 02 Trial#02** | |

|  | |  | |
| --- | --- | --- | --- |
| Fundamental frequency (Hz) | 0.596??? | Fundamental frequency (Hz) | 0.647??? |
| **Location 10 Instrument 02 Trial#03** | | **Location 10 Instrument 02 Trial#04** | |
|  | |  | |
| Fundamental frequency (Hz) | 0.796 | Fundamental frequency (Hz) | 0.829 |
| **Location 10 Instrument 03 Trial#01** | | **Location 10 Instrument 03 Trial#02** | |
|  | |  | |
| Fundamental frequency (Hz) | 0.829 | Fundamental frequency (Hz) | 0.829 |
| **Location 10 Instrument 03 Trial#03** | | **Location 10 Instrument 03 Trial#04** | |
